# Barriers to Deprescribing Benzodiazepines in Older Adults in a Survey of European Physicians

**DOI:** 10.1001/jamanetworkopen.2024.59883

**Published:** 2025-03-03

**Authors:** Vladyslav Shapoval, Marie de Saint Hubert, Perrine Evrard, François-Xavier Sibille, Carole E. Aubert, Lucy Bolt, Vagioula Tsoutsi, Pinelopi Kollia, Antoni Salvà, Ramon Miralles, Adam Wichniak, Katarzyna Gustavsson, Torgeir Bruun Wyller, Enrico Callegari, Jeremy M. Grimshaw, Justin Presseau, Séverine Henrard, Anne Spinewine

**Affiliations:** 1 Clinical Pharmacy and Pharmacoepidemiology Research Group, Louvain Drug Research Institute, Université catholique de Louvain (UCLouvain), Brussels, Belgium; 2 Department of Geriatric Medicine, CHU UCLouvain Namur, Yvoir, Belgium; 3 Institute of Health and Society (IRSS), UCLouvain, Brussels, Belgium; 4 Department of General Internal Medicine, Inselspital, Bern University Hospital, University of Bern, Bern, Switzerland; 5 Institute of Primary Health Care (BIHAM), University of Bern, Bern, Switzerland; 6 Sleep Research Unit, First Department of Psychiatry, Eginition Hospital, Medical School, National & Kapodistrian University of Athens, Athens, Greece; 7 Fundació Salut i Envelliment UAB Universitat Autonoma de Barcelona, Barcelona, Spain; 8 Hospital Universitari Germans Trias i Pujol, Badalona, Barcelona, Spain; 9 Department of Clinical Neurophysiology, Sleep Medicine Center, Institute of Psychiatry and Neurology, Warsaw, Poland; 10 Third Department of Psychiatry, Institute of Psychiatry and Neurology, Warsaw, Poland; 11 Department of Science and Evaluation, Medical Research Agency, Warsaw, Poland; 12 Department of Geriatric Medicine, Oslo University Hospital, Oslo, Norway; 13 Institute of Clinical Medicine, University of Oslo, Oslo, Norway; 14 Department of Old Age Psychiatry, Østfold Hospital Trust, Grålum, Norway; 15 Methodological and Implementation Research, Ottawa Hospital Research Institute, Ottawa, Ontario, Canada; 16 Pharmacy Department, CHU UCLouvain Namur, Yvoir, Belgium; 17 School of Epidemiology and Public Health, University of Ottawa, Ottawa, Ontario, Canada; 18 Department of Medicine, University of Ottawa, Ottawa, Ontario, Canada

## Abstract

**Importance:**

The use of benzodiazepine receptor agonists (BZRA) poses serious health risks to older adults. Although several guidelines recommend deprescribing, implementation in clinical practice remains limited.

**Objective:**

To identify physicians’ barriers to and enablers of deprescribing BZRA in adults aged 65 years and older taking a BZRA for sleep problems; to determine factors associated with hospital physicians’ intention to deprescribe BZRA and their self-reported routine BZRA deprescribing.

**Design, Setting, and Participants:**

This survey study included hospital physicians and general practitioners (GPs) working across 6 European Countries (Belgium, Greece, Norway, Poland, Spain, and Switzerland) between December 2022 and March 2023.

**Main Outcomes and Measures:**

Barriers identification via a 35-item questionnaire based upon the Theoretical Domains Framework (TDF). Responses were categorized as major barriers, moderate barriers, and enablers based on their mean scores. Multivariable logistic regressions were used to identify background characteristics and TDF-based domains associated with hospital physicians’ intention to deprescribe and self-reported routine deprescribing.

**Results:**

Questionnaires from 240 hospital physicians and 96 GPs were analyzed. Most participants were women: 144 (61.0%) hospital physicians and 52 (54.2%) GPs. In terms of experience, the most common reported time in practice was less than 5 years for hospital physicians (76 [31.7%]) and between 10 and 14 years for GPs (35 [36.5%]). Most reported deprescribing BZRA routinely (135 hospital physicians [57.2%] and 66 GPs [72.5%]). Major barriers (and TDF domains) were similar for hospital physicians and GPs across the 6 countries. These barriers included: lack of training (skills), low self-efficacy (beliefs about capabilities), prioritization of other health issues (goals), frustration with the challenges of deprescribing (emotions), insufficient staff and time, absence of local policies (environmental context and resources), and reluctance from patients (social influence). Intention to deprescribe was significantly associated with country, occupation type, and 5 TDF domains: memory, attention, and decision process (odds ratio [OR], 1.70; 95% Ci, 1.22-2.40); social and/or professional role and identity (OR, 5.92; 95% CI, 3.28-11.07); beliefs about capabilities (OR, 2.35; 95% CI, 1.55-3.63); beliefs about consequences (OR, 3.00; 95% CI, 1.61-5.71); and reinforcement (OR, 1.49; 95% CI, 1.05-2.15). Routine deprescribing was significantly associated with 3 TDF domains: memory, attention, and decision processes; intentions; and emotions.

**Conclusion:**

In this theory-based survey study of physicians, physicians and general practitioners described numerous barriers to deprescribing BZRA in older adults. Our findings indicate that effective deprescribing efforts require approaches that address both reflective processes (eg, enhancing capability) and impulsive processes (eg, managing emotions).

## Key Points

**Question:**

What are the main barriers to benzodiazepine receptor agonists (BZRA) deprescribing in older adults from the perspective of physicians?

**Findings:**

In this survey study of 240 hospital physicians and 96 general practitioners (GPs) from 6 European countries, major barriers were found in 6 Theoretical Domains Framework domains: skills, beliefs about capabilities, goals, emotions, environmental context and resources, and social influence. Barriers were mostly similar across countries and between hospital physicians and GPs.

**Meaning:**

These findings suggest that different approaches to tackling reflective and impulsive processes are required to enhance the implementation of BZRA deprescribing in routine practice.

## Teaser

This survey study of hospital physicians and general practitioners in 6 European countries examines attitudes contributing to and impeding the deprescription of benzodiazepine receptor agonists to older patients.

## Introduction

The use of benzodiazepine receptor agonists (BZRA) poses serious health risks to older adults, including falls, fractures, dependence, and cognitive impairment.^1,2,3,4^ Guidelines advise against using BZRA for managing sleep problems, especially in older adults and for periods exceeding 4 weeks.^5,6,7^ However, BZRA continue to be widely prescribed, with prevalence increasing with age.^8,9,10,11^ In US older adults in 2020, BZRA were the most frequent potentially inappropriate medication prescribed.^12^ European data show that BZRA are often prescribed among community-dwelling older adults, with prescribing rates exceeding 30% in some countries.^8^ BZRA use is even higher in acute and long-term care settings.^8,13,14^ Although deprescribing BZRA is recommended,^15,16^ its implementation in clinical practice remains limited.

Identifying barriers is crucial in changing behaviors and reducing low-value care.^17,18^ Significant progress has been made in studying barriers to deprescribing, but key gaps remain. First, research on BZRA deprescribing is limited, particularly in Central and Southern European countries, despite their high BZRA consumption.^19^ Second, few multicountry studies exist, although they enable cross-country comparisons to explore how health care systems shape perceived barriers.^20,21^ Third, the perspectives of hospital stakeholders are underexplored compared with those of patients and GPs.^22^ Fourth, many studies lack theoretical support, which may hinder effective deprescribing efforts.

This study is part of the BE-SAFE project (Implementing a Patient-Centered and Evidence-Based Intervention to Reduce Benzodiazepine and Sedative-Hypnotic Use to Improve Patient Safety and Quality of Care), funded by the European Union’s Horizon Europe program and the Swiss State Secretariat for Education, Research and Innovation. It aims to improve patient safety by initiating the deprescribing of BZRA used by older adults for sleep problems in hospital settings. The project follows the Choosing Wisely De-Implementation Framework (CWDIF),^18^ where barrier identification is essential for developing a theory-based intervention.

The present study aimed to identify barriers to and enablers of BZRA deprescribing in older adults, as perceived by physicians. Additionally, we sought to determine factors associated with physicians’ self-reported BZRA deprescribing. The results will be used to develop a theory-based intervention that will be tested in a cluster-randomized clinical trial.

## Methods

### Study Design and Sample

We conducted a survey with hospital physicians and general practitioners (GPs) across 6 European countries (Belgium, Greece, Norway, Poland, Spain, and Switzerland). This report follows the American Association for Public Opinion Research (AAPOR) reporting guideline for survey research and the Consensus-Based Checklist for Reporting of Survey Studies (CROSS) criteria.

Eligible hospital physicians worked in acute medical wards or outpatient clinics caring for older adults in one of the hospitals participating in BE-SAFE (from 1 to 3 hospitals per country). We excluded wards and clinics with existing structured BZRA deprescribing processes. Eligible GPs had to practice within the same regional area as the hospitals.

We aimed for a target sample of 40 hospital physicians and 20 GPs in each country, totaling 240 hospital physicians and 120 GPs. This sample size was deemed sufficient to guide BE-SAFE intervention development, feasible within project timelines, and suitable for cross-country comparisons. We chose a larger sample of hospital physicians since they constitute the primary target for the future BE-SAFE intervention.

The study followed the Declaration of Helsinki principles and was approved by local ethical committees for each region.^23^ All participants provided written informed consent.

### Survey Questionnaire

The questionnaire was based on the Theoretical Domains Framework (TDF) version 2, which maps 128 constructs from 33 behavior change theories into 14 domains that reflect determinants of behavior. The TDF is widely used to evaluate barriers and enablers.^24,25^ We developed items from barriers identified in a TDF-based systematic review on BZRA deprescribing^22^ and 2 validated TDF-based questionnaires.^26,27^ Items were refined with input from experts in implementation and behavioral sciences, deprescribing, geriatrics, and sleep medicine.

The targeted behavior was BZRA deprescribing for adults aged 65 years and older, after taking a BZRA for sleep problems, initiated by a hospital physician and followed by a GP. Deprescribing was defined as the process of tapering and stopping drugs, aimed at minimizing polypharmacy and improving patient outcomes.^28^ This includes subactions such as checking eligibility to deprescribe, shared decision-making, tapering, selecting support measures, and following up.

The final version of the questionnaire comprises 44 questions, including 5 background questions, 35 TDF-based items, 2 questions on self-reported behavior (ie, whether they have ever deprescribed a BZRA and whether they routinely consider BZRA deprescription), and 2 open questions for additional insights. The 35 items span 12 TDF domains (knowledge; skills; memory, attention, and decision processes; social and/or professional role and identity; beliefs about capabilities; beliefs about consequences; reinforcement; goals; intentions; emotions; environmental context and resources; and social influence) and are measured on a 5-point Likert scale. We excluded 2 TDF domains—optimism and behavioral regulation—due to the lack of relevant barriers in previous research. The GP questionnaire was similar to the version for hospital physicians, with differences in 3 items related to social and/or professional role and identity and the environmental context and resources TDF domains.

Translation from English into 6 languages (French, German, Greek, Norwegian, Polish, and Spanish) followed the forward-backward methodology.^29,30^ Pilot testing involved at least 2 physicians per country to ensure the questionnaire’s clarity, comprehensiveness, and acceptability. The study protocol and questionnaires are available on the Open Science Framework platform (Supplement 2).

### Survey Procedure

Recruitment occurred between December 2022 and May 2023. All eligible hospital physicians were invited via email to complete the online survey using Qualtrics version 09.22. Physicians could also complete the questionnaire on paper, with local researchers responsible for data entry into Qualtrics. GPs were invited via email, newsletters, local journals, and through local primary care contacts.

### Statistical Analysis

Respondents who only answered questions on background characteristics were excluded. We performed descriptive analyses on the full sample and by country. Post hoc, we described results by physician specialty due to variations across countries. Reversed scoring was applied to negative items so that lower scores consistently reflect higher barriers, regardless of the item wording. Barriers and enablers were classified as follows: items with a mean score below 3 were classified as major barriers, those with scores between 3 and 4 were considered moderate barriers, and items scoring 4 or higher were categorized as enablers.

We used 2 multivariable logistic regression models to identify factors (among background characteristics and TDF domains) associated with intention to deprescribe (first model) and self-reported routine deprescribing (second model). Scores were computed for TDF domains by averaging variables related to the domain. Univariate regressions were conducted for each model. For multivariable models, we selected background characteristics and TDF-based variables with *P* < .15. Stepwise logistic regression was then applied to select the best-fitting variables for each multivariable model.

Statistical analysis was performed using R version 4.2.1 (R Project for Statistical Computing) with packages tidyr and Likert. A 2-sided *P* value <.05 was considered statistically significant.

Answers were analyzed using a qualitative deductive methodology. Two researchers (V.S. and P.E.) trained in qualitative research and TDF coding independently coded the citations within the TDF domains, using NVivo version 14.23.2 (Lumivero). Disagreements were resolved with a senior researcher (A.S.).

## Results

### Population Characteristics

The final sample comprised 240 hospital physicians and 96 GPs. Most participants were women (144 hospital physicians [61.0%] and 52 GPs [54.2%]) (Table 1). Hospital physicians mainly included internal medicine specialists (98 [40.8%]), geriatricians (44 [18.3%]), and neurologists (28 [11.7%]). One hundred eighty-four hospital physicians (78.0%) had previously deprescribed BZRA in older adults with sleep problems, and 135 (57.2%) reported deprescribing BZRA routinely. For GPs, the results were 82 (90.1%) and 66 (72.5%), respectively. Detailed participant characteristics are presented by country in eTables 1 and 2 in Supplement 1.

**Table 1.** Background Characteristics of Hospital Physicians and General Practitioners

Characteristics	Respondents, No. (%)^a^	
	Hospital physicians (n = 240)	GPs (n = 96)
Country		
Belgium	43 (17.9)	20 (20.8)
Greece	40 (16.6)	19 (19.8)
Norway	37 (15.7)	2 (2.2)
Poland	40 (16.6)	19 (19.8)
Spain	40 (16.6)	16 (16.6)
Switzerland	40 (16.6)	20 (20.8)
Age, y		
≤30	65 (27.1)	5 (5.2)
31-40	63 (26.2)	28 (29.2)
41-50	64 (26.7)	28 (29.2)
51-60	31 (12.9)	27 (28.1)
≥61	17 (7.1)	8 (8.3)
Gender		
Men	91 (38.6)	44 (45.8)
Women	144 (61.0)	52 (54.2)
Prefer not to answer	1 (0.4)	0
Occupation (hospital physicians)		
Other	40 (16.7)	NA
Cardiologist	20 (8.3)	
Geriatrician	44 (18.3)	
Internist	98 (40.8)	
Neurologist	28 (11.7)	
Psychiatrist	10 (4.2)	
Other^b^	40 (16.7)	
Type of care (hospital physicians)		
Inpatients	111 (46.6)	NA
Outpatients (seen in clinics)	33 (13.9)	
Both (inpatients/outpatients)	94 (39.5)	
Type of practice (GPs)		
Solo practice (in the office on my own)	NA	21 (22.3)
Group practice, monodisciplinary		20 (21.3)
Group practice, multidisciplinary (GPs and nonphysicians)		23 (24.5)
Group practice, multidisciplinary (GPs, specialists, and nonphysicians)		30 (31.9)
Experience, y		
<5	76 (31.7)	13 (13.5)
5-9	42 (17.5)	14 (14.6)
10-14	29 (12.1)	35 (36.5)
15-19	30 (12.5)	23 (24.0)
≥20	63 (26.2)	11 (11.5)
Deprescribed BZRA before, self-reported		
Yes	184 (78.0)	82 (90.1)
No	52 (22.0)	9 (9.9)
Deprescribe BZRA routinely, self-reported		
Yes	135 (57.2)	66 (72.5)
No	101 (42.7)	25 (27.5)

Abbreviations: BZRA, benzodiazepine receptor agonists; GPs, general practitioners; NA, not applicable.

^a^ Missing response (excluded from percentages) totaled 4 for gender (hospital physicians), 2 for type of practice (GPs), 2 for type of care (hospital physicians), 4 for deprescribe before (hospital physicians), 5 for deprescribed before (GPs), 4 for deprescribe routinely (hospital physicians), 5 for deprescribe routinely (GPs).

^b^ Others physicians include: pneumologist (2 respondents), physical medicine and rehabilitation physician (3 respondents), surgeon (4 respondents), emergency medicine (3 respondents), palliative medicine (1 respondent), oncologist (1 respondent), hematologist (1 respondent), rheumatologist (1 respondent), and not specified (24 respondents).

#### Barriers and Enablers for Hospital Physicians

For hospital physicians, we identified 2 enablers in 2 TDF domains. Most physicians reported that they know the risks associated with the BZRA use (what we describe as knowledge) and believe that the benefits of deprescribing outweigh the risks (beliefs about consequences). In contrast, 12 items across 6 TDF domains were major barriers. Most participants reported a lack of training in deprescribing BZRA, engaging patients in discussions about deprescribing, and implementing alternative approaches (skills). Most hospital physicians believe they are unable to deprescribe BZRA due to time constraints (beliefs about capabilities, environmental context, and resources), believe their patients have more important health issues to address than BZRA deprescribing (goals), and feel frustrated with the challenges of BZRA deprescribing (emotions). Four major barriers emerged within the environmental context and resources domain: lack of staff, lack of time, the perception that deprescribing is not a priority at the hospital level, and the lack of local policies to encourage BZRA deprescribing. Finally, we identified 2 major barriers in the domain of social influence: most participants reported pressure from patients to continue their BZRA and noted that most patients are reluctant to discontinue their BZRA. The remaining 20 items were moderate barriers across 10 TDF domains (Table 2).

**Table 2.** Barriers to and Enablers of BZRA Deprescribing by TDF-Based Items for Hospital Physicians and General Practitioners

TDF-based domains and items	Hospital physicians (n = 240)		General practitioners (n = 96)	
	Mean (SD)	Classification^a^	Mean (SD)	Classification^a^
**Knowledge**				
I know the risks associated with the use of BZRA in older adults with sleep problems.	4.32 (0.77)	Enabler	4.52 (0.54)	Enabler
I know the situations or comorbidities in which BZRA deprescribing is not recommended.	3.39 (1.06)	Moderate barrier	3.36 (0.99)	Moderate barrier
I know how to taper BZRA in older adults with sleep problems.	3.33 (1.11)	Moderate barrier	3.76 (0.86)	Moderate barrier
I know how to engage patients about BZRA deprescribing.	3.38 (0.96)	Moderate barrier	3.78 (0.86)	Moderate barrier
I am aware of alternative approaches to deal with sleep problems in older adults.	3.74 (0.97)	Moderate barrier	3.76 (0.83)	Moderate barrier
**Skills**				
I have been trained on how to deprescribe BZRA in older adults with sleep problems.	2.74 (1.21)	Major barrier	2.86 (1.26)	Major barrier
I have been trained to engage patients about deprescribing their BZRA.	2.67 (1.19)	Major barrier	2.60 (1.15)	Major barrier
I have been trained to implement alternative approaches for sleep problems in older adults.	2.82 (1.21)	Major barrier	2.82 (1.19)	Major barrier
**Memory, attention, and decision processes**				
I usually do not consider BZRA deprescribing in older adults with sleep problems because it is a difficult and time-consuming process.	3.25 (1.02)^b^	Moderate barrier	3.55 (1.07)^b^	Moderate barrier
As long as the patient has no specific issue or request, I renew/continue the prescription of BZRA.	3.37 (1.00)^b^	Moderate barrier	3.29 (1.07)^b^	Moderate barrier
**Social and/or professional role and identity**				
It is my responsibility as a physician (general practitioner) to deprescribe BZRA in older adults with sleep problems.	3.77 (0.96)	Moderate barrier	4.05 (0.97)	Enabler
In the department or institution where I work, it is relevant to initiate BZRA deprescribing.^c^	3.48 (1.06)	Moderate barrier	NA	NA
In my practice, it is relevant to follow up on BZRA deprescribing initiated by a physician from the hospital.^d^	NA	NA	4.01 (1.02)	Enabler
I don’t feel concerned with the BZRA deprescribing in older adults.	3.70 (1.01)^b^	Moderate barrier	4.10 (1.01)^b^	Enabler
**Beliefs about capabilities**				
I am confident that I can deprescribe BZRA in older adults with sleep problems even when I have limited time.	2.83 (1.04)	Major barrier	2.74 (0.99)	Major barrier
I am confident I could deprescribe BZRA in older adults with sleep problems if I wanted to.	3.30 (0.99)	Moderate barrier	3.11 (0.97)	Moderate barrier
**Beliefs about consequences**				
If I deprescribe BZRA in older adults with sleep problems, it will benefit the population’s health in general.	3.88 (0.85)	Moderate barrier	3.90 (0.89)	Moderate barrier
If I deprescribe BZRA in older adults with sleep problems, it will negatively affect my relationship with these patients.	3.49 (0.88)^b^	Moderate barrier	3.38 (0.97)^b^	Moderate barrier
I believe that BZRA deprescribing in older adults with sleep problems will have negative consequences for my patients’ health.	3.40 (0.88)^b^	Moderate barrier	3.42 (1.04)^b^	Moderate barrier
In general, I believe that the benefits of deprescribing BZRA in older adults with sleep problems outweigh the harms.	4.00 (0.88)	Enabler	3.95 (0.97)	Moderate barrier
**Reinforcement**				
I am reluctant to deprescribe BZRA in older adults with sleep problems due to previous failed attempts.	3.44 (0.92)^b^	Moderate barrier	3.20 (1.14)	Moderate barrier
**Goals**				
BZRA deprescribing in older adults with sleep problems is a priority for me.	3.13 (0.99)	Moderate barrier	3.08 (0.99)	Moderate barrier
My patients often have other health problems that are usually more important for me to address than the BZRA deprescribing.	2.29 (0.96)^b^	Major barrier	3.43 (0.92)^b^	Moderate barrier
**Intentions**				
I intend to deprescribe BZRA in older adults with sleep problems.	3.66 (0.86)	Moderate barrier	3.84 (0.88)	Moderate barrier
I intend to promote alternative approaches to help older adults deal with sleep problems.	3.78 (0.91)	Moderate barrier	3.88 (0.91)	Moderate barrier
**Emotions**				
I feel frustrated with all the challenges of the BZRA deprescribing in older adults with sleep problems.	2.87 (1.04)^b^	Major barrier	2.56 (0.991)^b^	Major barrier
I feel stressed about deprescribing BZRA in older adults with sleep problems.	3.07 (1.03)^b^	Moderate barrier	3.04 (1.15)^b^	Moderate barrier
**Environmental context and resources**				
There are guidelines and tools available for deprescribing BZRA in older adults with sleep problems that are possible to implement in my practice.	3.00 (0.95)	Moderate barrier	3.22 (0.89)	Moderate barrier
I have enough time to educate and inform patients about the BZRA deprescribing.	2.44 (1.06)	Major barrier	2.51 (1.19)	Major barrier
There is enough staff in the department or institution where I work to support BZRA deprescribing.^c^	2.77 (1.11)	Major barrier	NA	NA
In my practice, there are enough collaborators to support BZRA deprescribing.^d^	NA	NA	2.33 (1.03)	Major barrier
In my opinion, BZRA deprescribing is not prioritized by our health care system.	2.69 (1.20)^b^	Major barrier	3.09 (1.24)^b^	Moderate barrier
In the department or institution where I work, we have set goals (or policies) that encourage BZRA deprescribing.^c^	2.69 (1.08)	Major barrier	NA	NA
For general practitioners, in my area or my region, goals or policies have been set that encourage BZRA deprescribing.^d^	NA	NA	2.30 (1.17)	Major barrier
**Social influence, colleagues**				
My colleagues/collaborators whose opinions I value support the BZRA deprescribing in older adults with sleep problems.	3.70 (0.90)	Moderate barrier	3.46 (0.94)	Moderate barrier
My colleagues are supportive of BZRA deprescribing.	3.61 (0.97)	Moderate barrier	3.54 (0.93)	Moderate barrier
**Social influence, patients**				
I feel a lot of pressure from older adults with sleep problems and/or their relatives to renew/extend their prescriptions.	2.35 (0.92)^b^	Major barrier	1.65 (0.68)^b^	Major barrier
Most of my older patients taking a BZRA for sleep problems or their relatives are reluctant to deprescribe their BZRA.	2.18 (0.82)^b^	Major barrier	1.65 (0.62)^b^	Major barrier

Abbreviations: BZRA, benzodiazepine receptor agonists; NA, not applicable; TDF, Theoretical Domains Frameworks.

^a^ Items are measured on a 5-point Likert scale from 1 (“Strongly disagree”) to 5 (“Strongly agree”). Items with mean scores of 4 or higher were classified as enablers, 3-4 as moderate barriers, and below 3 as major barriers.

^b^ Reversed scoring so that lower scores reflect higher barriers for all items, irrespective of item wording.

^c^ Item for hospital physicians.

^d^ Item for general practitioners.

Most of the major barriers were similar across countries and specialties, although we observed differences across some specialties (eTable 3 in Supplement 1). For example, psychiatrists and geriatricians rated items higher in knowledge, skills, beliefs about capabilities, and intention domains.

In free-text answers, the most cited barriers to deprescribing were in environmental context and resources (insufficient time, short hospital inpatient stays, inadequate follow-up, poor coordination between inpatient and outpatient care, and limited support for deprescribing) and social influences (patient reluctance, lack of cooperation among physicians, and nursing staff resistance). Additionally, physicians noted that patients’ acute conditions, psychiatric issues, and cognitive impairments further complicated deprescribing efforts (beliefs about capabilities) (eAppendices 1 and 2 in Supplement 1).

#### Barriers and Enablers for General Practitioners

Overall, GPs’ results were similar to those of hospital physicians. Ten of the 12 major barriers identified for hospital physicians were also major for GPs. The 2 remaining items were moderate barriers: the perception that their patients have more important health problems to address (goals) and that BZRA deprescribing is not prioritized by the health care system (environmental context and resources). All items were enablers in the social, professional role, and identity domains. These included GPs’ perception that deprescribing BZRA is their responsibility and that they feel concerned with BZRA deprescribing. Notably, most GPs favored continuing the BZRA deprescribing initiated by a hospital physician.

Results per country showed more barriers in Greece and Poland than in other countries and more enablers in Switzerland and Belgium. Answers to the open question on barriers mainly fell in the domain of social influence (with influence from patients and families mostly cited) and of environmental context and resources (primarily relating to the lack of or limited access to alternatives) (eTable 4 in Supplement 1).

### Factors Associated With Intention and Self-Reported Behavior in Hospital Physicians

Five TDF-based domains were significantly associated with higher odds of intention to deprescribe BZRA (Table 3): memory, attention, and decision processes (OR, 1.70; 95% CI, 1.22-2.40), social and/or professional role and identity (OR, 5.92; 95% CI, 3.28-11.07), beliefs about capabilities (OR, 2.35; 95% CI, 1.55-3.63), beliefs about consequences (OR, 3.00; 95% CI, 1.61-5.71), and reinforcement (OR, 1.49; 95% CI, 1.05-2.15). Furthermore, the odds of intending to deprescribe BZRA were lower in Spanish physicians (OR, 0.21; 95% CI, 0.06-0.69) compared with Belgian physicians; and geriatricians had higher odds (OR, 6.58; 95% CI, 2.37-19.11) than internists. Three TDF domains were significantly associated with higher odds of routinely deprescribing BZRA: memory, attention, and decision processes (OR, 2.79; 95% CI, 1.73-4.69), intentions (OR, 4.42; 95% CI, 2.38-8.83), and emotions (OR, 1.75; 95% CI, 1.04-3.04) (Table 4).

**Table 3.** Factors Associated With Self-Reported Intention to Deprescribe BZRA, in Multivariable Logistic Regression for Hospital Physicians^a^

Variables	OR (95% CI)	*P* value
Country		
Belgium	1 [Reference]	NA
Greece	0.78 (0.24-2.62)	.69
Norway	0.37 (0.11-1.28)	.12
Poland	0.84 (0.11-6.56)	.87
Spain	0.21 (0.06-0.69)^b^	<.001^b^
Switzerland	2.14 (0.62-7.61)	.23
Occupation		
Internist	1 [Reference]	NA
Cardiologist	4.28 (0.42-46.29)	.22
Geriatrician	6.58 (2.37-19.11)^b^	<.001^b^
Neurologist	1.17 (0.20-7.21)	.86
Psychiatrist	1.02 (0.22-4.98)	.97
Other	1.21 (0.45-3.23)	.71
TDF domains^c^		
Memory, attention, and decision processes	1.70 (1.22-2.40)^b^	<.001^b^
Social/professional role and identity	5.92 (3.28-11.07)^b^	<.001^b^
Beliefs about capabilities	2.35 (1.55-3.63)^b^	<.001^b^
Beliefs about consequences	3.00 (1.61-5.71)^b^	<.001^b^
Reinforcement	1.49 (1.05-2.15)^b^	.02^b^

Abbreviations: BZRA, benzodiazepine receptor agonists; NA, not applicable; TDF, Theoretical Domains Frameworks.

^a^ A total of 57 individuals were omitted by the software automatically due to missing data in some of the variables included in the model.

^b^ Significant result.

^c^ Scores at the TDF domain level were computed by averaging item scores for each domain.

**Table 4.** Factors Associated With Self-Reported Routine BZRA Deprescribing in Multivariable Logistic Regression for Hospital Physicians^a^

TDF domain^b^	OR (95% CI)	*P* value
Memory, attention, and decision processes	2.79 (1.73-4.69)^c^	<.001^c^
Goals	1.56 (0.89-2.75)	.12
Intentions	4.42 (2.38-8.83)^c^	<.001^c^
Emotions	1.75 (1.04-3.04)^c^	.04^c^

Abbreviations: BZRA, benzodiazepine receptor agonists; OR, odds ratio; TDF, Theoretical Domains Frameworks.

^a^ A total of 57 individuals were omitted by the software automatically due to missing data in some of the variables included in the model.

^b^ Scores at the TDF domain level were computed by averaging item scores for each domain.

^c^ Significant result.

## Discussion

In this cross-sectional, multinational, theory-based survey, we identified numerous physician barriers to BZRA deprescribing in older adults with sleep problems, across various TDF domains. Many were common across countries and for both hospital physicians and GPs. For hospital physicians, some of these barriers were associated with a lower intention to deprescribe and a lower rate of self-reported deprescribing.

Our findings align with previous studies—mainly qualitative and conducted in primary care settings—on general barriers to deprescribing and specific barriers to BZRA deprescribing, which identified barriers across most TDF domains.^22^ However, data on barriers from the hospital physicians’ perspectives are scarce. Keller et al^31^ interviewed 14 clinicians in a US tertiary hospital and identified similar barriers to the results of our study. They included a lack of knowledge about how to engage in deprescribing conversations, competing tasks in the inpatient setting, patient resistance, and lack of postdischarge follow-up. The latter aligns with extensive research highlighting the risks associated with care transitions in older adults.^32,33^

### Main Barriers and Implications

It is reassuring that most physicians know the risks of BZRA and believe that the benefits of deprescribing outweigh the risks. However, their knowledge and skills could be improved in several aspects of the deprescribing process, such as tapering and shared decision-making. This would strengthen self-efficacy (also referred to as beliefs about capabilities), which was associated with higher intentions to deprescribe in our sample.

Unsurprisingly, environmental and resource constraints were significant barriers. While increasing staff and resources may not be feasible, certain approaches could still facilitate deprescribing: (1) providing guidelines and tools to support physicians—for example, whiteboard videos have been shown to increase physicians’ self-efficacy^34^; (2) supporting interprofessional collaboration, such as involving pharmacists, was successful in many deprescribing interventions^16,35,36^; (3) improving access to cognitive behavioral therapy for insomnia (CBT-I), which is the first choice treatment for insomnia and can support BZRA deprescribing.^15,16^ Access remains limited across Europe, but self-help CBT-I can help address this issue.^37^

Two interesting findings emerge from the data on patients’ influence. First, our study, in line with previous research, shows that physicians feel pressured by patients to continue their BZRA and believe that most patients are reluctant to stop their medication.^31,38,39^ This contrasts with data showing that most patients are willing to consider deprescribing if recommended by their physician.^34^ Discrepancies between physicians’ perceptions and actual patient expectations are well-documented in deprescribing and, more broadly, low-value care.^40,41,42^ Upskilling physicians to efficiently ascertain and manage patient expectations seems worthwhile. Second, perceived patient influence was not associated with hospital physicians’ intentions or actions. This suggests that, while physicians may feel pressured by patients, it may not prevent them from deprescribing. This has not been explored for deprescribing, and it contrasts with evidence that perceived patient expectations may affect prescribing decisions.^40,43,44^ Further research is needed to explore these associations.

### Reflective and Impulsive Processes Associated With Intention and Behavior

Several TDF domains were associated with intention and/or self-reported behavior, and our data support a dual-process model interpretation of the findings. Dual process models propose that behavior results from the interplay of reflective and impulsive processes operating in parallel.^45,46,47^ Reflective processes involve conscious reflection and active decision-making based on perceived utility, outcomes, risk, capability, social influence, and intention. For example, we found that beliefs about consequences, beliefs about capabilities, and social and professional role and identity were associated with the intention to deprescribe. Intention was also unsurprisingly associated with behavior. In contrast, the impulsive process operates quickly and automatically with little cognitive effort, often driven by external (environmental) or internal (eg, affective) reactions. Interestingly, we found that the 2 most impulsive TDF domains (emotions and memory, attention, and decision processes) were significantly associated with behavior. These results and the fact that these factors were moderate or major barriers suggest that deprescribing interventions must address both processes.^48^ Most deprescribing interventions have focused primarily on the reflective component. Our data call for intervention components that also address the automatic aspects of deprescribing.^49^

### Similarities and Differences Among Physicians and Countries

Barriers were mostly similar across the 6 countries and between hospital physicians and GPs, suggesting that core intervention components could apply to all groups. Unsurprisingly, geriatricians had higher intentions to deprescribe than internists—reducing medication harm is a cornerstone of senior care—and GPs rated their role higher than hospital physicians. In many countries, the GPs’ role in deprescribing is central, and most deprescribing interventions to date have been developed in the primary care setting. Also, our data show that GPs welcome deprescribing initiated by hospital physicians. A limited number of studies, including a 2022 large-scale trial conducted in Canada, have demonstrated that deprescribing initiated in the hospital setting increases discontinuation.^50,51^ Finally, interpreting differences between countries is challenging due to the heterogeneity among participating specialists across countries and the lack of detailed data on organizational and health care system factors. As part of BE-SAFE, a planned qualitative study on care trajectories will provide additional information to explore cross-country differences and to inform on country-specific adaptations for the intervention.

### Strengths and Limitations

To our knowledge, this study is the first multinational, theory-based investigation of barriers to BZRA deprescribing. Dedicating a research stage to theorizing and testing via survey provides a transparent foundation for identifying what to focus on and then selecting strategies that best align with addressing those barriers.^52,53,54^

This study has several limitations. First, while questionnaires allow for reaching a large sample of participants, they may not fully capture participants’ perspectives. Second, we were unable to reach the target sample of 120 GPs, mainly due to the difficulties in recruiting GPs in Norway. Third, the self-reported measure of deprescribing is likely overestimated due to social desirability bias, the likelihood that participating physicians were more interested in BZRA deprescribing, and the lack of a clear definition of routine deprescribing. Consequently, the results of the multivariable analysis should be cautiously interpreted. Lastly, the cut-offs that categorized results into enablers, moderate or major barriers were somewhat arbitrary. However, this categorization was helpful for data interpretation and prioritization for intervention development.

## Conclusions

Hospital physicians and GPs across 6 European countries recognize the benefits of BZRA deprescribing in older adults but face numerous barriers in many different domains. Several of these barriers are associated with intention and/or action. Our findings indicate that effective deprescribing efforts require approaches that address both reflective processes (eg, enhancing capability) and impulsive processes (eg, managing emotions).

## Notes

Supplement 1. **eTable 1.** Background Characteristics of Hospital Physicians, by Country **eTable 2.** Background Characteristics of General Practitioners, by Country **eTable 3.** Barriers to and Enablers of BZRA Deprescribing by TDF-Based Domains and Items for Hospital Physicians: Country-Specific Data **eAppendix 1.** Summary of Answers to Open Questions on Barriers of and Enablers to BZRA Deprescribing (Hospital Physicians) **eTable 4.** Barriers to and Enablers of BZRA Deprescribing by TDF-Based Domains and Items for General Practitioners: Country-Specific Data **eAppendix 2.** Summary of Answers to Open Questions on Barriers of and Enablers to BZRA Deprescribing (General Practitioners)

Supplement 2.
Data Sharing Statement
