# Supplementary material for: Barriers to Deprescribing Benzodiazepines in Older Adults in a Survey of European Physicians
Source: JAMA Netw Open. 2025 Mar 3;8(3):e2459883. doi: 10.1001/jamanetworkopen.2024.59883 (PMC11877185; doi:10.1001/jamanetworkopen.2024.59883)
Supplement: Supplement 1. — eTable 1. Background Characteristics of Hospital Physicians, by Country eTable 2. Background Characteristics of General Practitioners, by Country eTable 3. Barriers to and Enablers of BZRA Deprescribing by TDF-Based Domains and Items for Hospital Physicians: Country-Specific Data eAppendix 1. Summary of Answers to Open Questions on Barriers of and Enablers to BZRA Deprescribing (Hospital Physicians) eTable 4. Barriers to and Enablers of BZRA Deprescribing by TDF-Based Domains and Items for General Practitioners: Country-Specific Data eAppendix 2. Summary of Answers to Open Questions on Barriers of and Enablers to BZRA Deprescribing (General Practitioners) [file jamanetwopen-e2459883-s001.pdf]

Supplemental Online Content

Shapoval V, de Saint Hubert M, Evrard P, et al. Physicians' barriers to deprescribing benzodiazepines in older adults. *JAMA Netw Open.* 2025;8(2):e2459883. doi:10.1001/jamanetworkopen.2024.59883

eTable 1. Background Characteristics of Hospital Physicians, by Country

eTable 2. Background Characteristics of General Practitioners, by Country

eTable 3. Barriers to and Enablers of BZRA Deprescribing by TDF-Based Domains and Items for Hospital Physicians: Country-Specific Data

eAppendix 1. Summary of Answers to Open Questions on Barriers of and Enablers to BZRA Deprescribing (Hospital Physicians)

eTable 4. Barriers to and Enablers of BZRA Deprescribing by TDF-Based Domains and Items for General Practitioners: Country-Specific Data

eAppendix 2. Summary of Answers to Open Questions on Barriers of and Enablers to BZRA Deprescribing (General Practitioners)

This supplemental material has been provided by the authors to give readers additional information about their work.

© 2025 Shapoval V et al. *JAMA Network Open*.

eTable 1. Background characteristics of hospital physicians, by country

Characteristic	Belgium No. (%) (n=43) [a]	Greece No. (%) (n =40) [a]	Norway No. (%) (n =37) [a]	Poland No. (%) (n=40) [a]	Spain No. (%) (n=40) [a]	Switzerland No. (%) (n=40) [a]
**Age, y**						
≤ 30	18 (41.9)	10 (25)	8 (21.6)	12 (30)	3 (7.5)	14 (35)
31 – 40	12 (27.9)	3 (7.5)	11 (29.7)	7 (17.5)	8 (20)	22 (55)
41 – 50	7 (16.3)	16 (40)	12 (32.4)	16 (40)	10 (25)	3 (7.5)
51 – 60	5 (11.6)	8 (20)	4 (10.8)	4 (10)	10 (25)	0 (0)
≥ 61	1 (2.3)	3 (7.5)	2 (5.5)	1 (2.5)	9 (22.5)	1 (2.5)
**Gender**						
Male	17 (40.5)	20 (50)	17 (45.9)	14 (37.8)	12 (30)	11 (27.5)
Female	25 (59.5)	20 (50)	20 (54.1)	23 (62.2)	27 (67.5)	29 (72.5)
Prefer not to answer	0 (0)	0 (0)	0 (0)	0 (0)	1 (2.5)	0 (0)
**Occupation, hospital physicians**						
Geriatrician	10 (23.3)	1 (2.5)	18 (48.6)	0 (0)	15 (37.5)	0 (0)
Psychiatrist	4 (9.3)	1 (2.5)	4 (10.9)	0 (0)	1 (2.5)	0 (0)
Internist	8 (18.6)	22 (55)	15 (40.5)	0 (0)	13 (32.5)	40 (100)
Cardiologist	0 (0)	0 (0)	0 (0)	20 (50)	0 (0)	0 (0)
Neurologist	6 (14)	0 (0)	0 (0)	20 (50)	2 (5)	0 (0)
Other	15 (34.9)	16 (40)	0 (0)	0 (0)	9 (22.5)	0 (0)
**Type of care, hospital physicians**						
Inpatients	17 (39.5)	13 (32.5)	17 (45.9)	19 (48.7)	12 (30.8)	33 (82.5)
Outpatients (clinics)	5 (11.7)	3 (7.5)	3 (8.2)	4 (10.3)	11 (28.2)	1 (2.5)
Both (inpatients/outpatients)	21 (48.8)	18 (45)	17 (45.9)	11 (41)	16 (41)	6 (15)
**Experience, y**						
< 5	16 (37.2)	8 (20)	11 (29.7)	14 (35)	4 (10)	23 (57.5)
5 – 9	10 (23.3)	7 (17.5)	3 (8.2)	4 (10)	7 (17.5)	11 (27.5)
10 – 14	5 (11.6)	8 (20)	9 (24.3)	3 (7.5)	1 (2.5)	3 (7.5)
15 – 19	3 (7)	4 (10)	7 (18.9)	7 (17.5)	8 (20)	1 (2.5)
≥ 20	9 (20.9)	13 (32.5)	7 (18.9)	12 (30)	20 (50)	2 (5)
**Deprescribed BZRA before, self-reported**						
Yes	34 (79.1)	22 (56.4)	35 (94.6)	26 (66.6)	34 (87.2)	33 (84.6)
No	9 (20.9)	17 (43.6)	2 (5.4)	13 (33.4)	5 (12.8)	6 (15.4)
**Deprescribe BZRA routinely, self-reported**						
Yes	32 (74.4)	19 (48.8)	18 (48.6)	18 (46.2)	22 (56.4)	26 (66.6)
No	11 (25.6)	20 (51.2)	19 (51.4)	21 (53.8)	17 (43.6)	13 (33.4)

[a] Missing response (excluded from percentages) totaled 4 for Gender (1 in Belgium; 3 in Poland), 2 for Type of care (1 in Poland; 1 in Spain), 4 for Deprescribed BZRA before (1 in Greece; 1 in Poland; 1 in Spain; 1 in Switzerland), 4 for Deprescribe BZRA routinely (1 in Greece; 1 in Poland; 1 in Spain; 1 in Switzerland

© 2025 Shapoval V et al. *JAMA Network Open*.

eTable 2. Background characteristics of general practitioners, by country

Characteristic	Belgium No. (%) (n=20) [a]	Greece No. (%) (n=19) [a]	Norway No. (%) (n=2) [a]	Poland No. (%) (n=19) [a]	Spain No. (%) (n=16) [a]	Switzerland No. (%) (n=20) [a]
**Age, y**						
less than 30 years old	1 (5.0)	0 (0)	0 (0)	3 (15.8)	1 (6.2)	0 (0)
31 – 40 years old	4 (20.0)	6 (31.6)	0 (0)	8 (42.1)	4 (25.0)	6 (30.0)
41 – 50 years old	5 (25.0)	6 (31.6)	1 (50.0)	3 (15.8)	5 (31.2)	8 (40.0)
51 – 60 years old	4 (20.0)	7 (36.8)	1 (50.0)	5 (26.3)	6 (37.5)	4 (20.0)
61 years old and more	6 (30.0)	0 (0)	0 (0)	0 (0)	0 (0)	2 (10.0)
**Gender**						
Men	13 (65.0)	13 (68.4)	1 (50.0)	4 (21.1)	5 (31.2)	8 (40.0)
Women	7 (35)	6 (31.6)	1 (50.0)	15 (78.9)	11 (68.8)	12 (60.0)
**Type of practice, general practitioner**						
Solo practice (in the office on my own)	5 (25.0)	8 (42.1)	0 (0)	4 (21.1)	2 (14.3)	2 (10.0)
Group practice, monodisciplinary	8 (40.0)	0 (0)	1 (50.0)	0 (0)	2 (14.3)	9 (45.0)
Group practice, multidisciplinary: PS and non-physicians)	6 (30.0)	2 (10.5)	0 (0)	6 (31.6)	5 (35.7)	4 (20.0)
Group practice, multidisciplinary: GPs, specialists, and non-physicians	1 (5.0)	9 (47.4)	1 (50.0)	9 (47.4)	5 (35.7)	5 (25.0)
**Experience, y**						
Less than 5 years	1 (5.0)	4 (21.1)	0 (0)	4 (21.1)	2 (12.5)	0 (0)
5 – 9 years	3 (15.0)	5 (26.3)	0 (0)	8 (42.1)	3 (18.8)	4 (20.0)
10 – 14 years	2 (10.0)	2 (10.5)	0 (0)	3 (15.8)	2 (12.5)	4 (20.0)
15 – 19 years	2 (10.0)	3 (15.8)	1 (50.0)	0 (0)	2 (12.5)	6 (30.0)
20 years or more	12 (60.0	5 (26.3)	1 (50.0)	4 (21.1)	7 (43.8)	6 (30.0)
**Deprescribed BZRA before, self-reported**						
Yes	18 (100)	13 (68.4)	2 (100)	14 (87.5)	15 (93.8)	20 (100)
No	0 (0)	6 (31.6)	0 (0)	2 (12.5)	1 (6.2)	0 (0)
**Deprescribed BZRA routinely, self-reported**						
Yes	15 (83.3)	13 (68.4)	2 (100)	9 (56.3)	10 (62.5)	17 (85.0)
No	3 (16.7)	6 (31.6)	0 (0)	7 (43.7)	6 (37.5)	3 (15.0)

[a] Missing response (excluded from percentages) totaled 2 for Type of practice (2 in Spain), 5 for Deprescribed BZRA before (2 in Belgium, 3 in Poland), 5 for Deprescribed BZRA routinely (2 in Belgium, 3 in Poland

© 2025 Shapoval V et al. *JAMA Network Open*.

eTable 3. Barriers to and enablers of BZRA deprescribing by TDF-based domains and items for hospital physicians: country-specific data.

The colour code for the tables below		
Enabler (mean ≥ 4.00)	Moderate Barrier (mean 3.00-3.99)	Major Barrier (mean ≤ 2.99)

eTable 3.1. Knowledge

TDF-based items	Mean (SD)					
	Belgium	Greece	Norway	Poland	Spain	Switzerland
I know the risks associated with the use of BZRA in older adults with sleep problems.	4.28 (0.93)	4.47 (0.72)	4.46 (0.69)	4.03 (0.90)	4.33 (0.70)	4.38 (0.49)
I know the situations or comorbidities in which BZRA deprescribing is not recommended.	3.07 (1.08)	3.88 (1.04)	3.46 (0.93)	3.05 (0.98)	3.79 (0.96)	3.13 (1.04)
I know how to taper BZRA in older adults with sleep problems.	3.28 (1.08)	3.67 (1.02)	3.35 (0.95)	3.03 (1.18)	3.90 (1.02)	2.74 (1.02)
I know how to engage patients about deprescribing BZRA.	3.12 (0.93)	3.60 (1.10)	3.39 (0.84)	2.90 (0.94)	3.73 (0.82)	3.55 (0.88)
I am aware of alternative approaches to deal with sleep problems in older adults.	3.70 (1.04)	3.75 (1.17)	3.59 (0.76)	3.62 (0.94)	3.85 (0.92)	3.92 (0.62)

I know the risks associated with the use of BZRA in older adults with sleep problems.

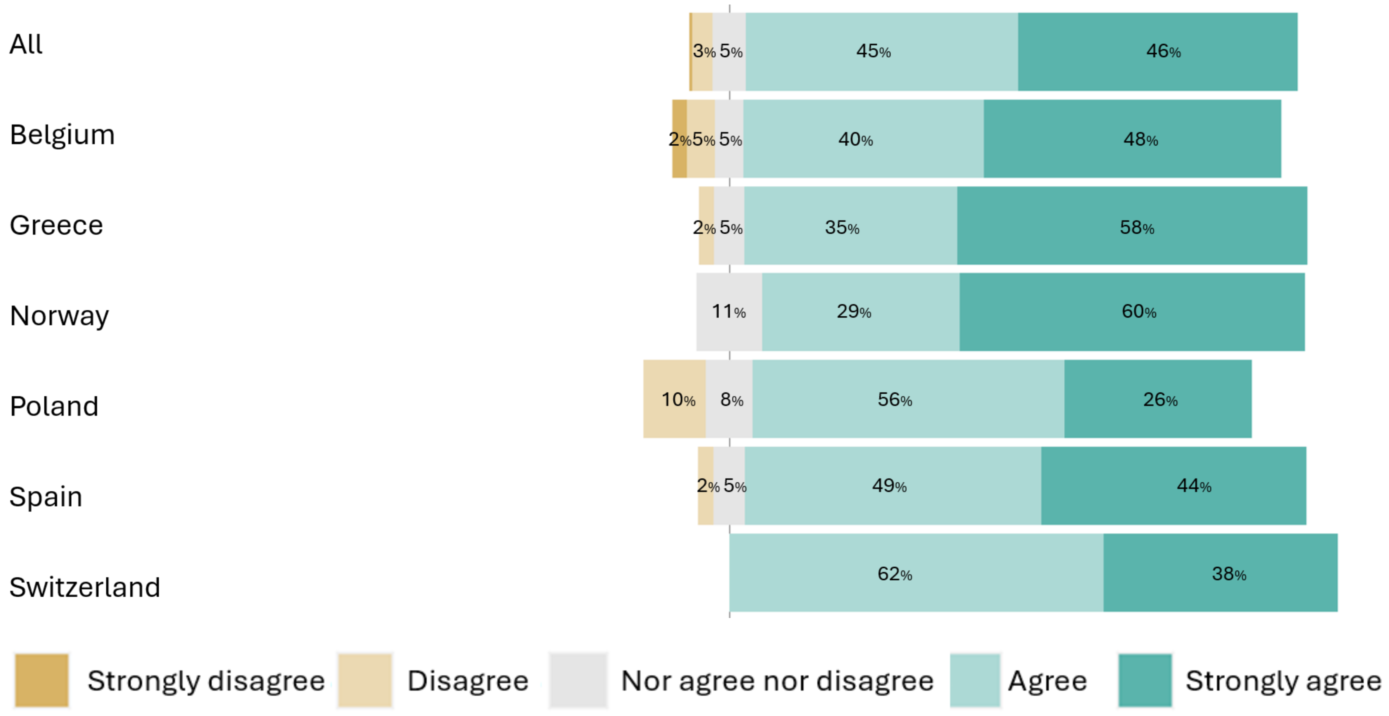

© 2025 Shapoval V et al. *JAMA Network Open*.

I know the situations or comorbidities in which BZRA deprescribing is not recommended.

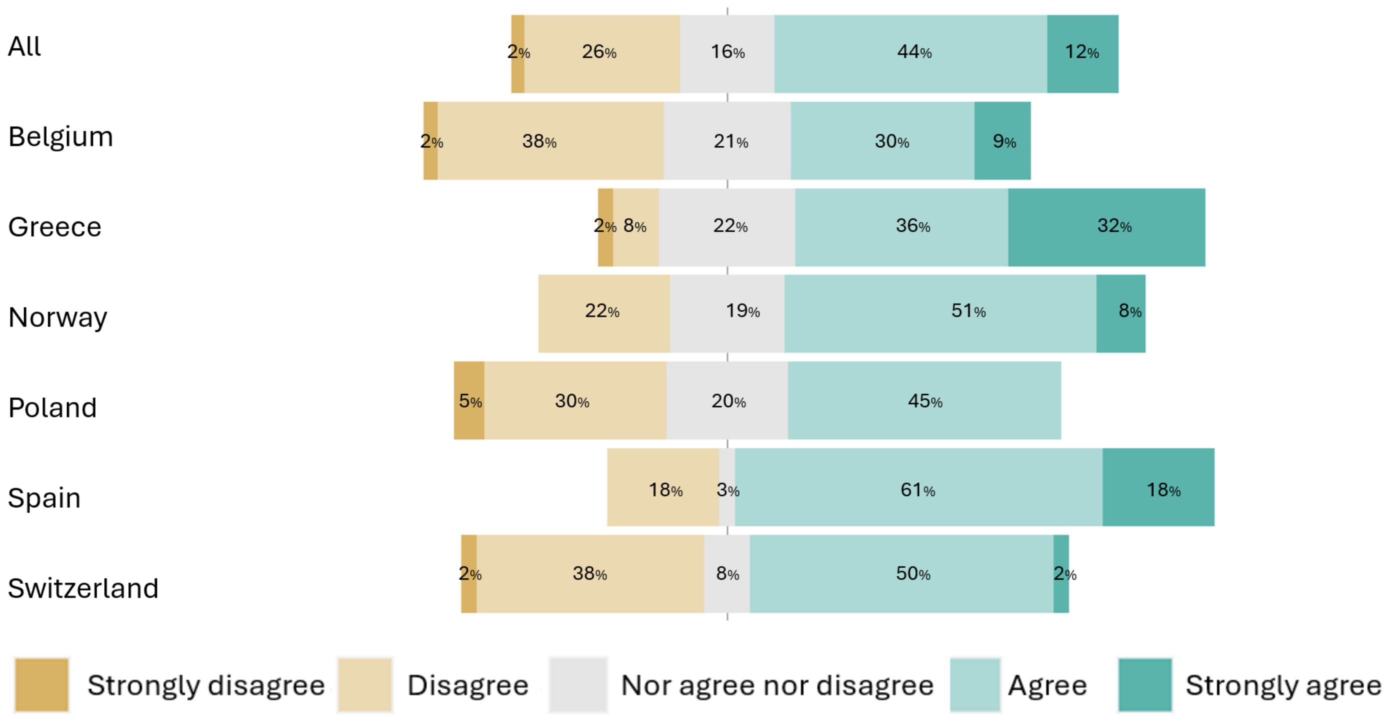

I know how to taper BZRA in older adults with sleep problems.

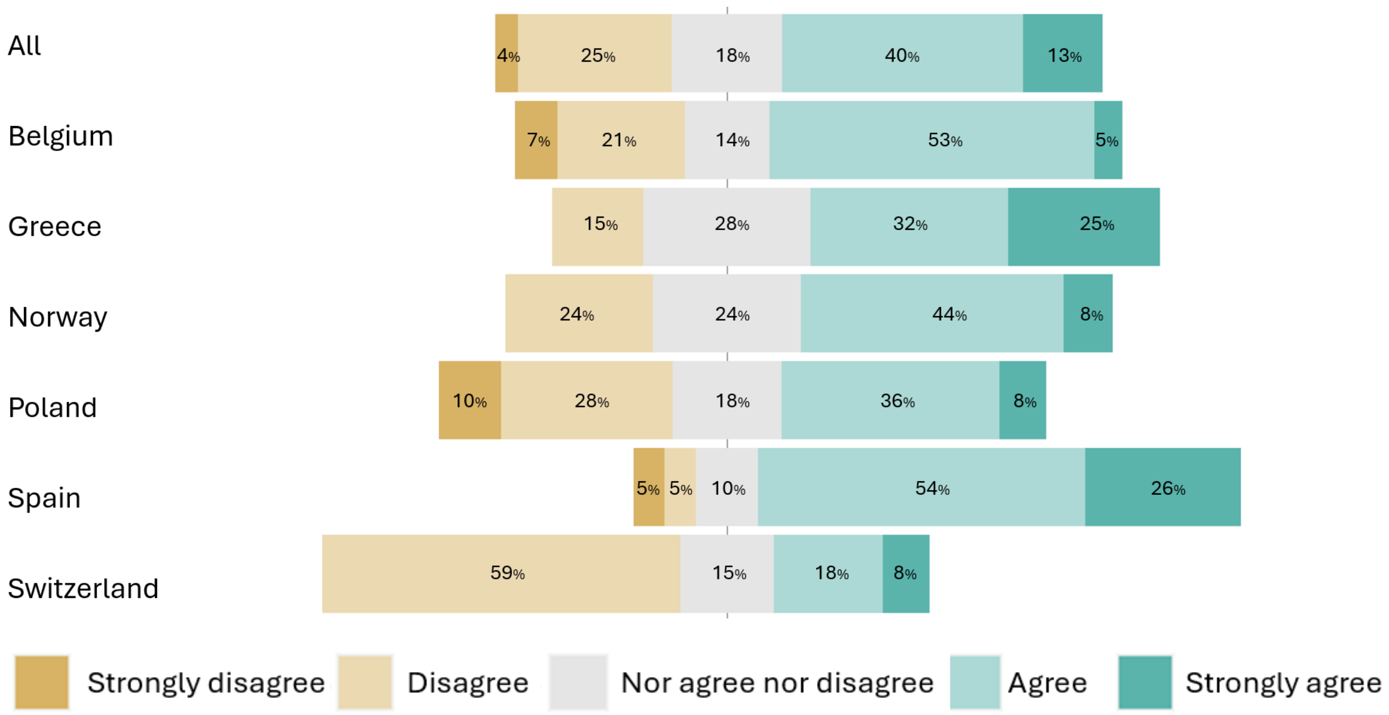

© 2025 Shapoval V et al. *JAMA Network Open*.

I know how to engage patients about deprescribing BZRA.

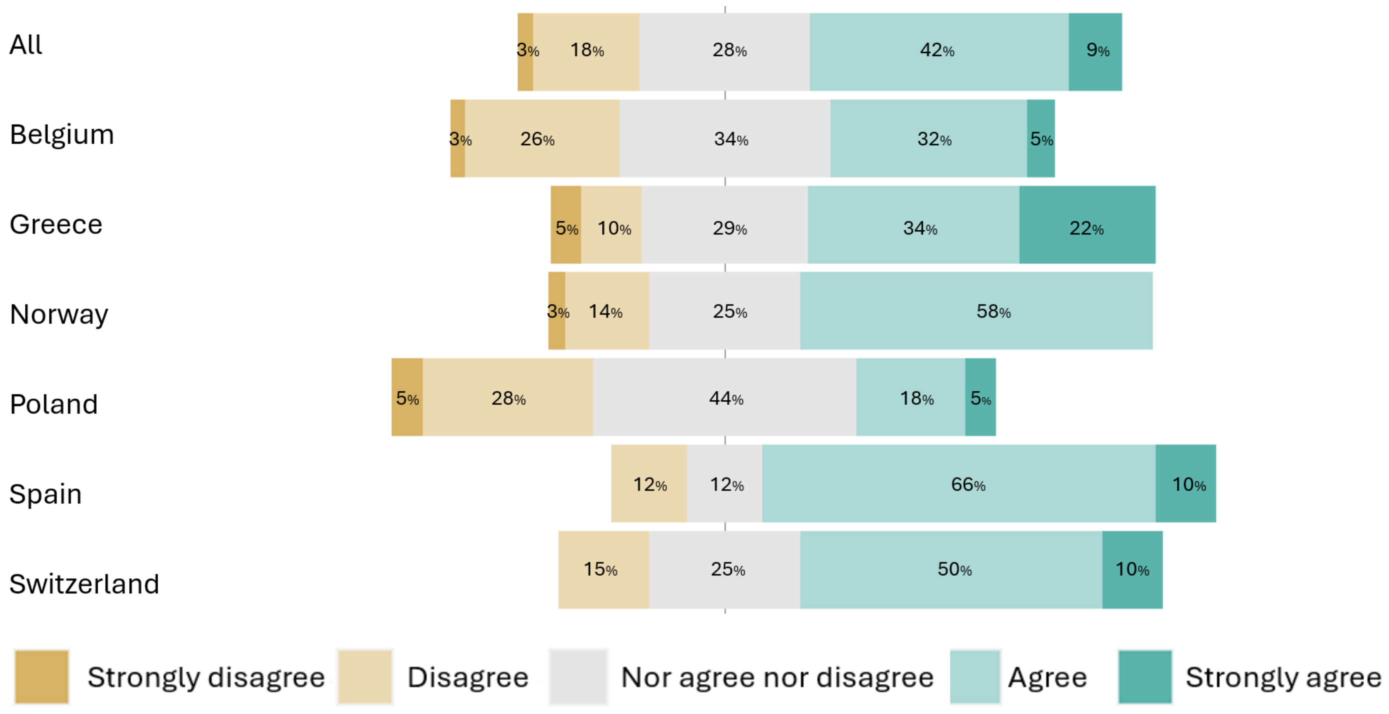

I am aware of alternative approaches to deal with sleep problems in older adults.

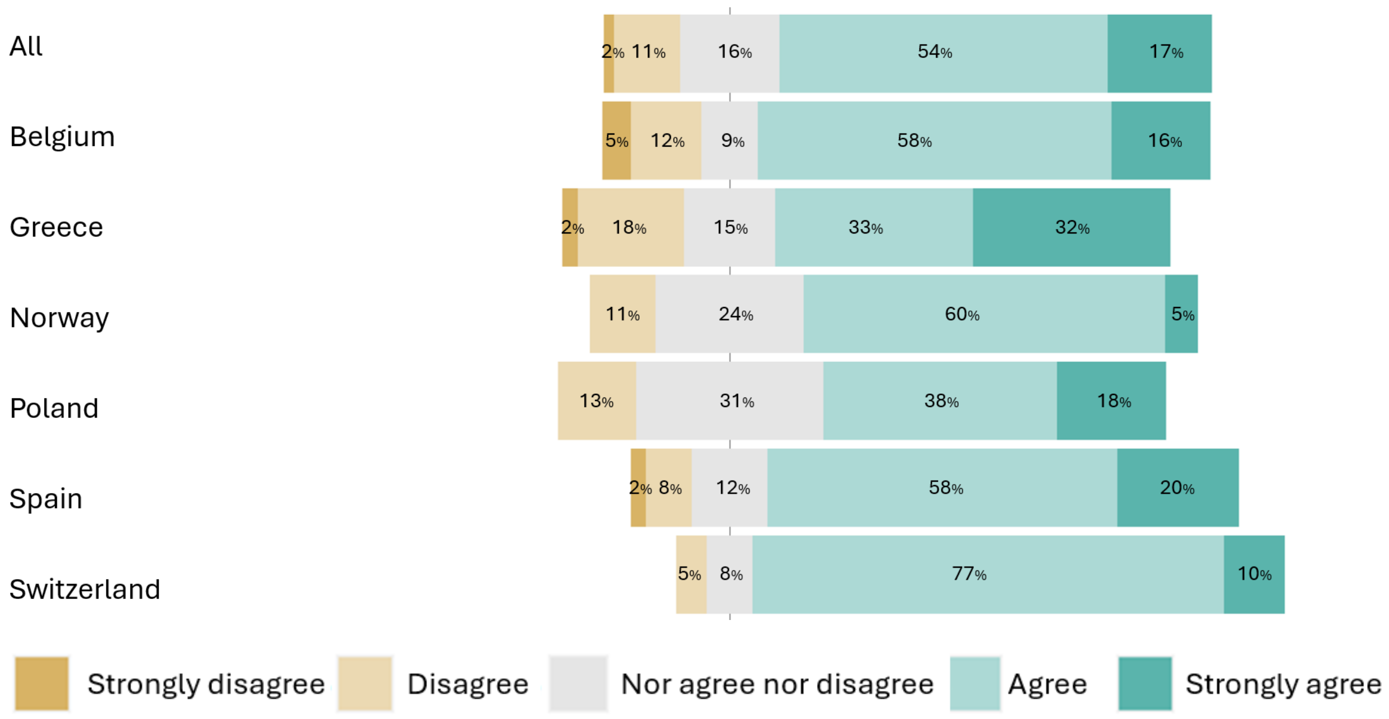

© 2025 Shapoval V et al. *JAMA Network Open*.

eTable 3.2. Skills

TDF-based items	Mean (SD)					
	Belgium	Greece	Norway	Poland	Spain	Switzerland
I have been trained on how to deprescribe BZRA in older adults with sleep problems.	2.70 (1.30)	3.05 (1.36)	2.81 (1.02)	2.41 (1.31)	2.95 (1.17)	2.51 (0.99)
I have been trained to engage patients about BZRA deprescribing.	2.33 (1.32)	3.05 (1.15)	2.51 (0.90)	2.45 (1.32)	2.79 (1.19)	2.92 (1.09)
I have been trained to implement alternative approaches for sleep problems in older adults.	2.67 (1.21)	2.95 (1.38)	2.78 (1.11)	2.52 (1.18)	3.00 (1.27)	3.00 (1.08)

I have been trained on how to deprescribe BZRA in older adults with sleep problems.

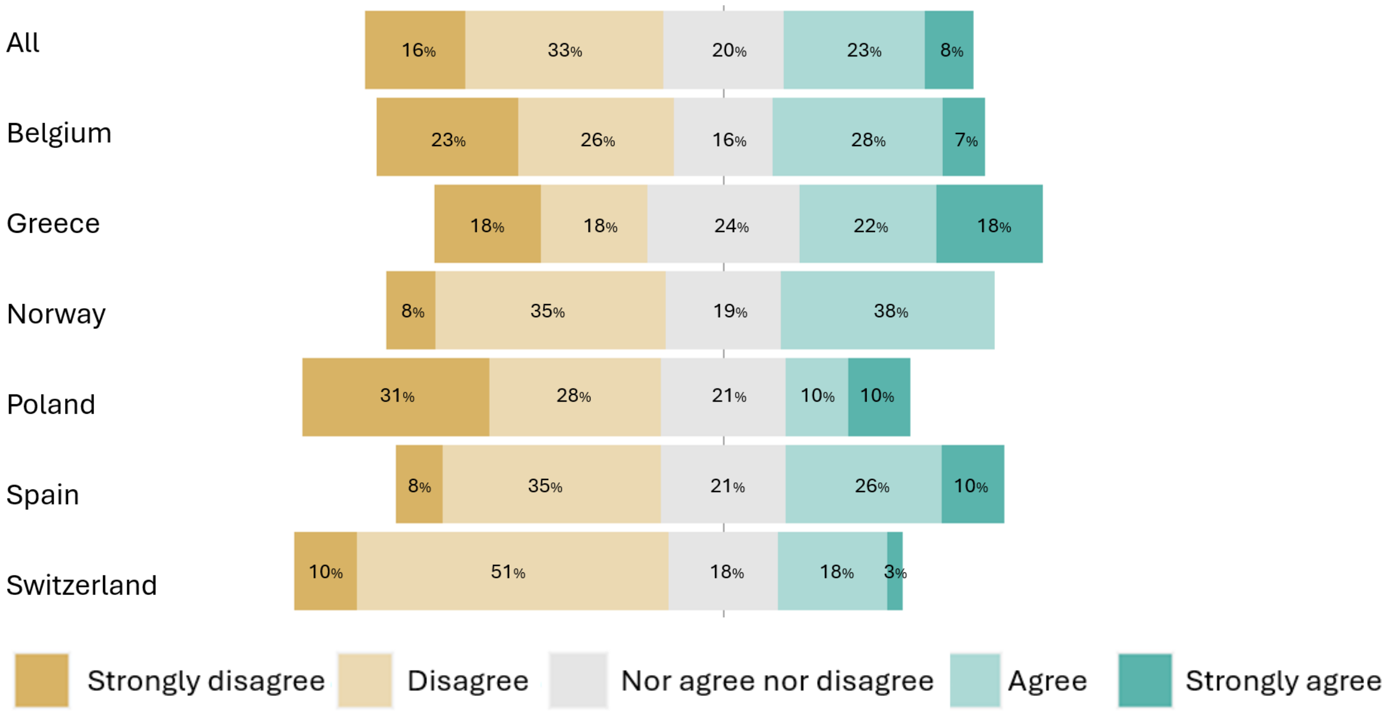

© 2025 Shapoval V et al. *JAMA Network Open*.

I have been trained to engage patients about BZRA deprescribing.

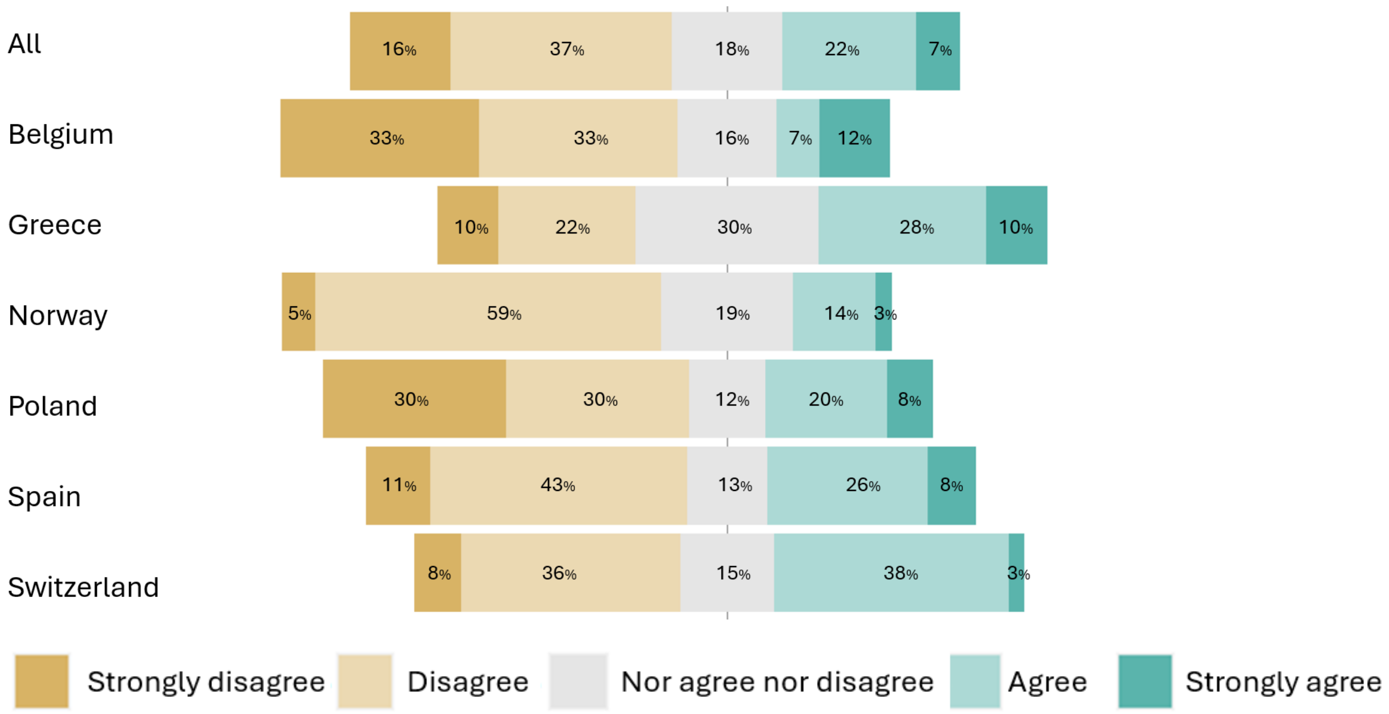

I have been trained to implement alternative approaches for sleep problems in older adults.

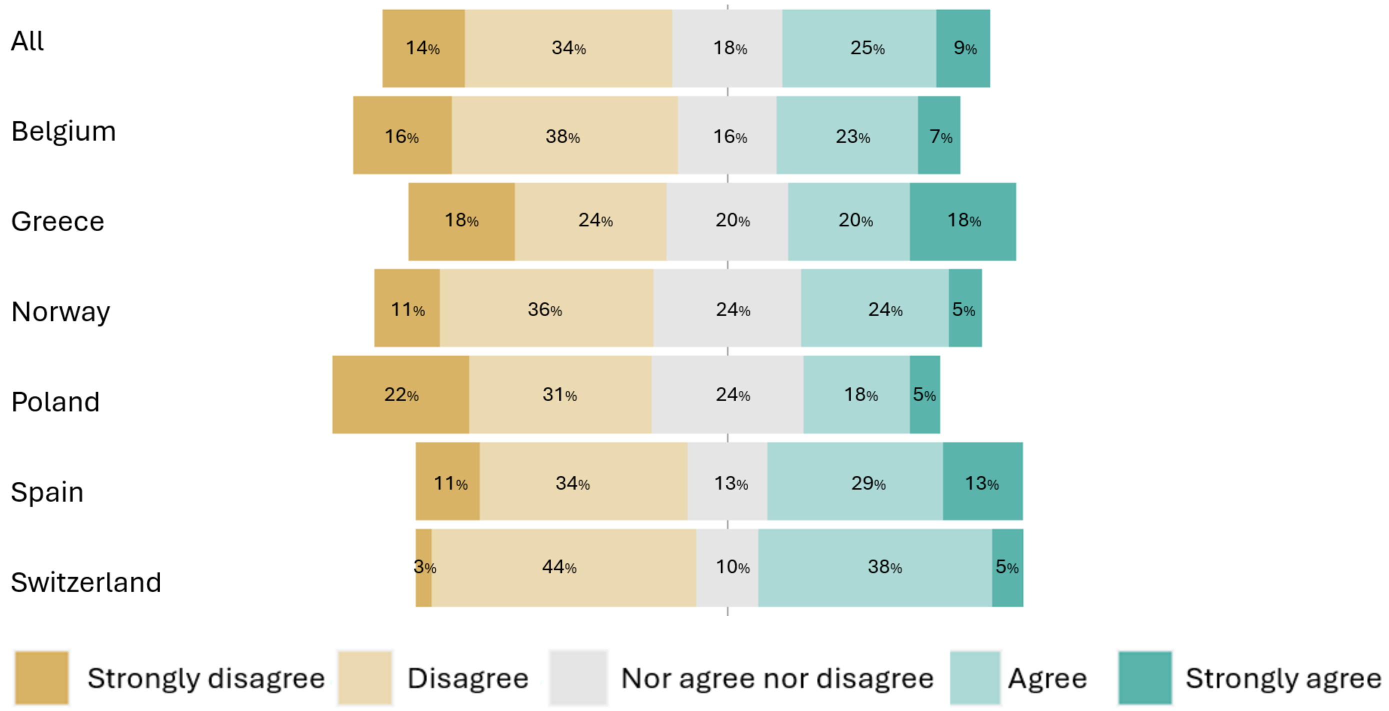

© 2025 Shapoval V et al. *JAMA Network Open*.

eTable 3.3. Memory, attention, and decision processes

TDF-based items	Mean (SD)					
	Belgium	Greece	Norway	Poland	Spain	Switzerland
I usually do not consider deprescribing BZRA in older adults with sleep problems because it is a difficult and time-consuming process.	3.55* (1.02)	3.13* (1.09)	2.89* (0.91)	3.05* (1.20)	3.55* (0.89)	3.31* (0.86)
As long as the patient has no specific issue or request, I renew/continue the prescription of BZRA.	3.31* (1.07)	3.25* (1.26)	3.27* (0.90)	3.58* (0.86)	3.08* (1.01)	3.72* (0.72)

* reversed mean applied to negative statements (so that for all items, the lower the mean, the higher the barrier)

I usually do not consider deprescribing BZRA in older adults as it is a difficult and time-consuming process.

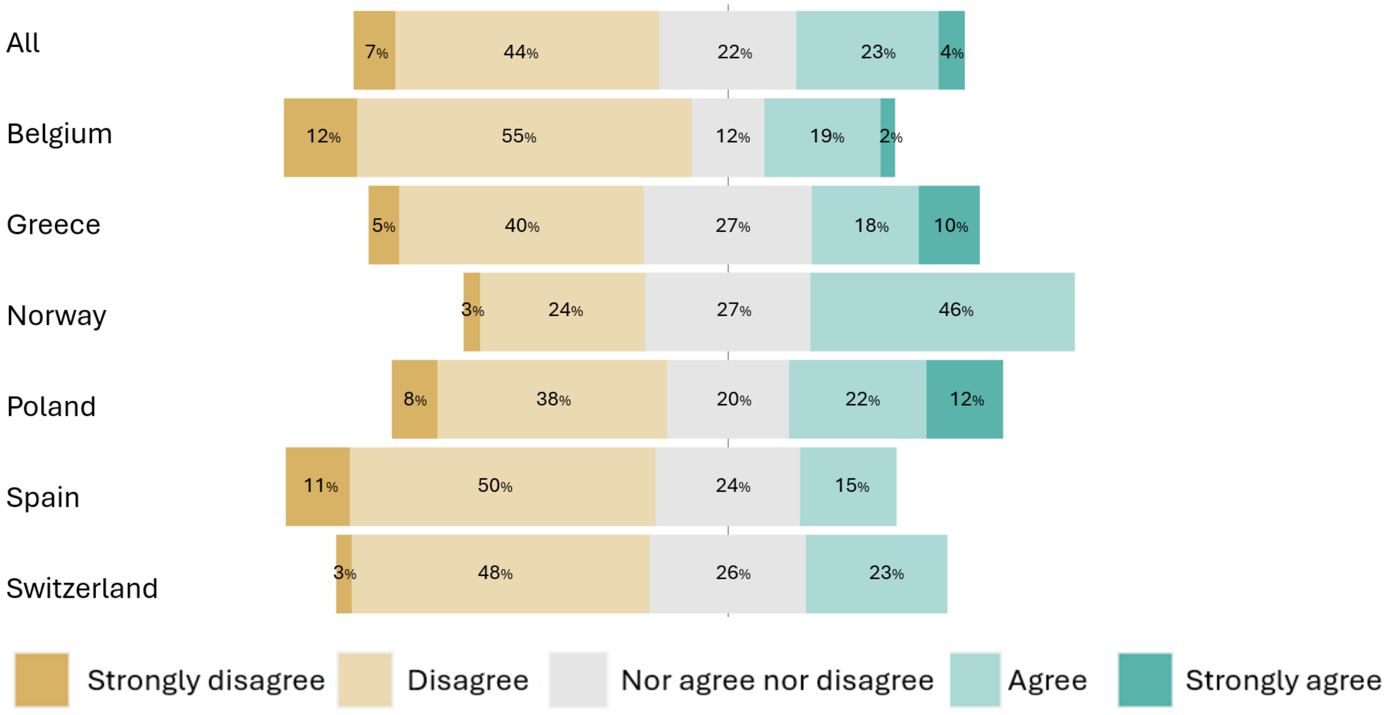

© 2025 Shapoval V et al. *JAMA Network Open*.

As long as the patient has no specific issue or request, I renew/continue the prescription of BZRA.

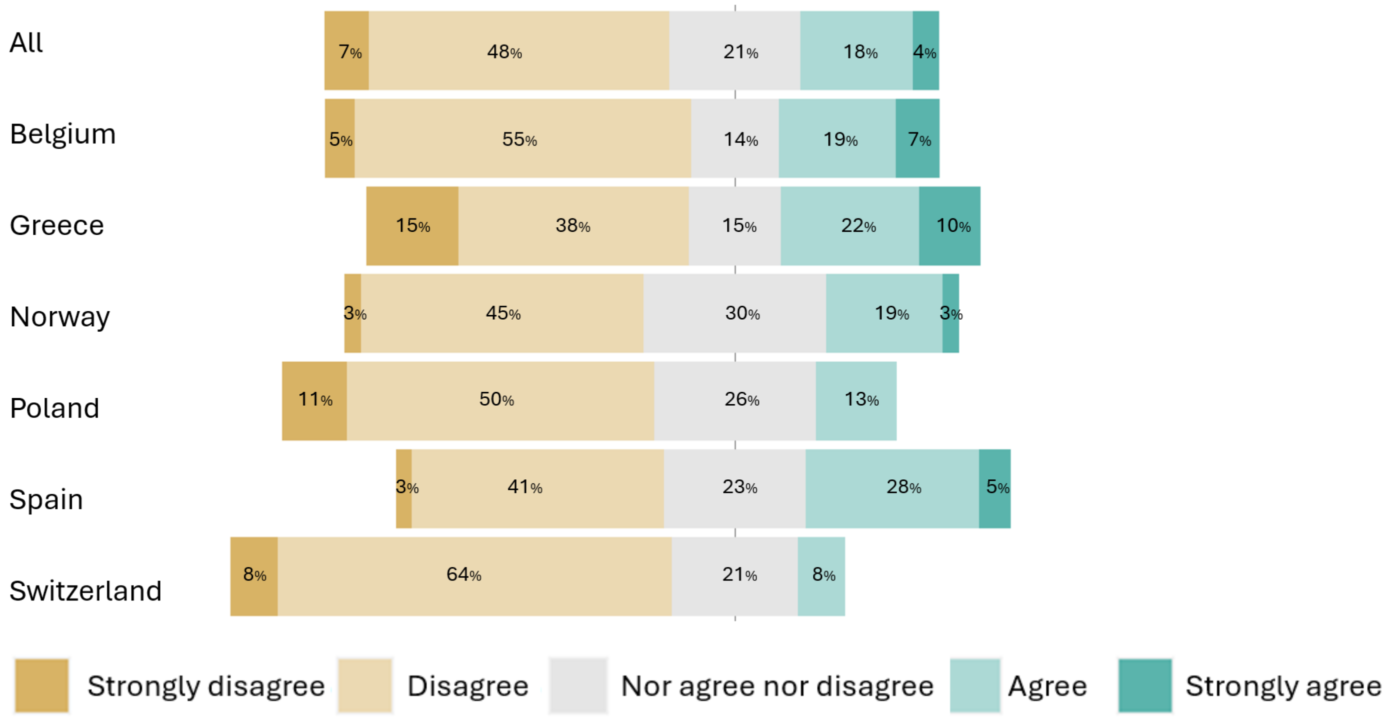

eTable 3.4. Social/Professional Role and Identity

TDF-based items	Mean (SD)					
	**Belgium**	**Greece**	**Norway**	**Poland**	**Spain**	**Switzerland**
It is my responsibility as a physician to deprescribe BZRA in older adults with sleep problems.	4.23 (0.68)	3.36 (1.20)	3.81 (0.78)	3.40 (0.96)	3.90 (1.08)	3.85 (0.70)
In the department or institution where I work, it is relevant to initiate BZRA deprescribing.	4.19 (0.86)	3.15 (0.89)	3.86 (0.71)	2.63 (1.00)	3.45 (1.13)	3.61 (0.97)
I do not feel concerned with BZRA deprescribing in older adults.	4.30* (0.91)	3.28* (1.07)	3.24* (0.89)	3.33* (0.93)	4.30* (0.85)	3.67* (0.77)

*reversed mean applied to negative statements (so that for all items, the lower the mean, the higher the barrier)

© 2025 Shapoval V et al. *JAMA Network Open*.

It is my responsibility as a physician to deprescribe BZRA in older adults with sleep problems.

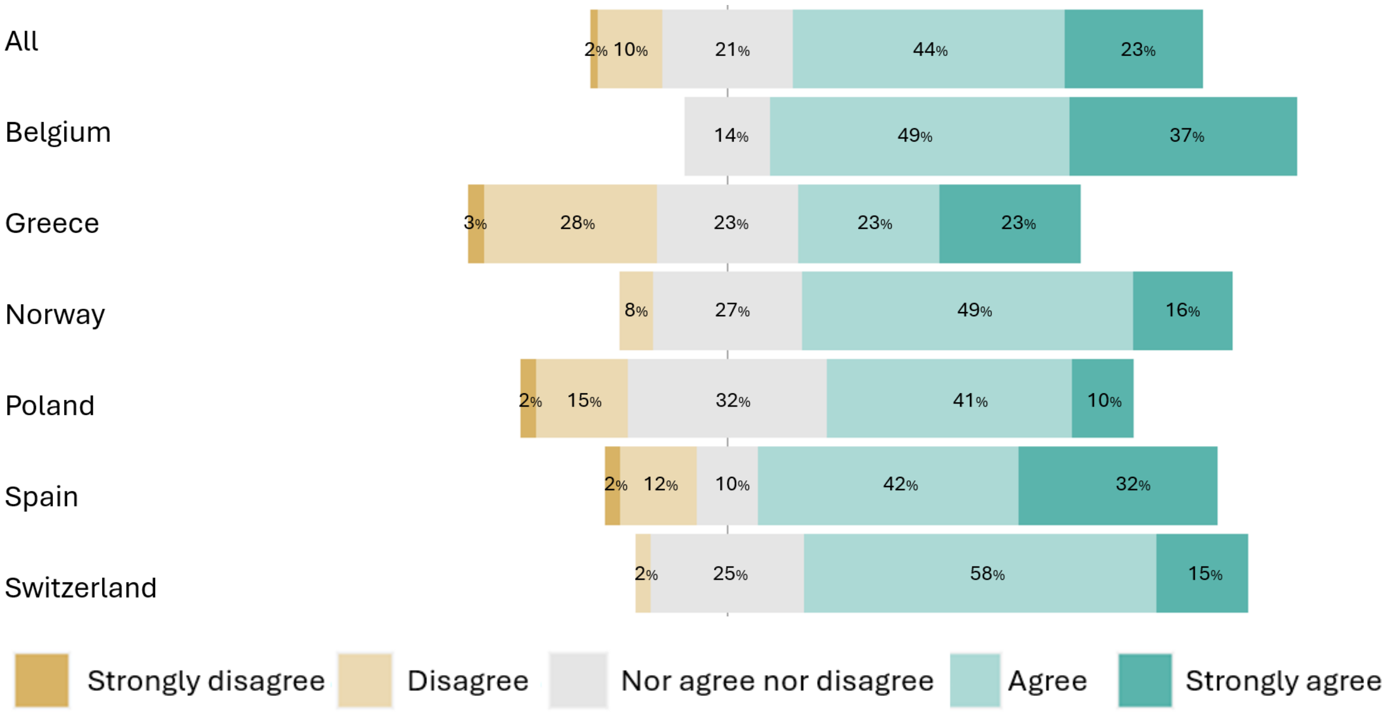

In the department or institution where I work, it is relevant to initiate BZRA deprescribing.

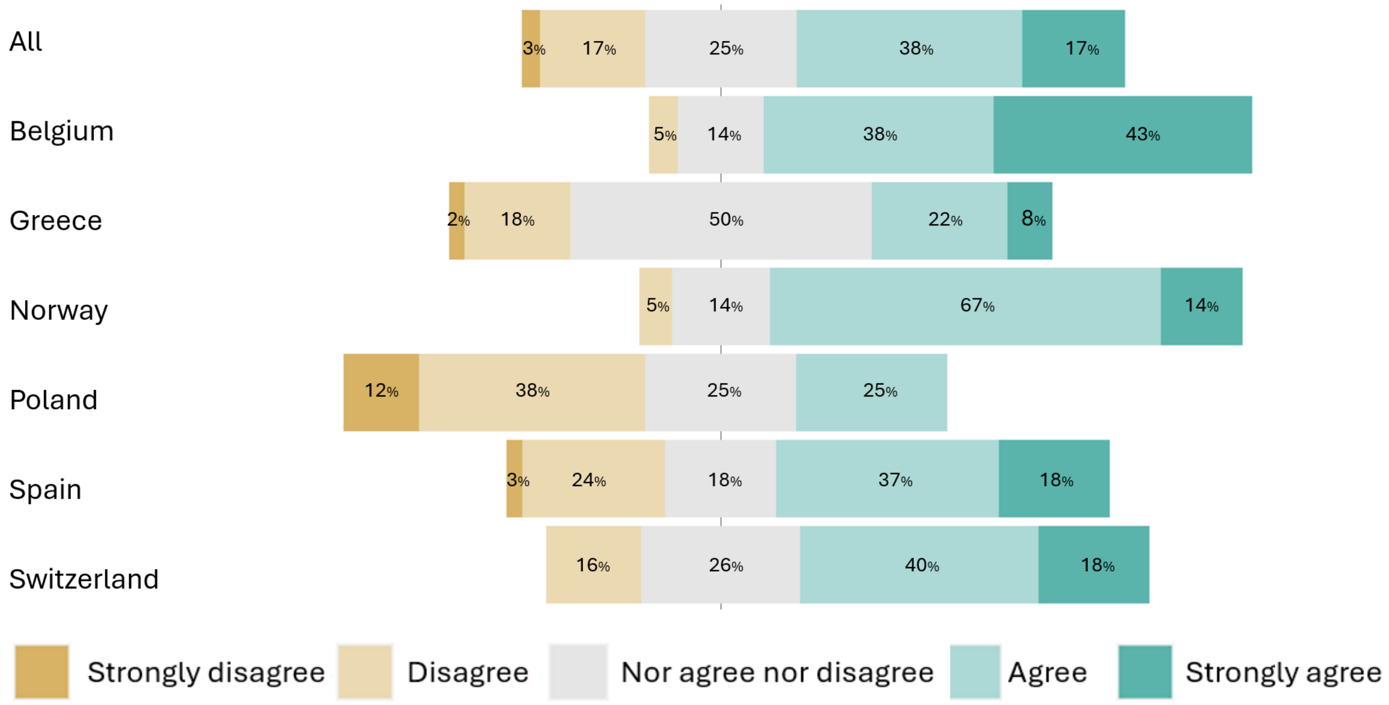

© 2025 Shapoval V et al. *JAMA Network Open*.

I do not feel concerned with BZRA deprescribing in older adults.

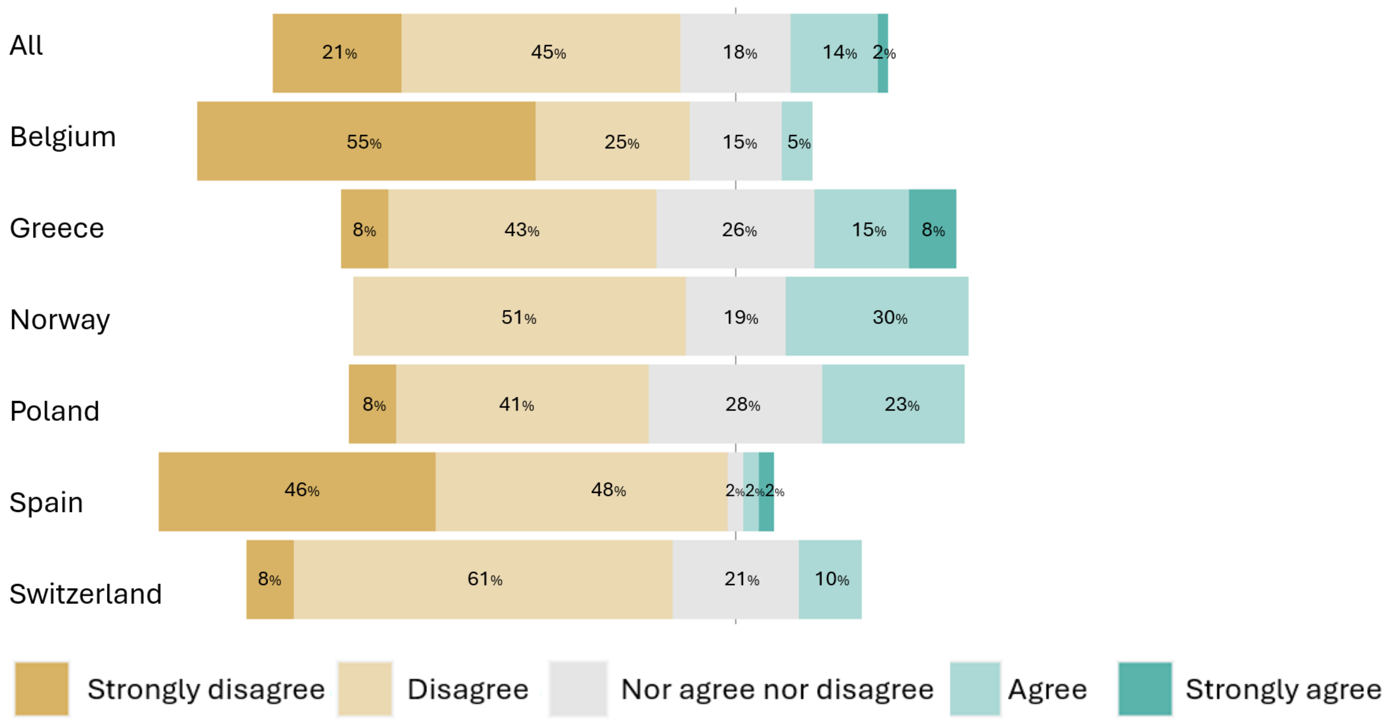

eTable 3.5. Beliefs about capabilities

TDF-based items	Mean (SD)					
	Belgium	Greece	Norway	Poland	Spain	Switzerland
I am confident that I can deprescribe BZRA in older adults with sleep problems even when I have limited time.	2.63 (1.02)	2.77 (1.10)	2.62 (0.86)	2.47 (0.95)	3.82 (0.721)	2.65 (0.95)
I am confident I could deprescribe BZRA in older adults with sleep problems if I wanted to.	3.47 (1.12)	2.92 (1.12)	3.19 (0.82)	3.13 (0.96)	3.82 (0.79)	3.25 (0.87)

© 2025 Shapoval V et al. *JAMA Network Open*.

I am confident, I can deprescribe BZRA in older adults with sleep problems even when I have limited time.

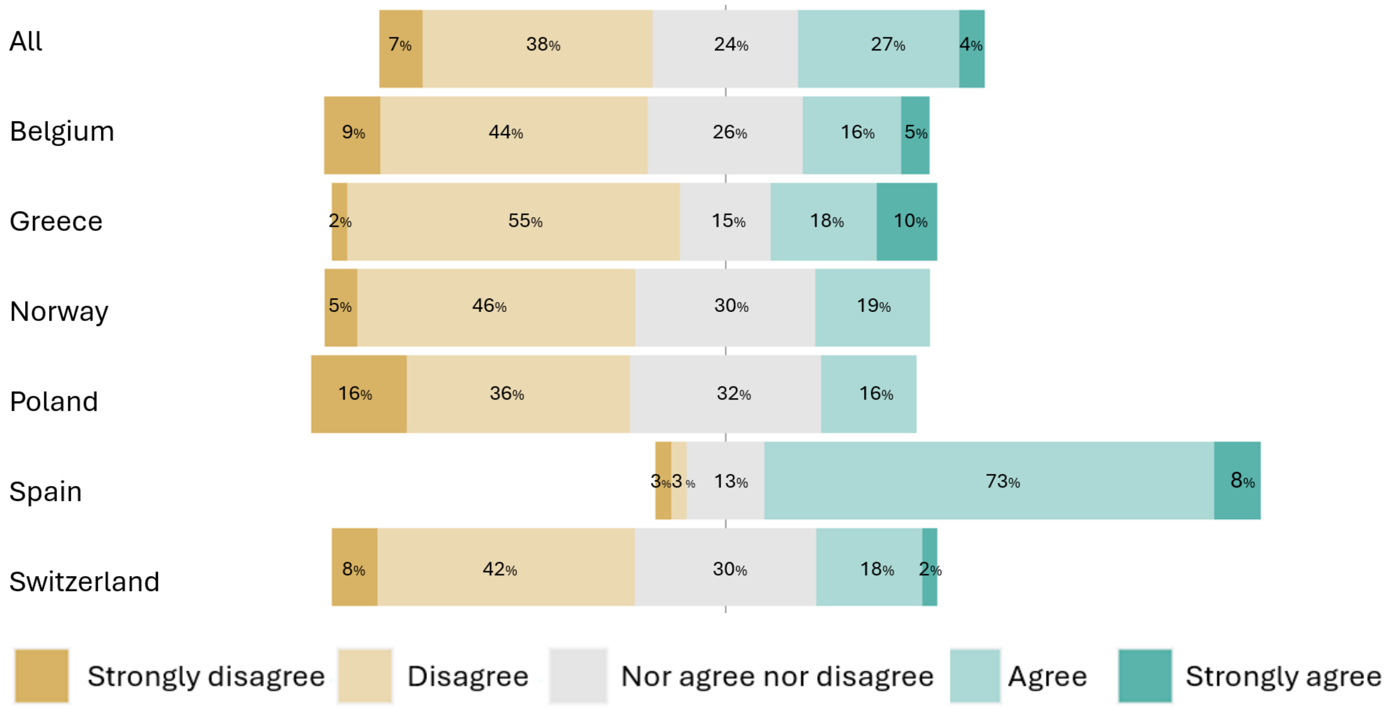

I am confident I could deprescribe BZRA in older adults with sleep problems if I wanted to.

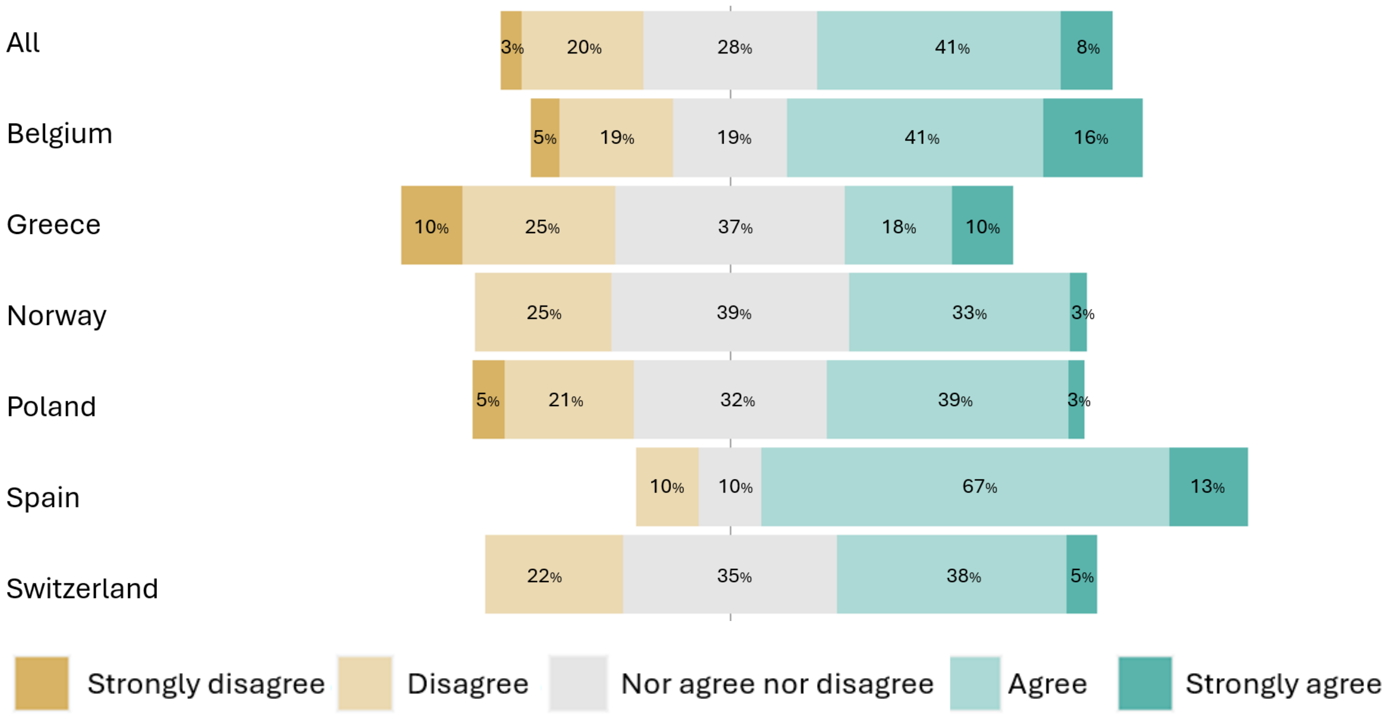

© 2025 Shapoval V et al. *JAMA Network Open*.

eTable 3.6. Beliefs about consequences

TDF-based items	Mean (SD)					
	Belgium	Greece	Norway	Poland	Spain	Switzerland
If I deprescribe BZRA to older adults with sleep problems, it will benefit the population's health in general.	4.35 (0.69)	3.45 (0.99)	4.05 (0.66)	3.56 (0.68)	3.79 (0.89)	4.03 (0.81)
If I deprescribe BZRA in older adults with sleep problems, it will negatively affect my relationship with these patients.	3.90* (0.75)	3.48* (0.93)	2.97* (0.89)	3.40* (0.81)	3.89* (0.79)	3.27* (0.78)
I believe that BZRA deprescribing in older adults with sleep problems will have negative consequences for my patient's health.	3.60* (0.85)	3.10* (0.90)	3.49* (0.77)	3.30* (0.85)	3.44* (0.99)	3.49* (0.89)
In general, I believe that the benefits of deprescribing BZRA in older adults with sleep problems outweigh the harms.	4.30 (0.89)	3.49 (0.89)	4.16 (0.60)	3.70 (0.94)	4.18 (0.91)	4.13 (0.73)

*reversed mean applied to negative statements (so that for all items, the lower the mean, the higher the barrier)

If I deprescribe BZRA to older adults with sleep problems, it will benefit the population's health in general.

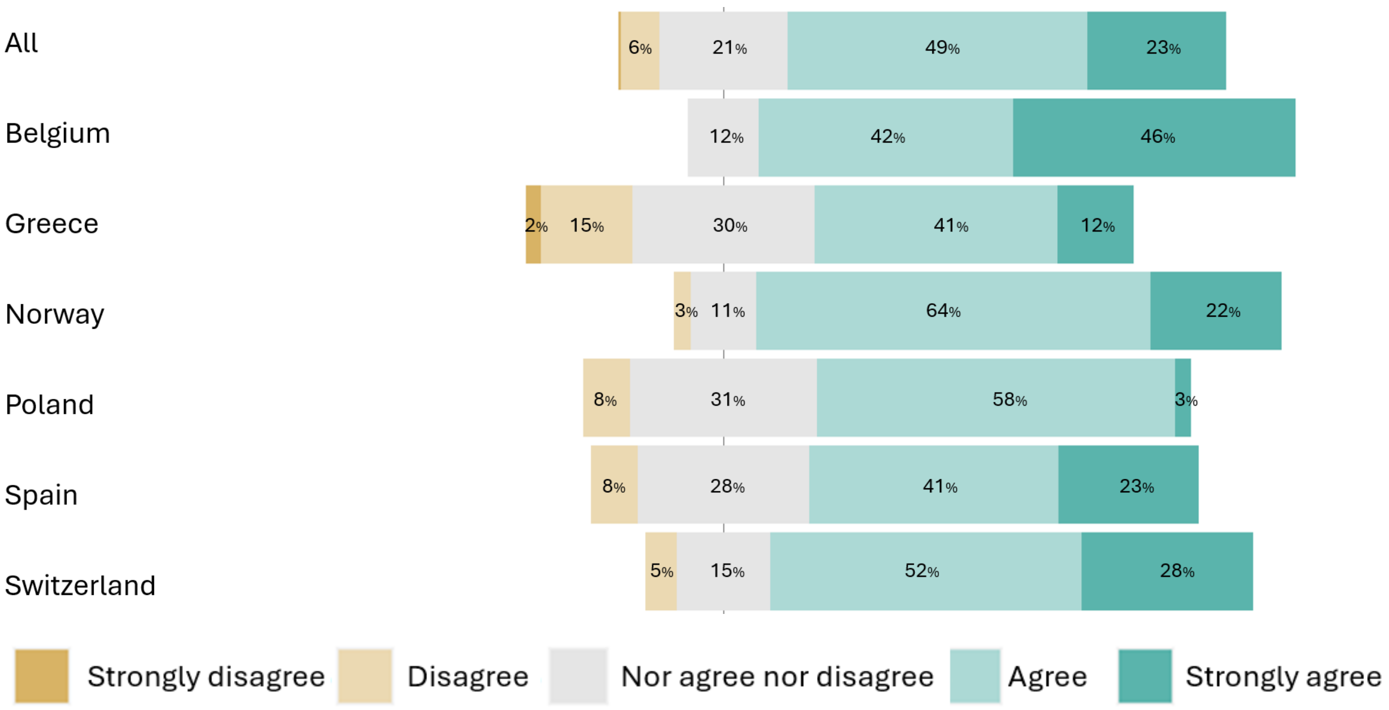

© 2025 Shapoval V et al. *JAMA Network Open*.

If I deprescribe BZRA in older adults, it will negatively affect my relationship with these patients.

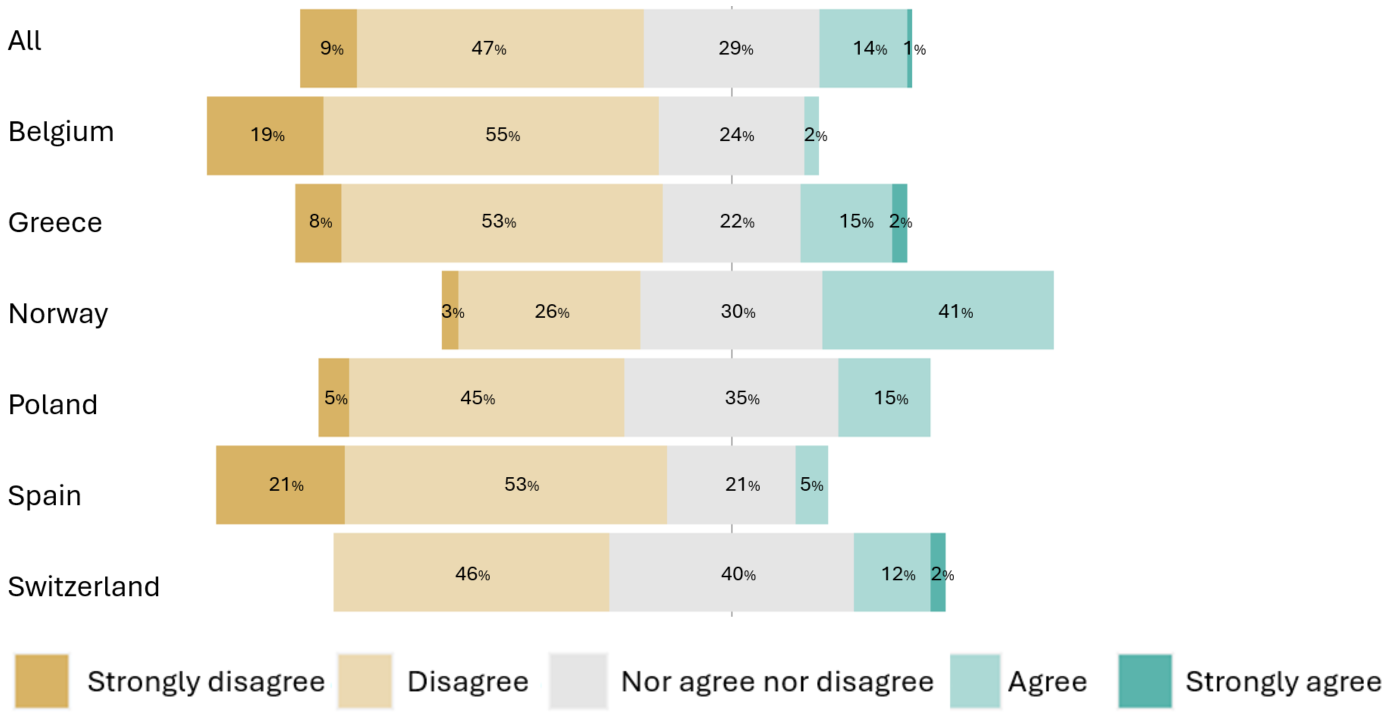

I believe that BZRA deprescribing in older adults will have negative consequences for my patient's health.

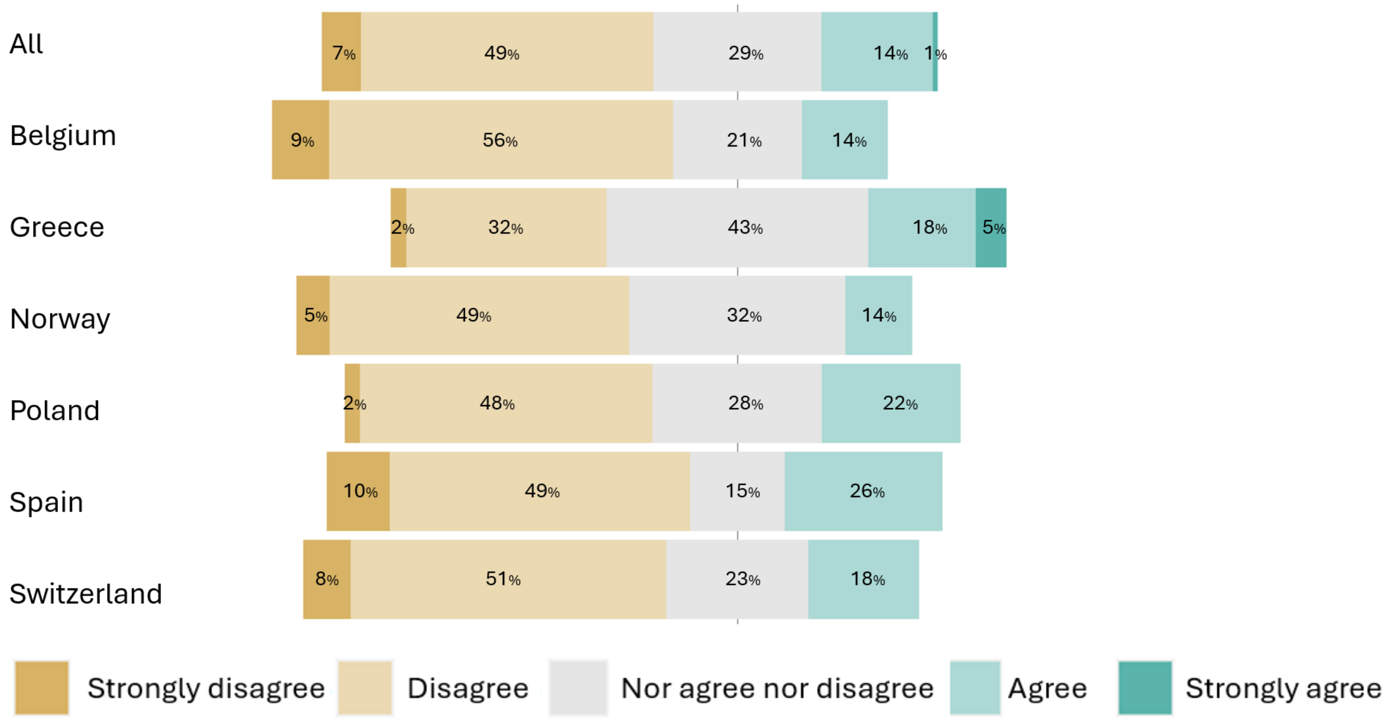

© 2025 Shapoval V et al. *JAMA Network Open*.

In general, I believe that the benefits of deprescribing BZRA in older adults outweigh the harms.

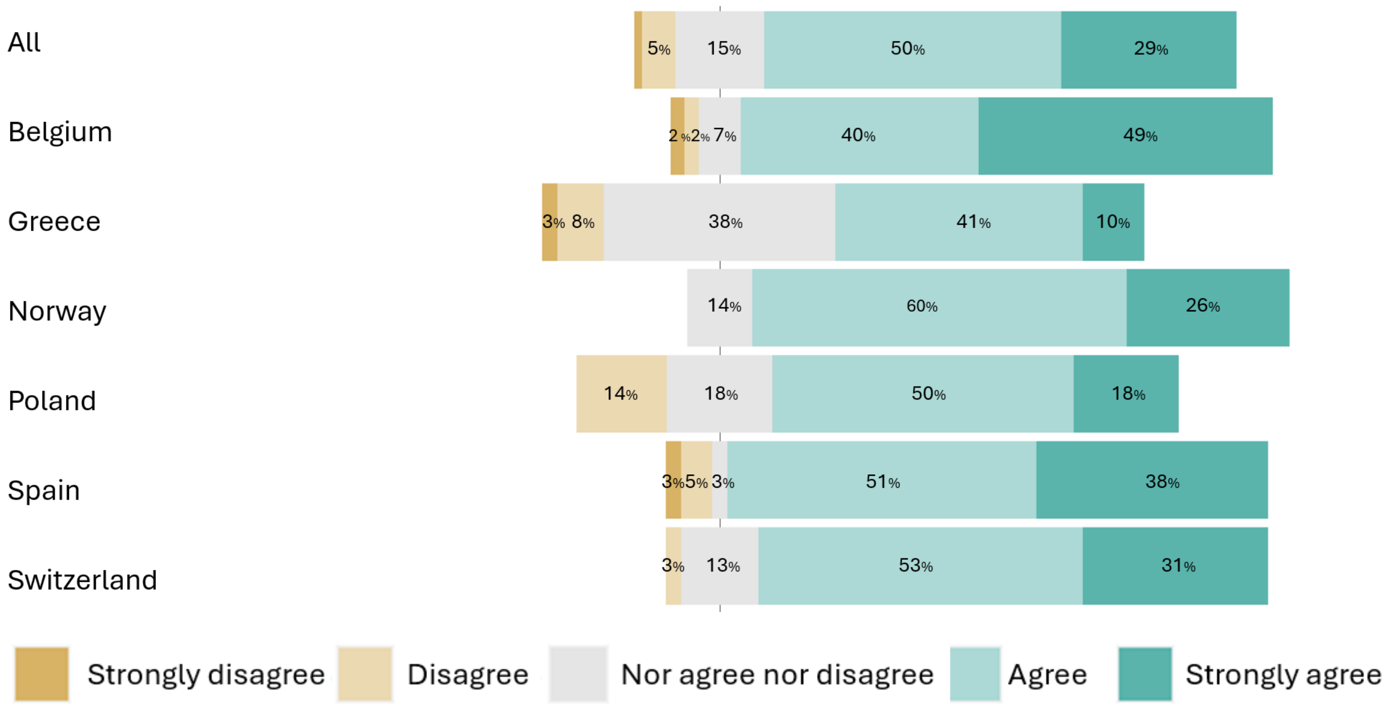

eTable 3.7. Reinforcement

TDF-based items	Mean (SD)					
	Belgium	Greece	Norway	Poland	Spain	Switzerland
I am reluctant to deprescribe BZRA in older adults with sleep problems due to previous failed attempts.	3.53* (1.03)	3.31* (1.03)	3.70* (0.70)	3.21* (0.95)	3.59* (0.94)	3.31* (0.73)

*reversed mean applied to negative statements (so that for all items, the lower the mean, the higher the barrier)

© 2025 Shapoval V et al. *JAMA Network Open*.

I am reluctant to deprescribe BZRA in older adults with sleep problems due to previous failed attempts.

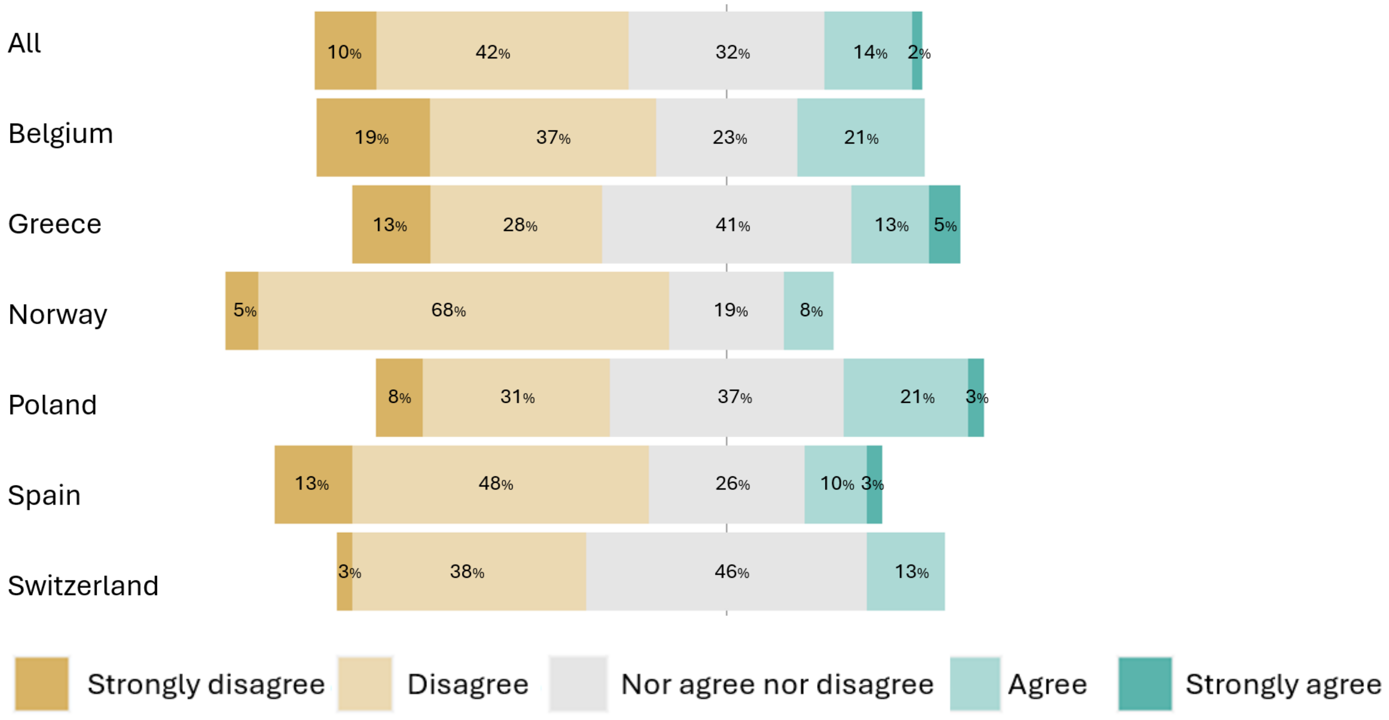

eTable 3.8. Goals

TDF-based items	Mean (SD)					
	Belgium	Greece	Norway	Poland	Spain	Switzerland
Deprescribing BZRA in older adults with sleep problems is a priority for me.	3.30 (0.99)	3.33 (1.13)	2.73 (0.80)	2.73 (0.96)	3.61 (0.86)	3.08 (0.89)
My patients often have other health problems that are usually more important for me to address than the BZRA deprescribing.	2.56* (1.03)	2.49* (0.89)	2.11* (0.88)	2.08* (0.89)	2.68* (1.07)	1.79* (0.69)

*reversed mean applied to negative statements (so that for all items, the lower the mean, the higher the barrier)

© 2025 Shapoval V et al. *JAMA Network Open*.

Deprescribing BZRA in older adults with sleep problems is a priority for me.

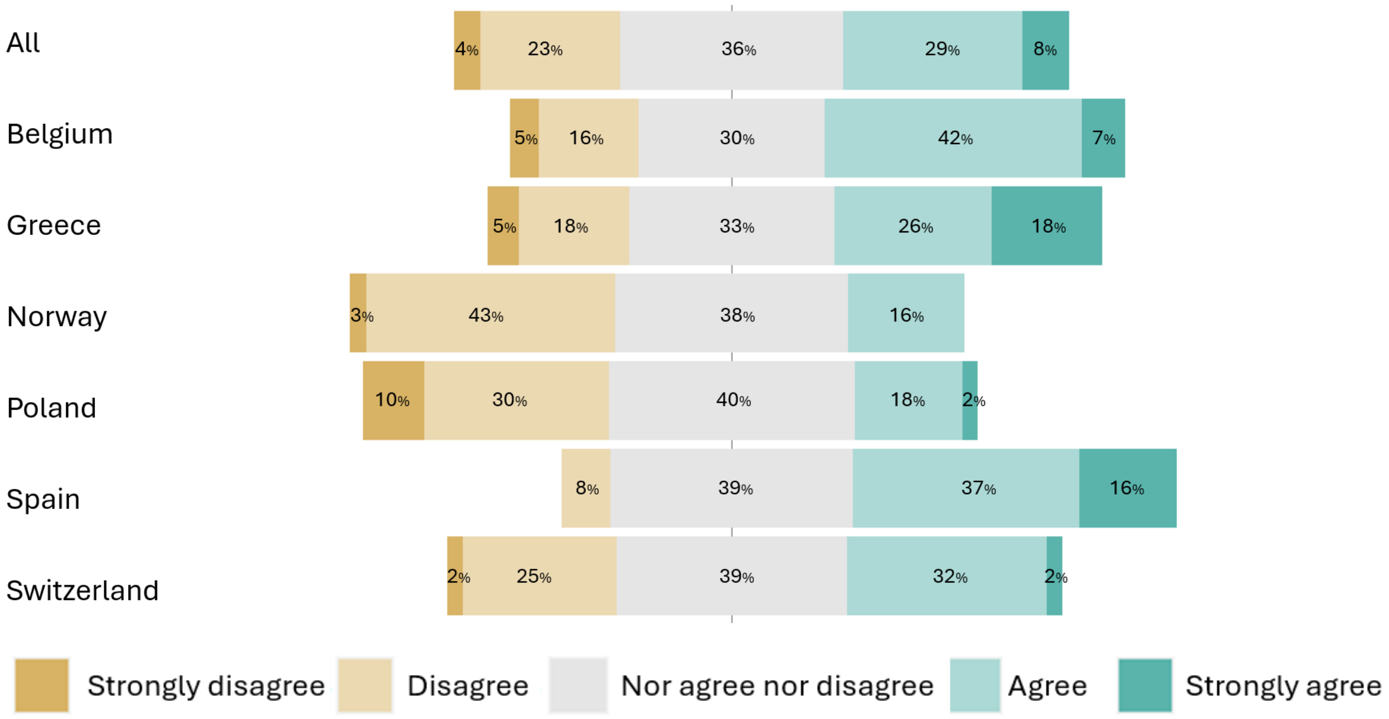

My patients have other health problems more important for me to address than the BZRA deprescribing.

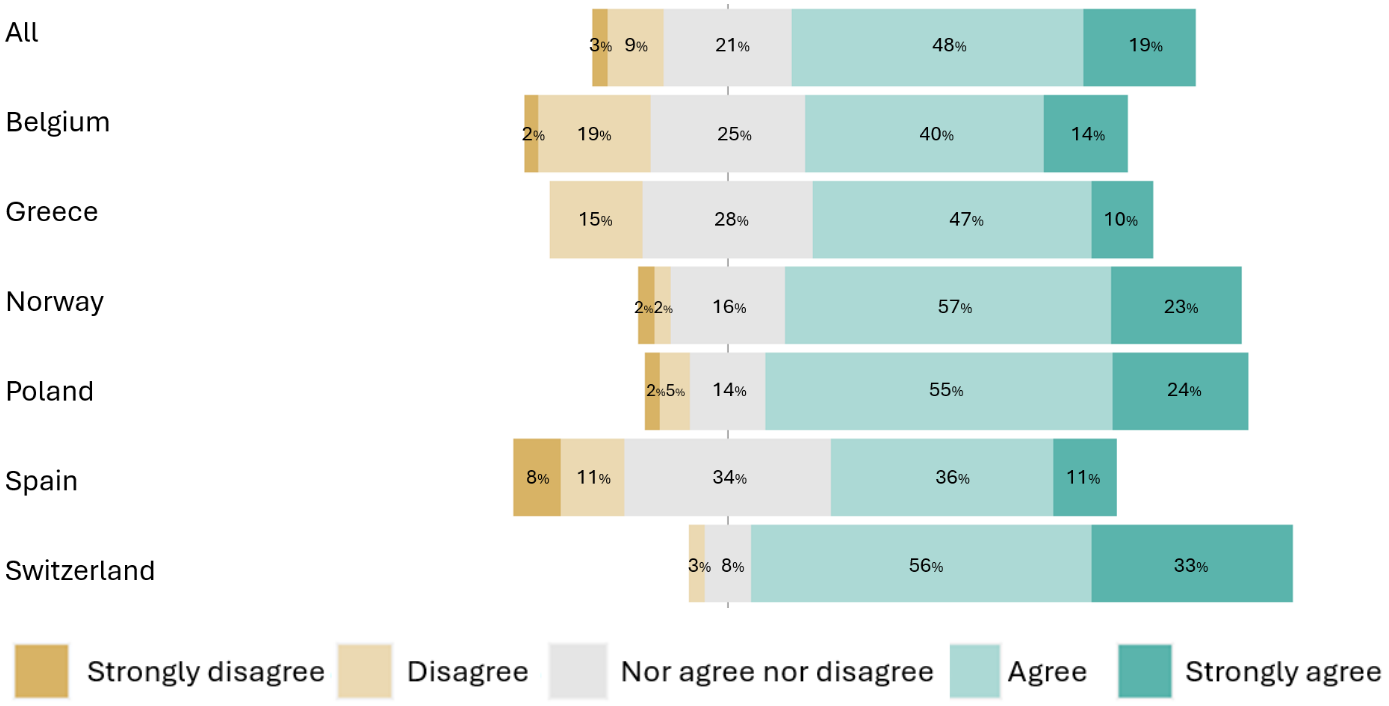

© 2025 Shapoval V et al. *JAMA Network Open*.

eTable 3.9. Intentions

TDF-based items	Mean (SD)					
	Belgium	Greece	Norway	Poland	Spain	Switzerland
I intend to deprescribe BZRA in older adults with sleep problems.	4.09 (0.97)	3.28 (0.86)	3.54 (0.73)	3.49 (0.76)	3.64 (0.96)	3.87 (0.62)
I intend to promote the use of alternative approaches to help older adults deal with sleep problems.	4.07 (0.86)	3.18 (1.19)	3.76 (0.83)	3.89 (0.65)	3.77 (0.92)	4.00 (0.69)

I intend to deprescribe BZRA in older adults with sleep problems.

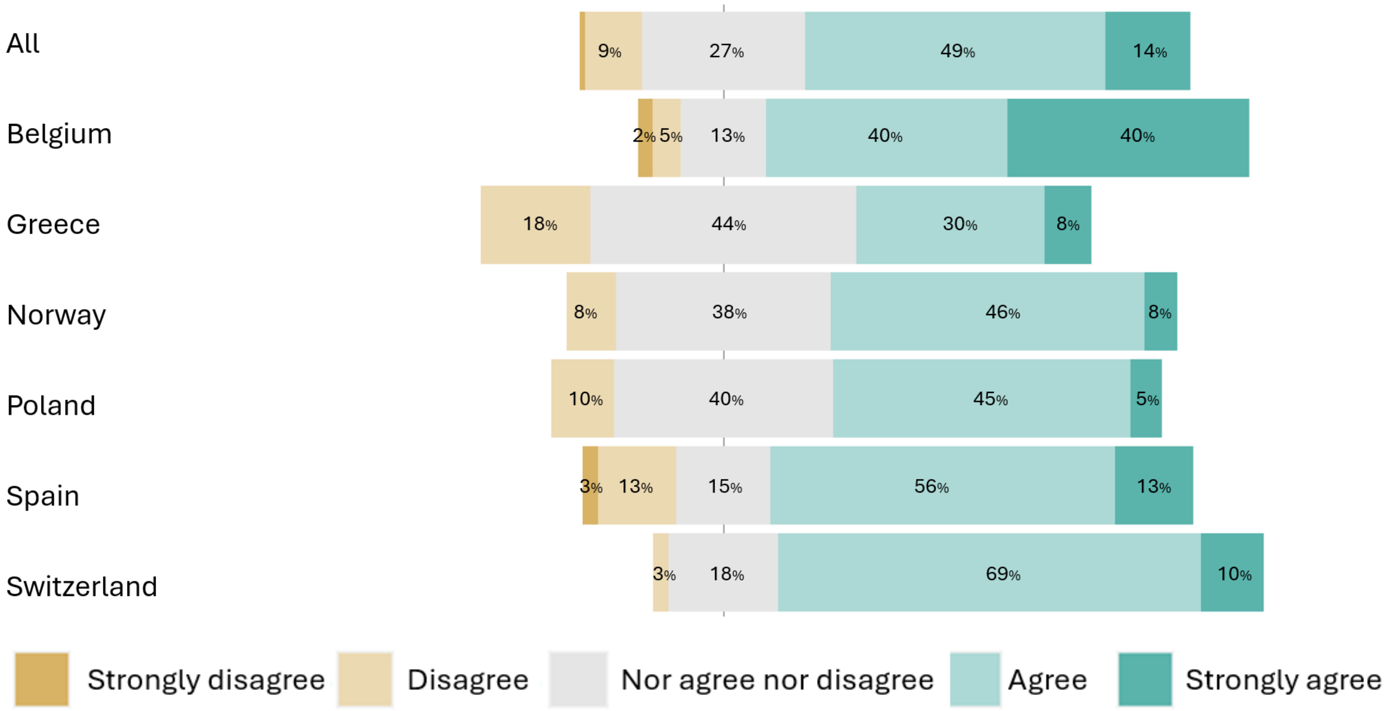

© 2025 Shapoval V et al. *JAMA Network Open*.

I intend to promote the use of alternative approaches to help older adults deal with sleep problems.

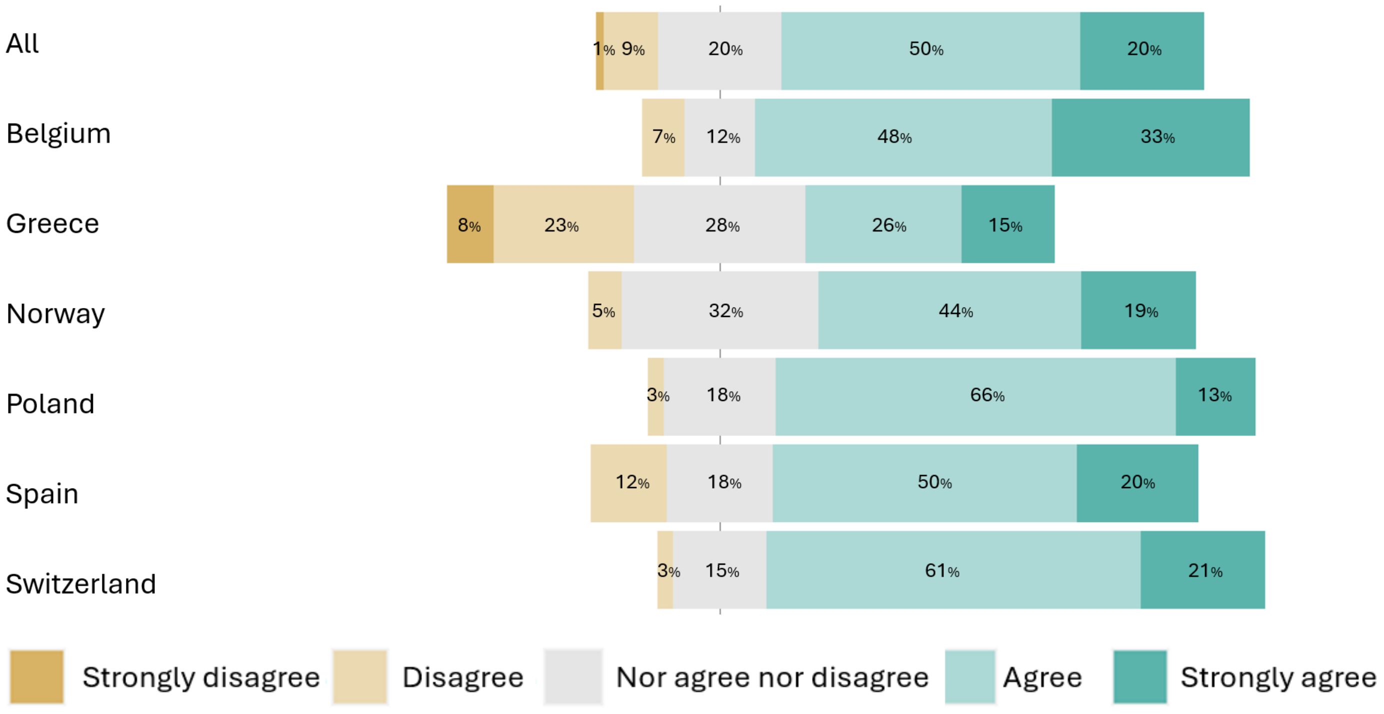

eTable 3.10. Emotions

TDF-based items	Mean (SD)					
	**Belgium**	**Greece**	**Norway**	**Poland**	**Spain**	**Switzerland**
I feel frustrated with all the challenges of BZRA deprescribing in older adults with sleep problems.	2.70* (0.94)	3.00* (1.00)	2.62* (1.11)	2.70* (1.04)	3.67* (0.94)	2.49* (0.76)
I feel stressed about BZRA deprescribing in older adults with sleep problems.	3.81* (0.76)	2.74* (1.04)	3.27* (0.96)	2.85* (0.96)	2.45* (0.93)	3.57* (0.65)

* reversed mean applied to negative statements (so, as that for all items, the lower the mean, the higher the barrier)

© 2025 Shapoval V et al. *JAMA Network Open*.

I feel frustrated with all the challenges of BZRA deprescribing in older adults with sleep problems.

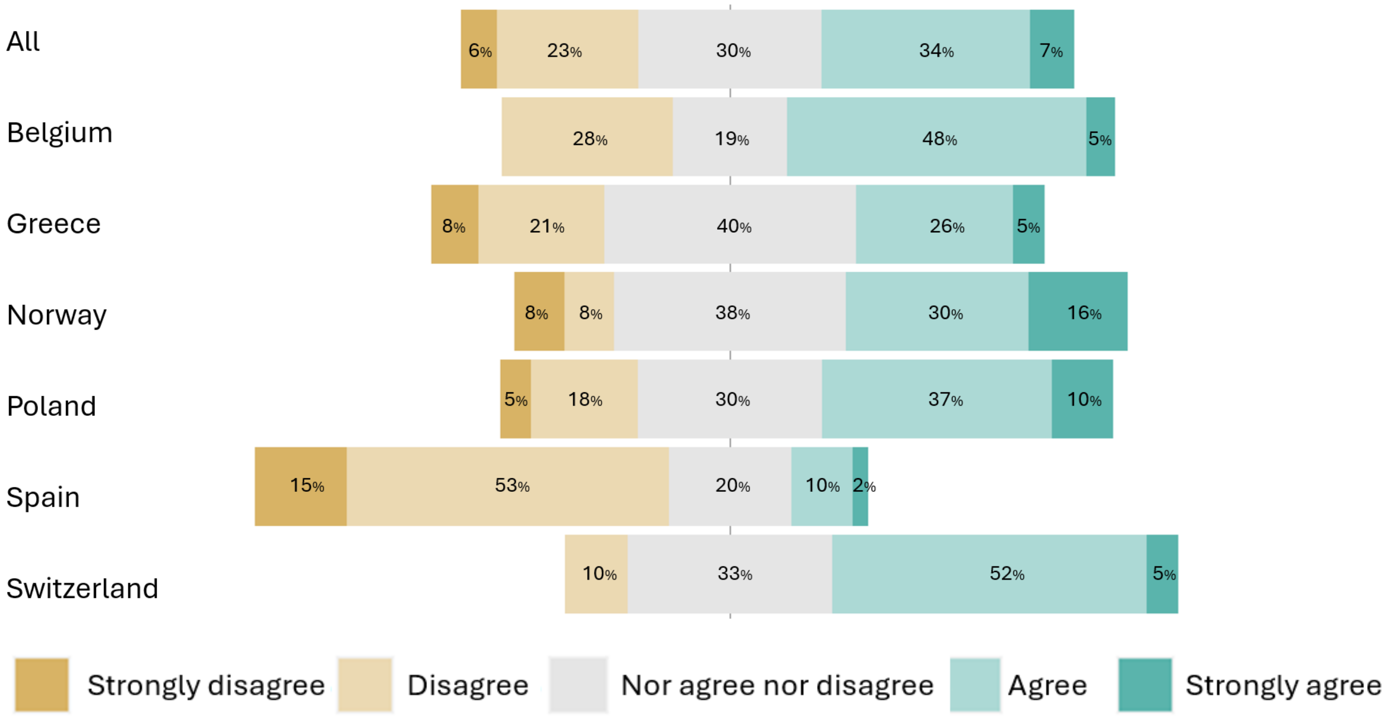

I feel stressed about BZRA deprescribing in older adults with sleep problems.

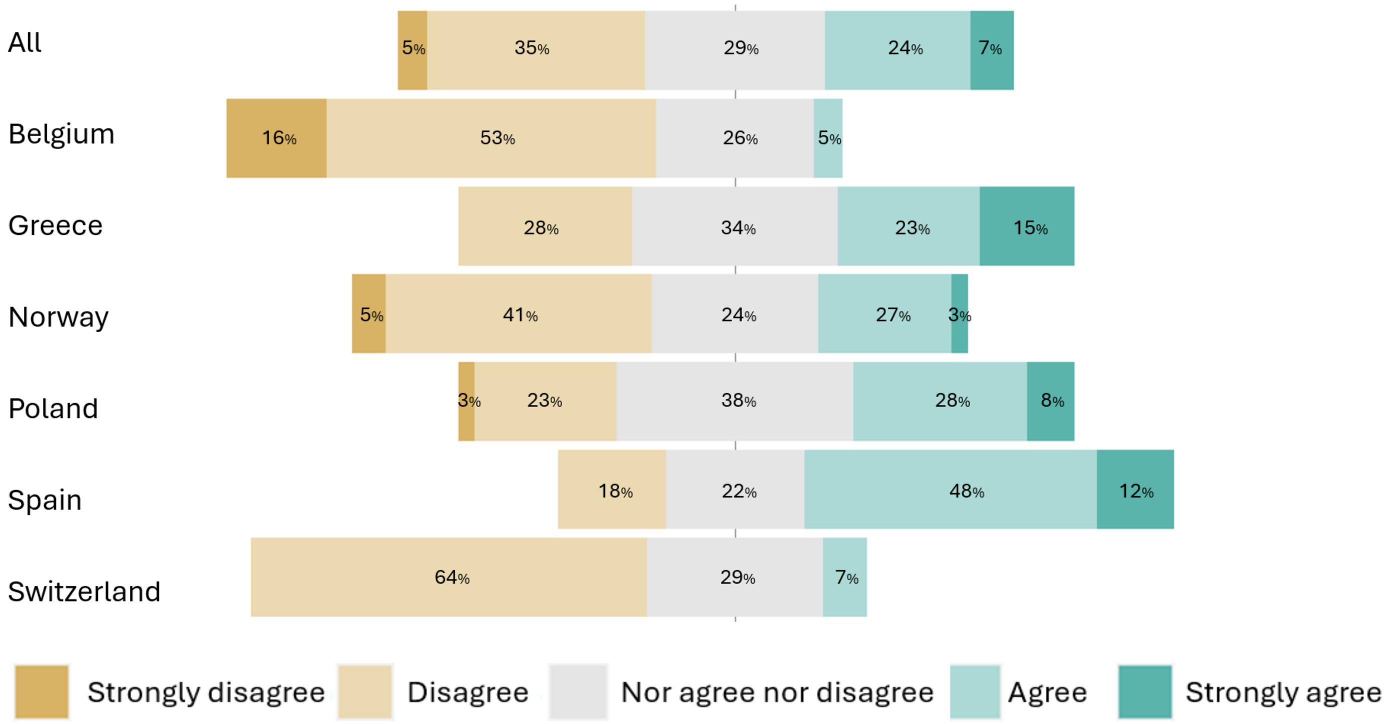

© 2025 Shapoval V et al. *JAMA Network Open*.

eTable 3.11. Environmental context and resources

TDF-based items	Mean (SD)					
	Belgium	Greece	Norway	Poland	Spain	Switzerland
There are guidelines and tools available for BZRA deprescribing in older adults with sleep problems that are possible to implement in my practice.	3.42 (0.88)	3.33 (0.87)	2.84 (0.76)	3.10 (0.94)	2.31 (0.98)	2.93 (0.62)
I have enough time to educate and inform patients about BZRA deprescribing.	2.44 (1.12)	2.79 (1.10)	2.46 (1.12)	2.00 (0.90)	2.71 (1.04)	2.26 (0.88)
There is enough staff in the department or institution where I work to support BZRA deprescribing.	2.95 (1.05)	2.26 (0.94)	2.62 (1.14)	2.30 (1.07)	3.59 (0.91)	2.88 (1.07)
In my opinion, BZRA deprescribing is not prioritized by our healthcare system.	3.86* (1.15)	2.64* (1.25)	2.30* (1.02)	2.70* (1.16)	2.23* (0.87)	2.26* (0.85)
In the department or institution where I work, we have set goals (or policies) that encourage BZRA deprescribing.	2.93 (1.08)	2.62 (1.16)	2.43 (0.93)	2.17 (0.96)	2.58 (1.11)	3.38 (0.88)

*reversed mean applied to negative statements (so that for all items, the lower the mean, the higher the barrier)

There are guidelines/tools available for BZRA deprescribing in older adults implementable in my practice.

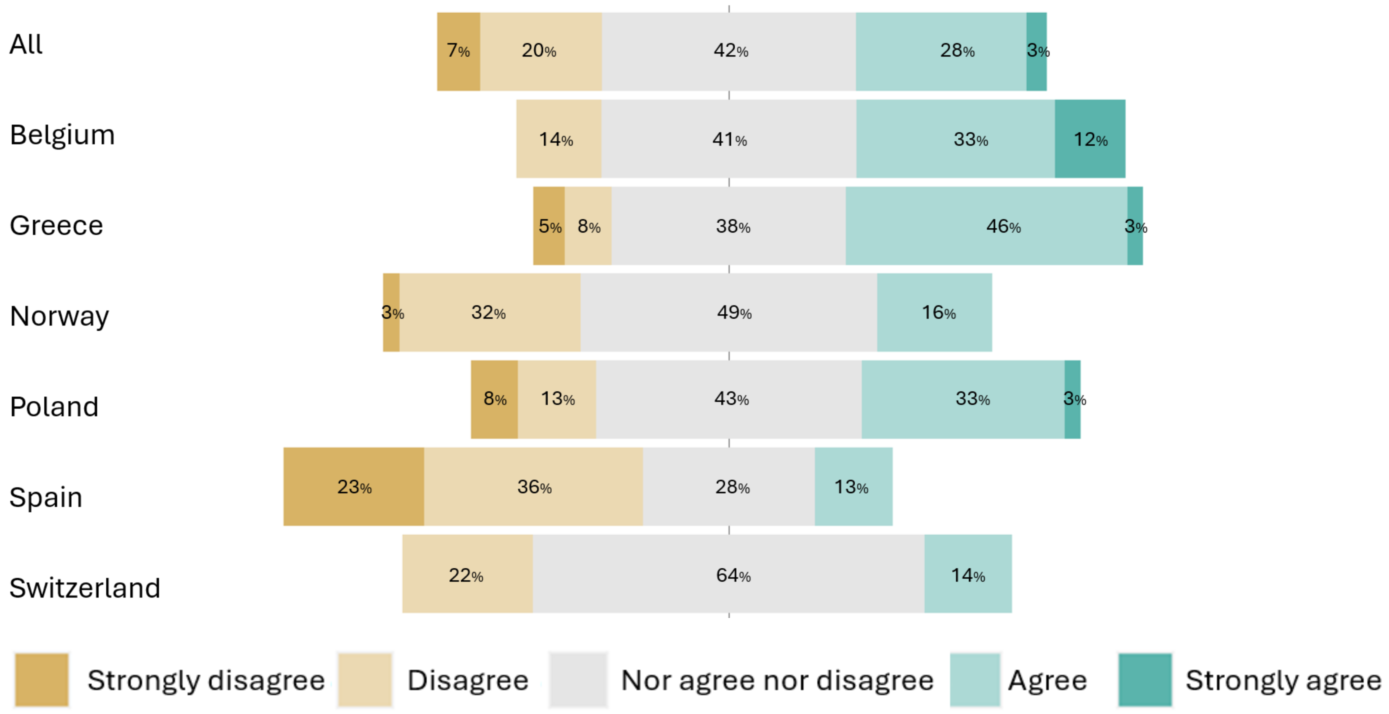

© 2025 Shapoval V et al. *JAMA Network Open*.

I have enough time to educate and inform patients about BZRA deprescribing.

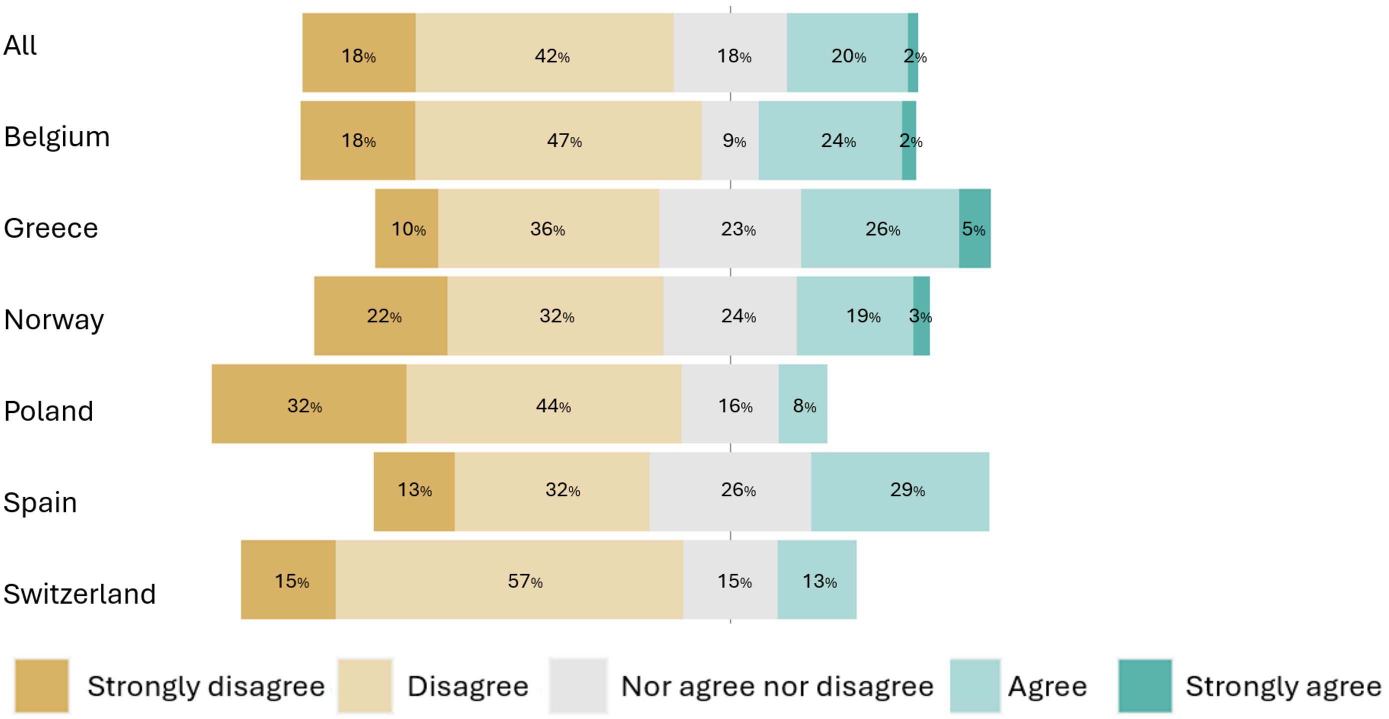

There is enough staff in the department or institution where I work to support BZRA deprescribing.

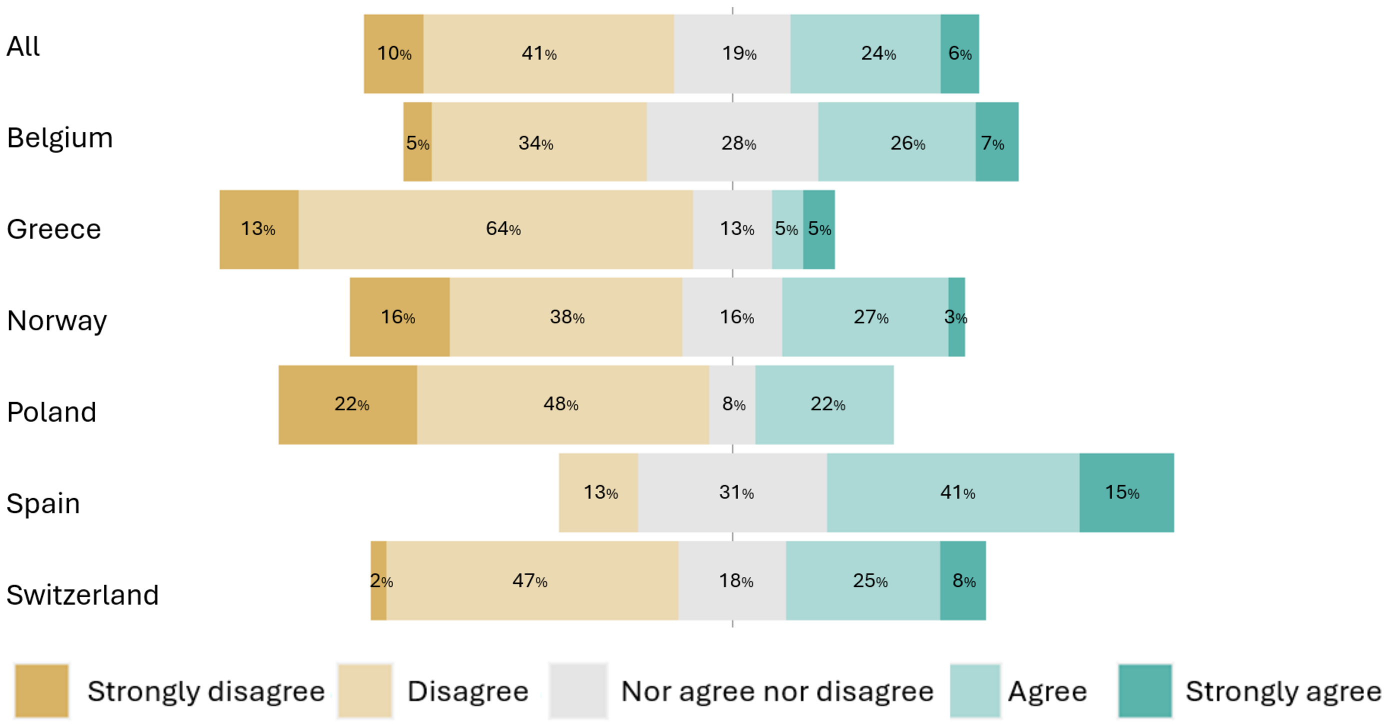

© 2025 Shapoval V et al. *JAMA Network Open*.

In my opinion, BZRA deprescribing is not prioritized by our healthcare system.

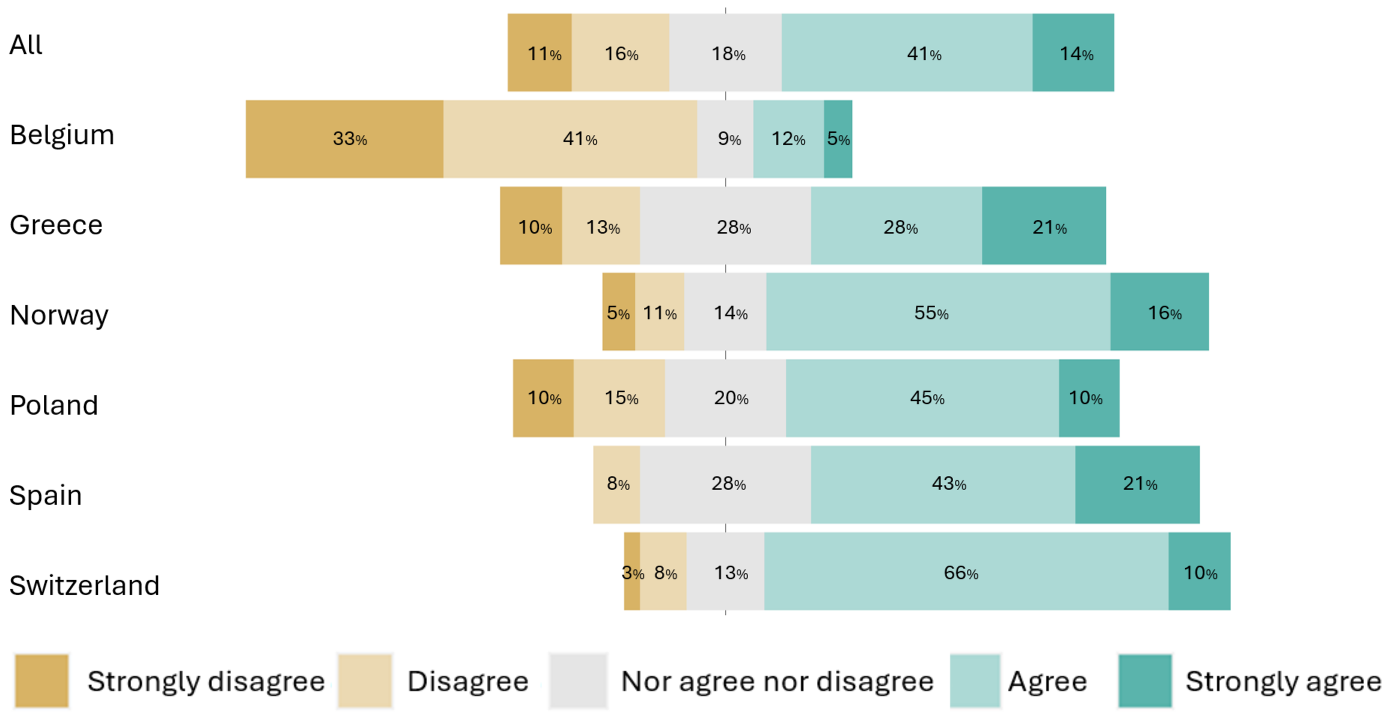

In the department or institution where I work, we have set goals that encourage BZRA deprescribing.

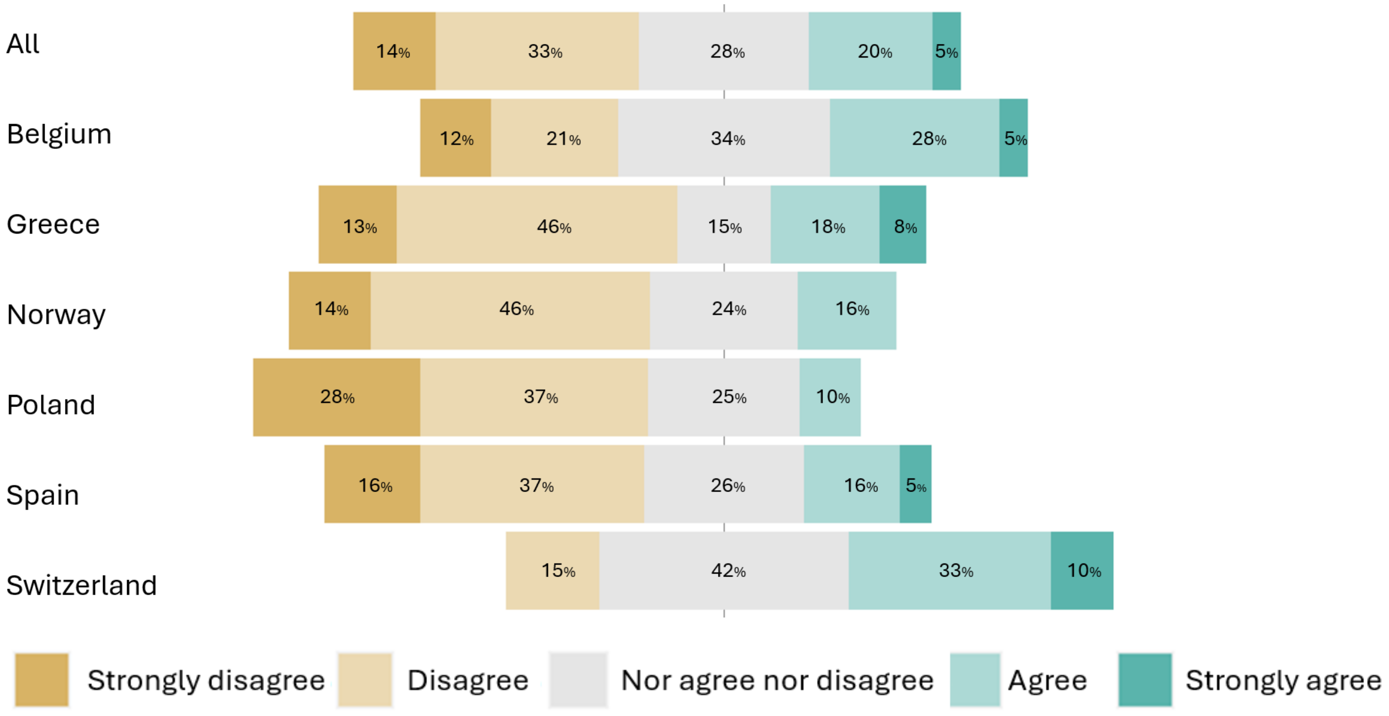

© 2025 Shapoval V et al. *JAMA Network Open*.

eTable 3.12. Social influence

TDF-based items	Mean (SD)					
	Belgium	Greece	Norway	Poland	Spain	Switzerland
I feel a lot of pressure from older adults with sleep problems and/or their relatives to renew/extend their prescriptions.	2.19* (0.94)	2.46* (0.97)	2.35* (0.89)	2.23* (0.87)	2.42* (0.92)	2.46* (0.92)
Most of my older patients take a BZRA for sleep problems or their relatives are reluctant to deprescribe their BZRA.	2.02* (0.71)	2.19* (0.97)	2.19* (0.81)	2.15* (0.84)	2.26* (0.79)	2.31* (0.83)
My colleagues/collaborators whose opinions I value support BZRA deprescribing in older adults with sleep problems.	4.21 (0.68)	3.08 (0.90)	3.86 (0.82)	3.31 (0.86)	3.92 (0.912)	3.77 (0.74)
My colleagues are supportive of BZRA deprescribing.	4.10 (0.91)	2.97 (1.11)	3.89 (0.81)	3.33 (0.93)	3.67 (0.87)	3.69 (0.77)

*reversed mean applied to negative statements (so that for all items, the lower the mean, the higher the barrier)

I feel a lot of pressure from older adults and/or their relatives to renew/extend their prescriptions.

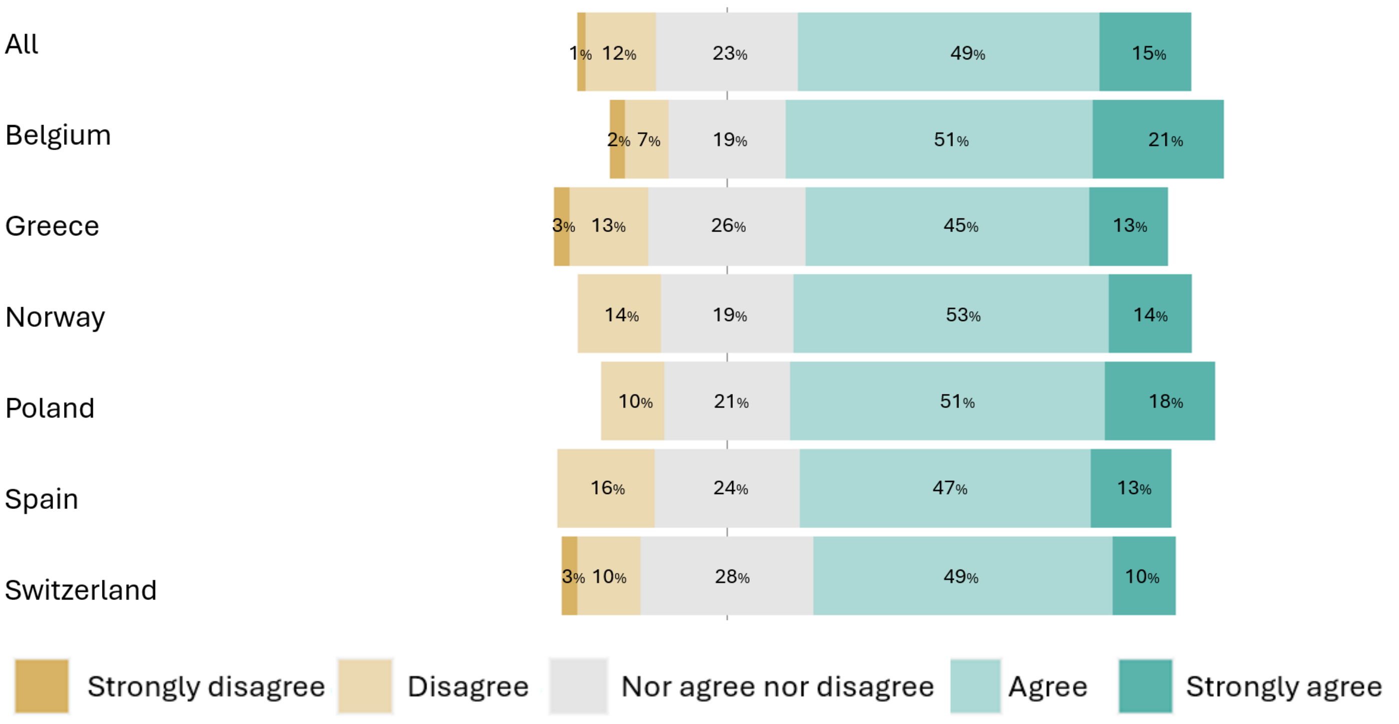

© 2025 Shapoval V et al. *JAMA Network Open*.

Most of my older patients who take a BZRA, or their relatives, are reluctant to deprescribe their BZRA.

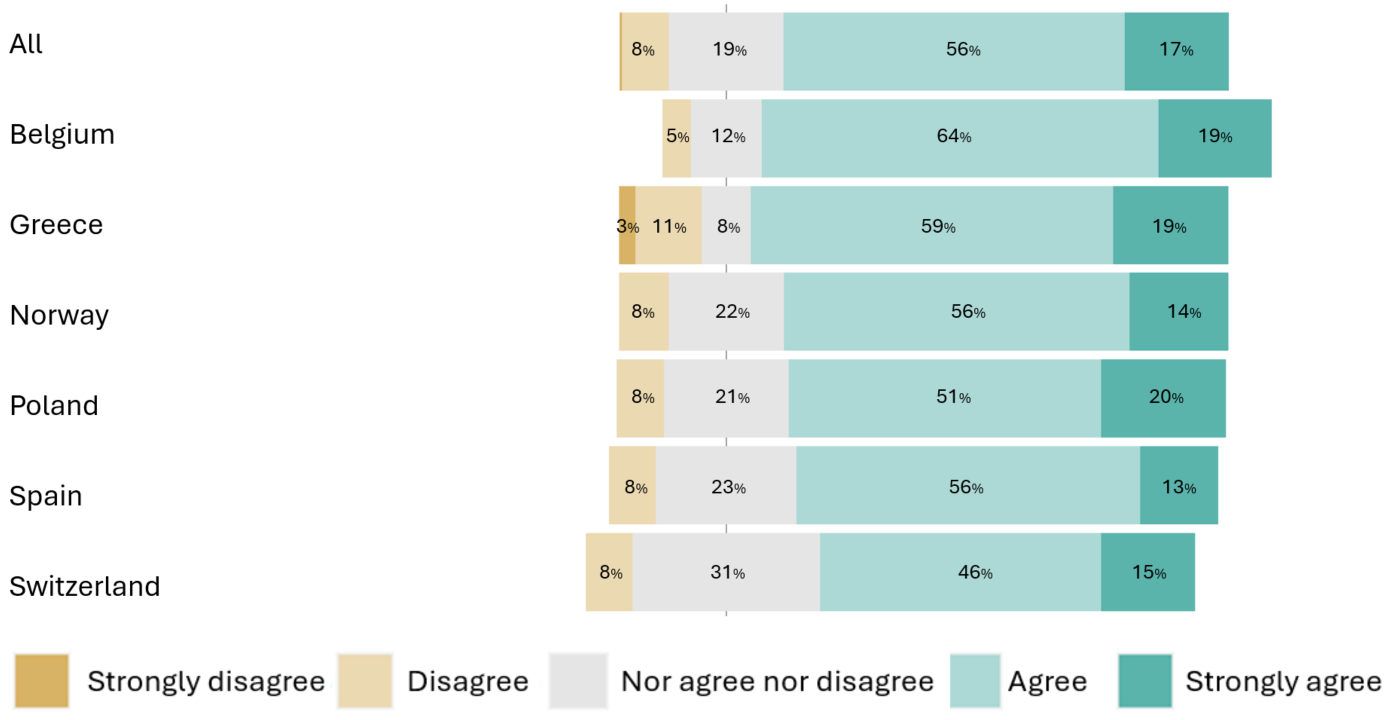

My colleagues/collaborators whose opinions I value support BZRA deprescribing in older adults.

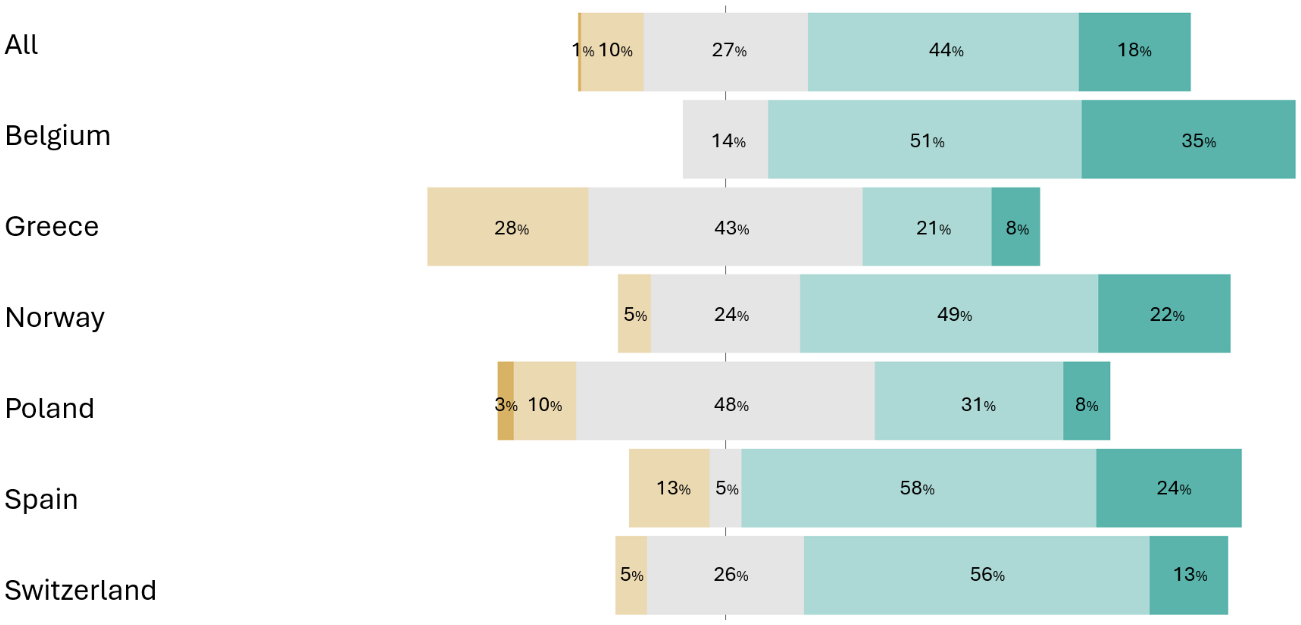

© 2025 Shapoval V et al. *JAMA Network Open*.

My colleagues are supportive of BZRA deprescribing.

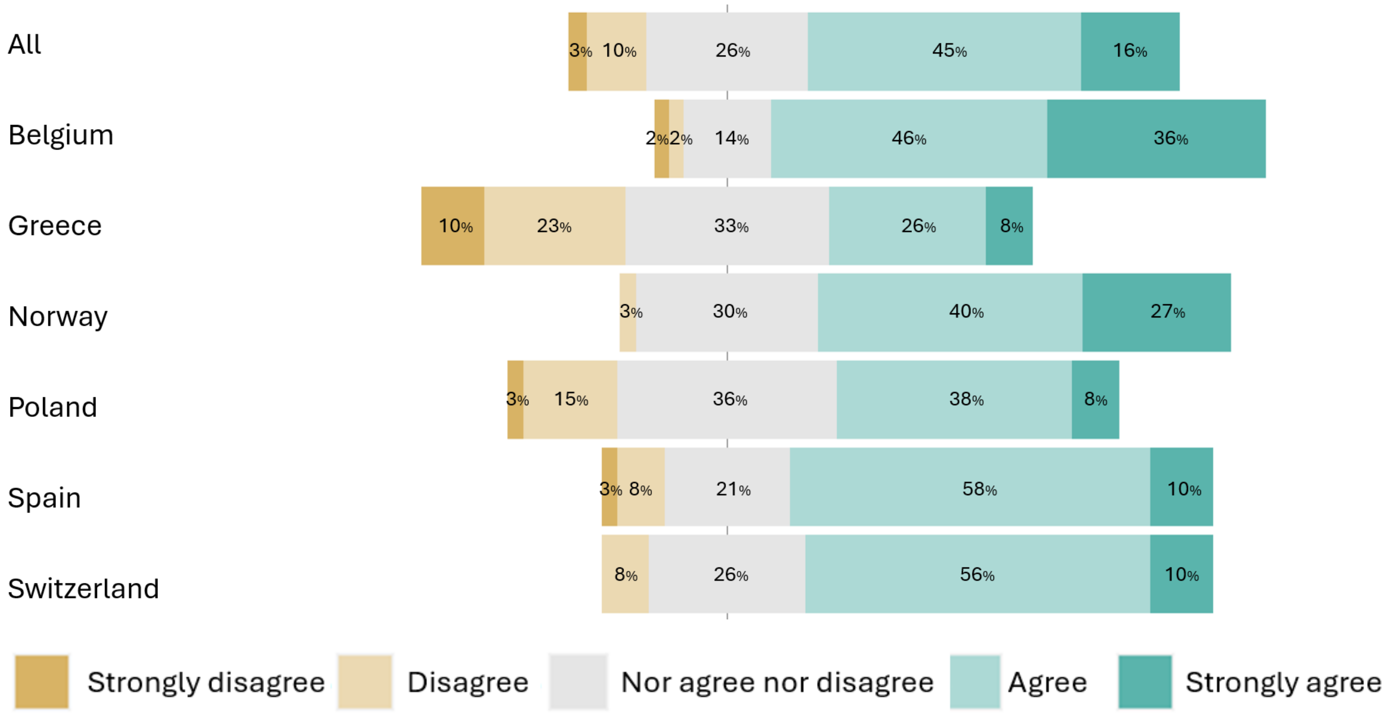

© 2025 Shapoval V et al. *JAMA Network Open*.

eAppendix 1. Summary of answers to open questions on barriers of and enablers to BZRA deprescribing (hospital physicians.)

In collaboration with partners, the statements were translated from the local language into English and deductively coded according to the Theoretical Domains Framework.

The most cited TDF domain was "Environmental context and resources." Physicians reported a lack of time and stated that short hospitalization was a barrier to BZRA deprescribing. This also led to a lack of follow-up of patients and poor relationships between physicians and patients.

- *"I have too much work and not enough time to work well"* (Pneumologist)
- *"Usually short appointment times with patients, insufficient for a thorough conversation and explanation of the harmful effects of BZRA."* (Cardiologist)
- *"I do not have the opportunity to follow them up after discharge, and it is expected that the GP follows them up."* (Geriatrician)
- *"Lack of regular support in BZRA deprescribing. One visit every 6 months is not sufficient to support the patient."* (Neurologist)

Physicians also reported poor coordination between inpatient and ambulatory care.

- *"Lack of cooperation between individuals involved in the process of BZRA deprescribing, such as between the family doctor, psychiatrist, and neurologist."* (Neurologist)
- *"Lack of contact with primary care for follow-up and non-renewal of prescription"* (Internist)

The domain "social influence" was also frequently cited by physicians. They evoked patients' reluctance, as well as a lack of collaboration among physicians who renew BZRA prescriptions. Nursing staff also was cited as a potential influence.

- *"Patients reluctant to try any intervention other than medication."* (Other)
- *"Even if as a specialist doctor I deprescribe a BZRA, it is often useless because the GP or another doctor renews the prescription."* (Internist)
- *"Nurse says that the patient MUST have something (read: must have some prescription medicine)."* (Internist)
- *"In hospital settings, one of the barriers to deprescribing comes from nursing. Elderly patients with sleep difficulties regularly request their sleeping pill or are older, which is an important work overload for understaffed teams during the night."* (Other)

Patients' conditions, including acute situations, psychiatric issues, and cognitive impairments also seemed to impact physicians' behavior.

- *"Difficult to use motivational interviewing and cognitive therapy if the patient has cognitive impairment. They may lack insight into the problem and be determined that no medication should be changed."* (Geriatrician)

In enablers, physicians mostly pointed to the resolution of the barriers mentioned before. Even though some insights may be considered.

- "*It favors the risk of falls (leading to hospitalization), and the risk of cognitive troubles. Those arguments are sometimes useful in patients who can still understand those explanations. This is also a very big cost for Belgian public health (direct costs with the medications and indirect with the falls and their consequences). Note the significant risk of dependency that can lead to psycho-social isolation*." (Neurologist)

- "*In the pocket" precise schema adapted to the molecule – nonpharmacological alternatives - give the right explanations on the evolution of sleep during aging*." (Geriatrician)

© 2025 Shapoval V et al. *JAMA Network Open*.

eTable 4. Barriers to and enablers of BZRA deprescribing by TDF-based domains and items for general practitioners: country-specific data.

The color code for the tables below		
Enabler (mean ≥ 4.00)	Moderate Barrier (mean 3.00-3.99)	Major Barrier (mean ≤ 2.99)

eTable 4.1. Knowledge

TDF-based items	Mean (SD)				
	Belgium	Greece	Poland	Spain	Switzerland
I know the risks associated with the use of BZRA in older adults with sleep problems.	4.47 (0.51)	4.42 (0.69)	4.44 (0.51)	4.63 (0.50)	4.65 (0.49)
I know the situations or comorbidities in which BZRA deprescribing is not recommended.	2.89 (0.90)	3.95 (0.71)	3.06 (1.03)	3.50 (1.15)	3.40 (0.88)
I know how to taper BZRA in older adults with sleep problems.	3.89 (0.47)	3.53 (0.84)	2.94 (0.97)	4.19 (0.75)	4.20 (0.53)
I know how to engage patients about BZRA deprescribing.	3.95 (0.69)	4.11 (0.74)	3.11 (0.88)	3.88 (0.81)	3.85 (0.88)
I am aware of alternative approaches to deal with sleep problems in older adults.	4.06 (0.24)	3.53 (0.91)	3.29 (0.96)	3.75 (1.06)	4.10 (0.55)

I know the risks associated with the use of BZRA in older adults with sleep problems.

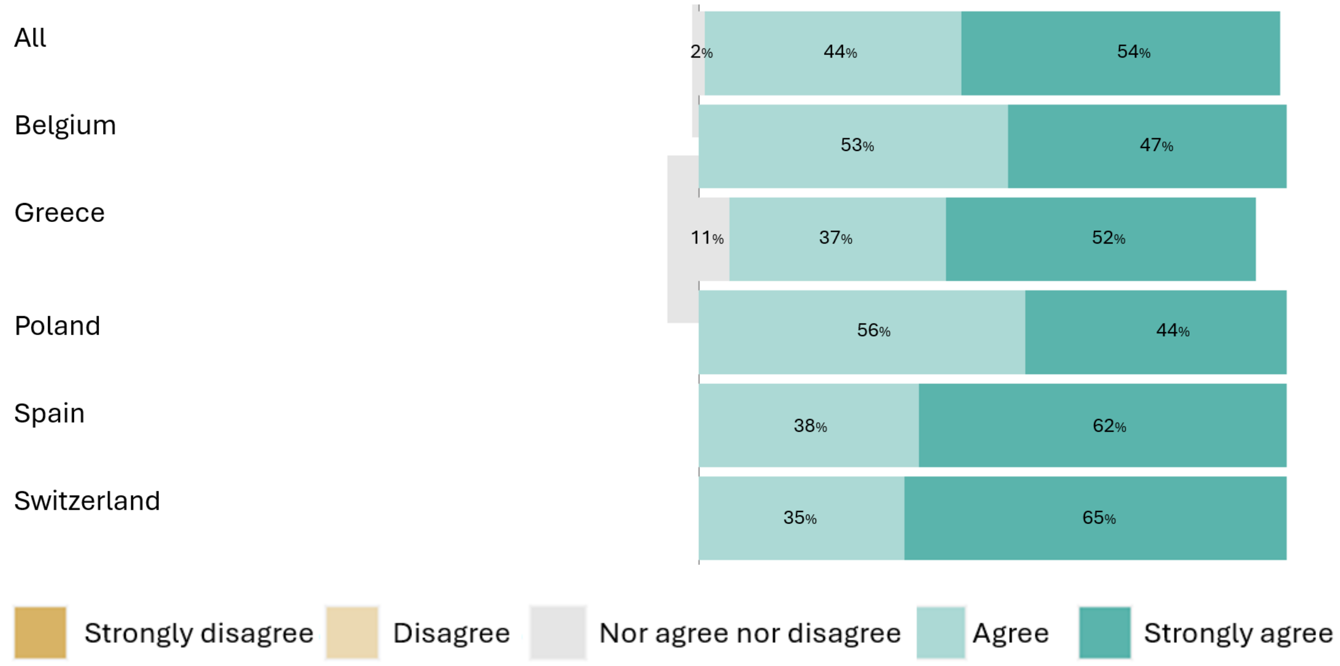

© 2025 Shapoval V et al. *JAMA Network Open*.

I know the situations or comorbidities in which BZRA deprescribing is not recommended.

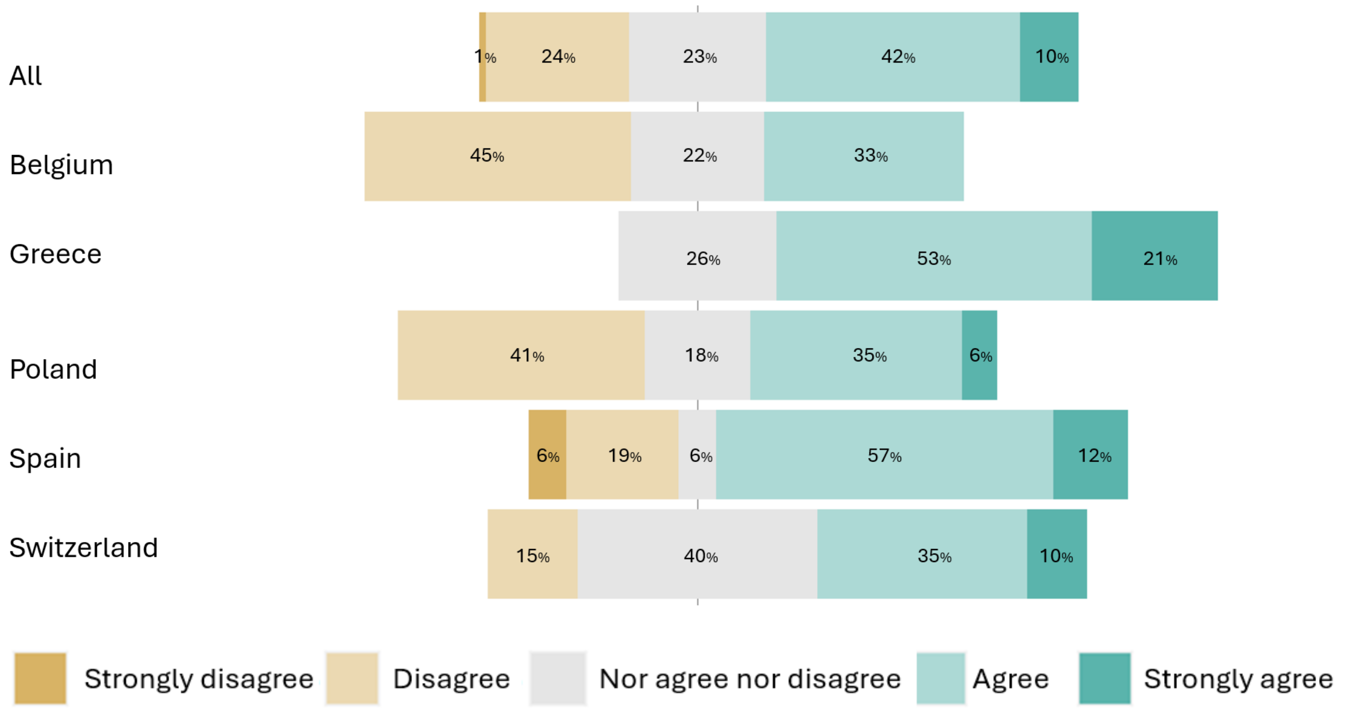

I know how to taper BZRA in older adults with sleep problems.

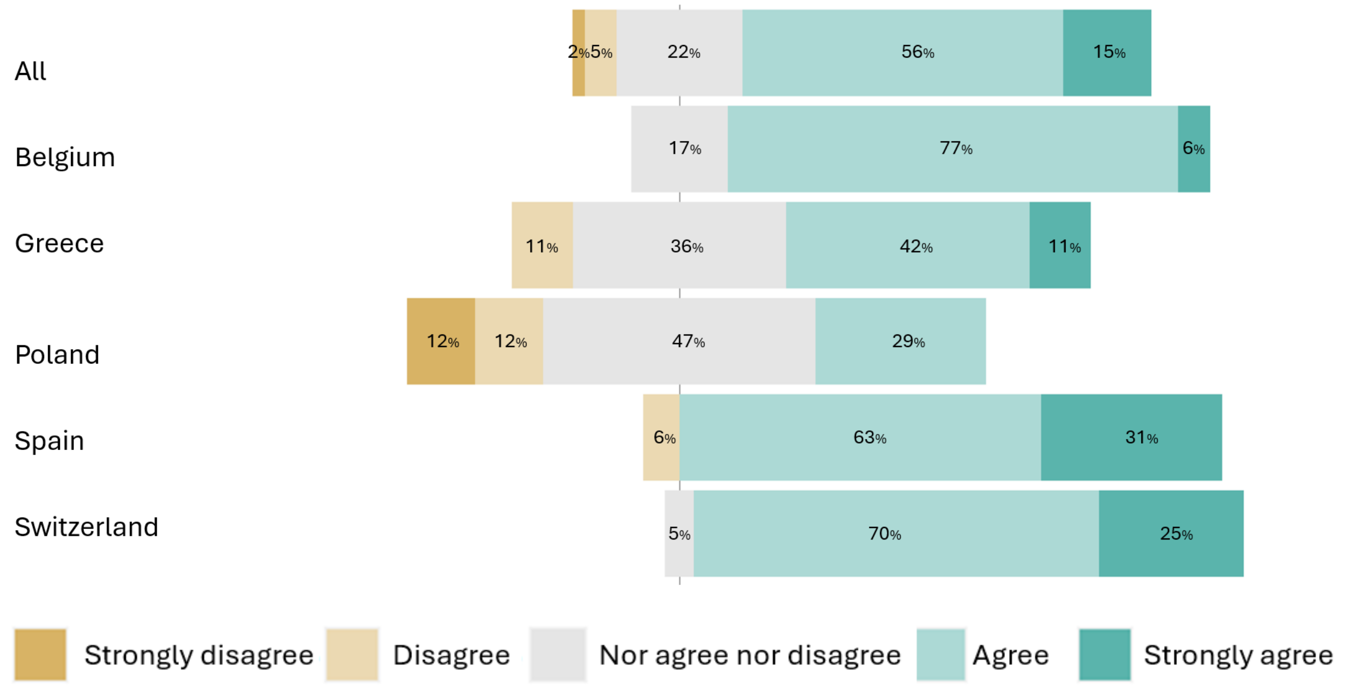

I know how to engage patients about BZRA deprescribing.

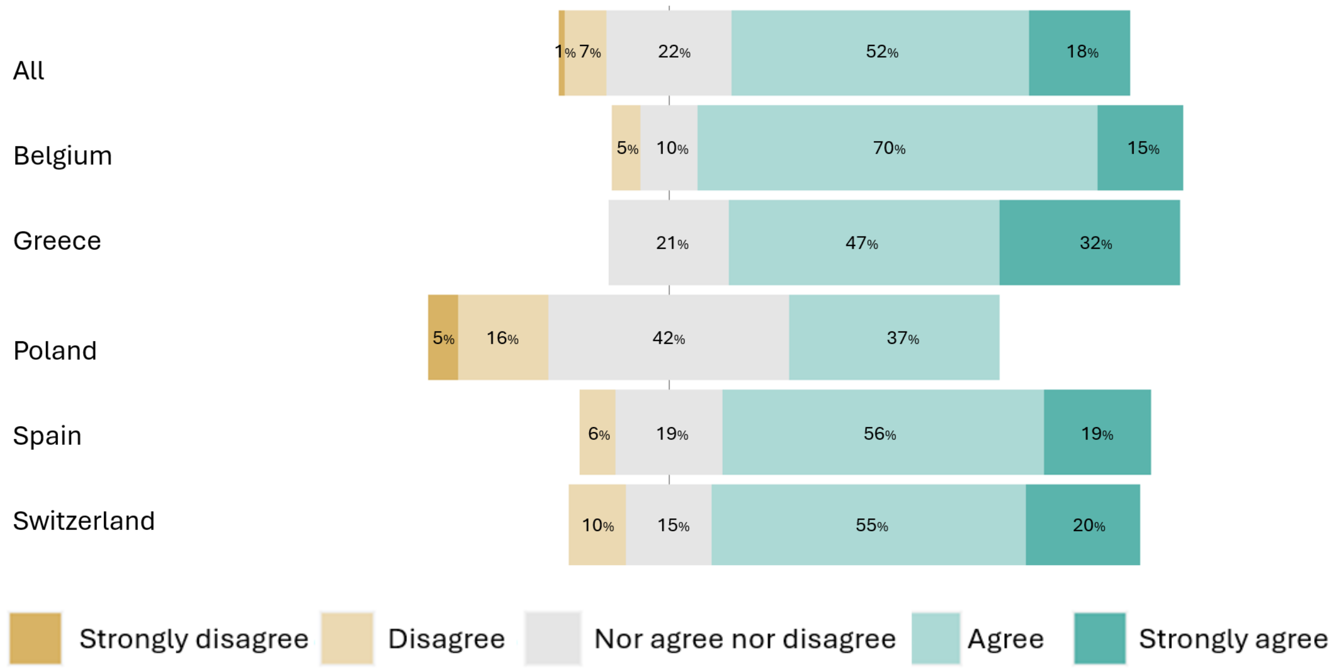

I am aware of alternative approaches to deal with sleep problems in older adults.

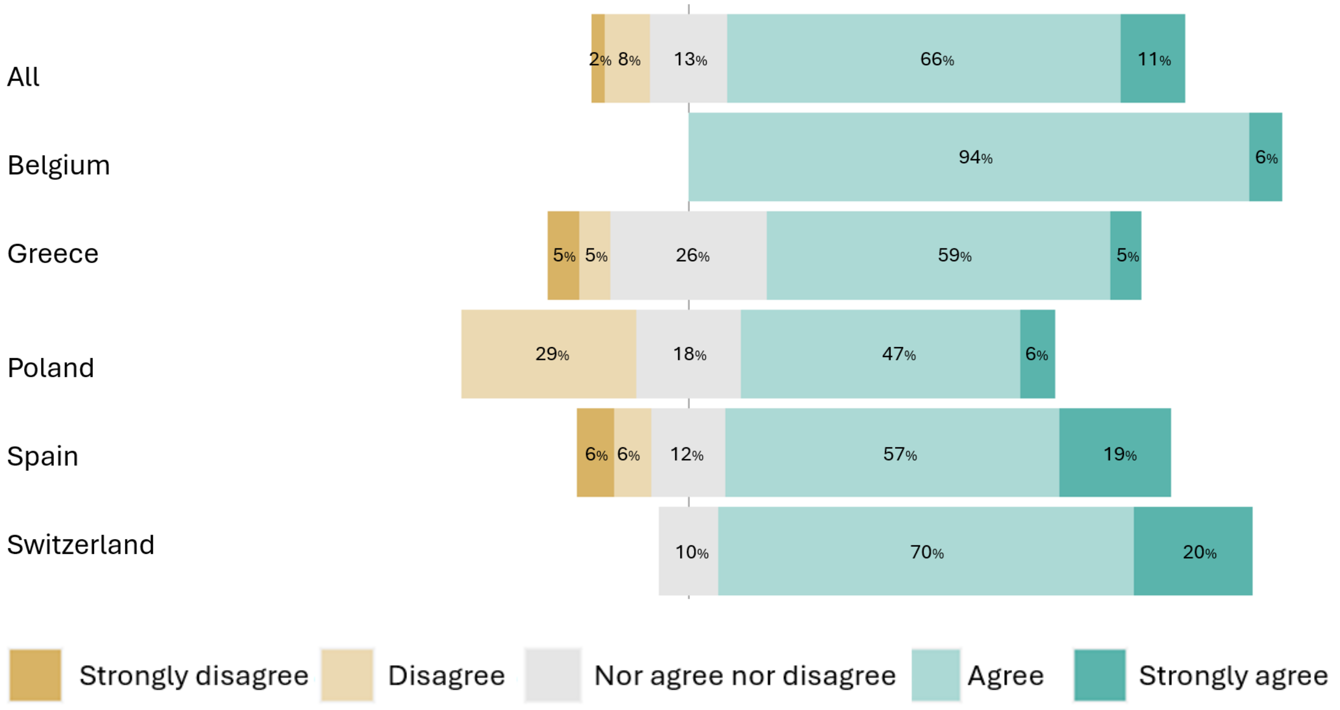

eTable 4.2. Skills

TDF-based items	Mean (SD)				
	Belgium	Greece	Poland	Spain	Switzerland
I have been trained on how to deprescribe BZRA in older adults with sleep problems.	3.39 (1.20)	2.89 (1.10)	1.69 (0.79)	3.13 (1.09)	3.25 (1.29)
I have been trained to engage patients about BZRA deprescribing.	2.83 (1.25)	2.58 (1.07)	1.82 (0.81)	2.75 (1.06)	3.00 (1.21)
I have been trained to implement alternative approaches for sleep problems in older adults.	3.11 (1.23)	2.63 (0.96)	2.47 (1.33)	2.38 (1.09)	3.45 (1.05)

I have been trained on how to deprescribe BZRA in older adults with sleep problems.

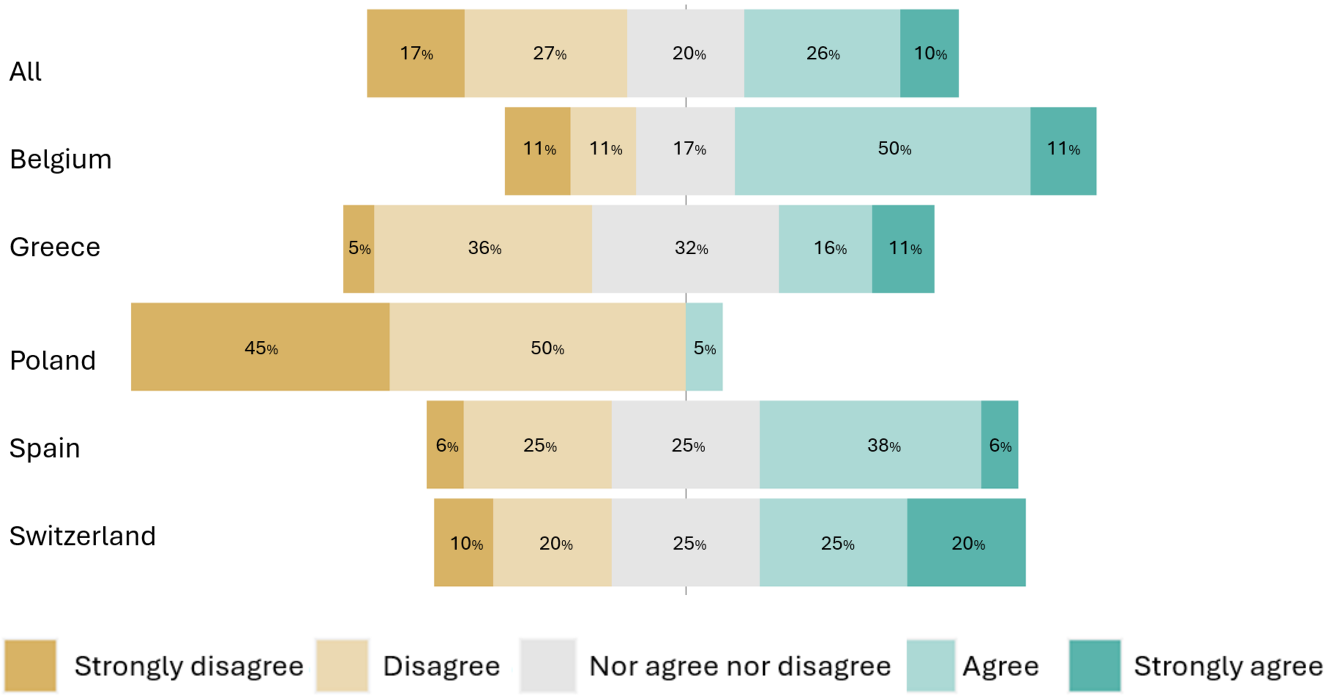

I have been trained to engage patients about BZRA deprescribing.

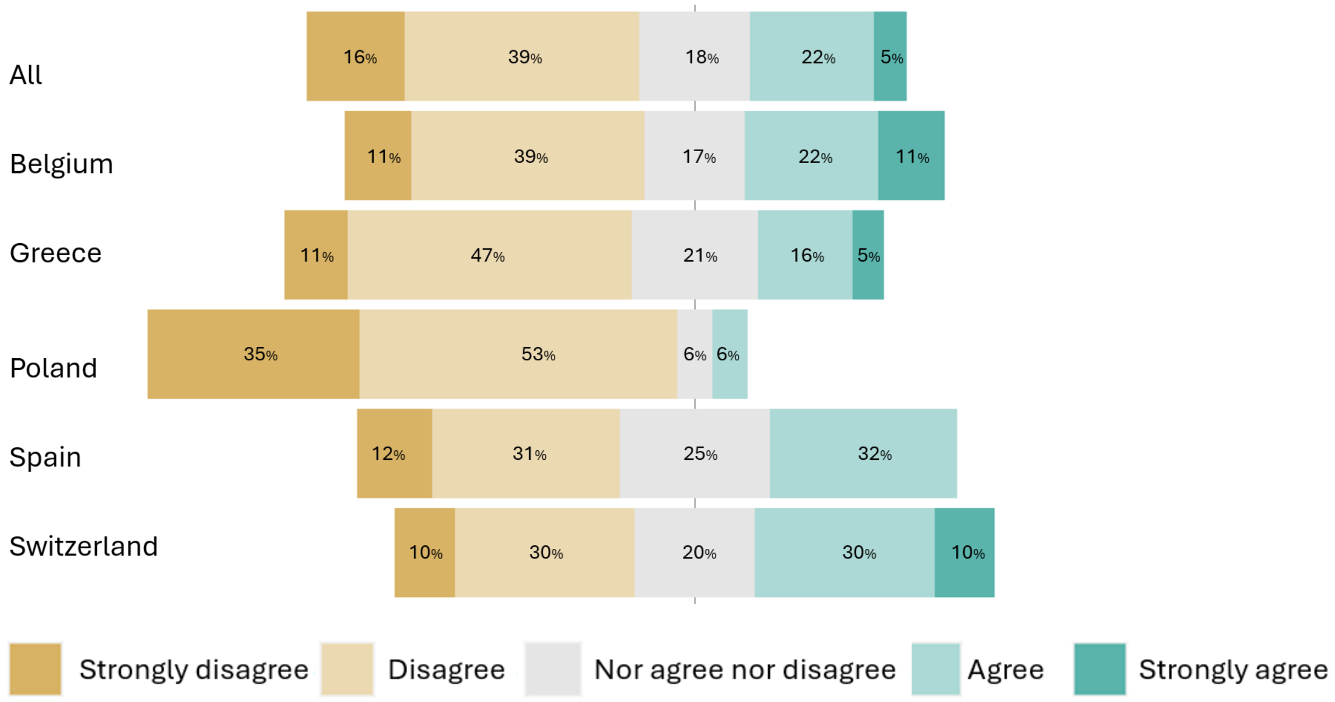

I have been trained to implement alternative approaches for sleep problems in older adults.

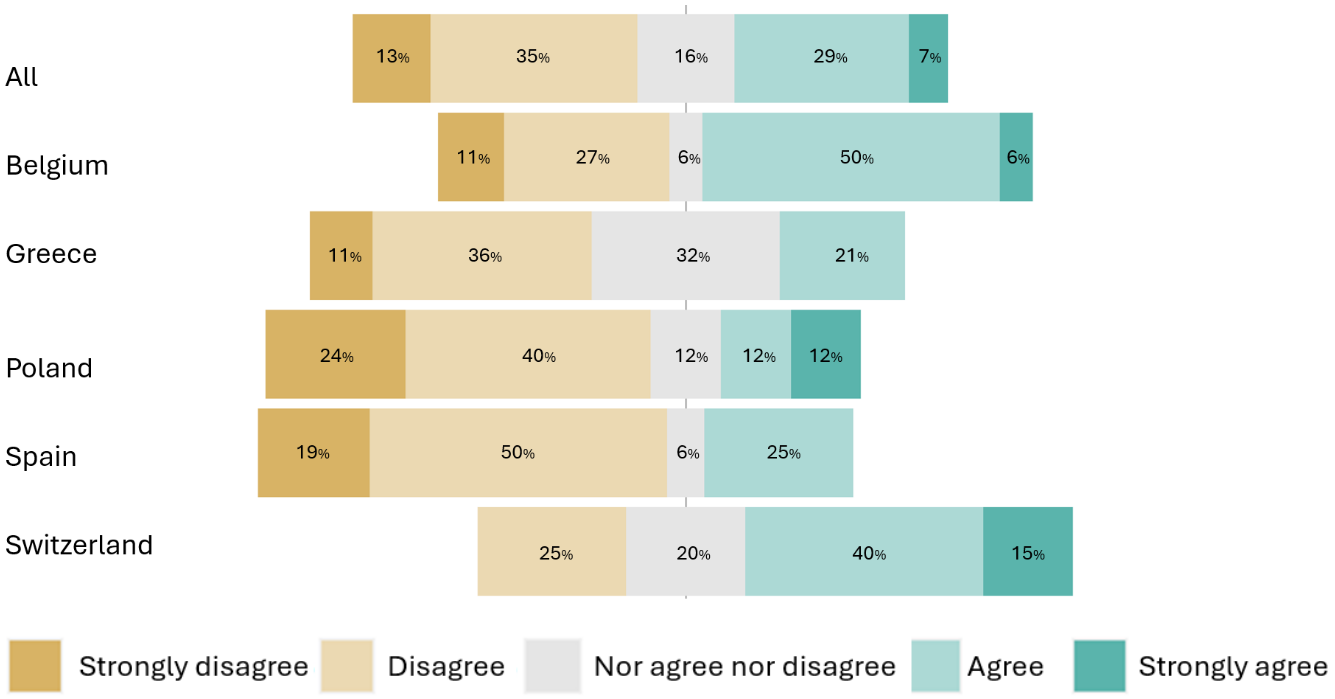

eTable 4.3. Memory, attention, and decision process

TDF-based items	Mean (SD)				
	Belgium	Greece	Poland	Spain	Switzerland
I usually do not consider BZRA deprescribing in older adults with sleep problems because it is a difficult and time-consuming process.	3.56* (0.98)	3.32* (1.11)	3.18* (1.13)	3.69* (0.95)	4.05* (1.05)
As long as the patient has no specific issue or request, I renew/continue the prescription of BZRA.	3.32* (0.95)	2.79* (1.13)	3.56* (1.25)	3.00* (0.89)	3.70* (0.87)

* reversed mean applied to negative statements (so that for all items, the lower the mean, the higher the barrier)

I usually do not consider deprescribing BZRA in older adults as it is a difficult and time-consuming process.

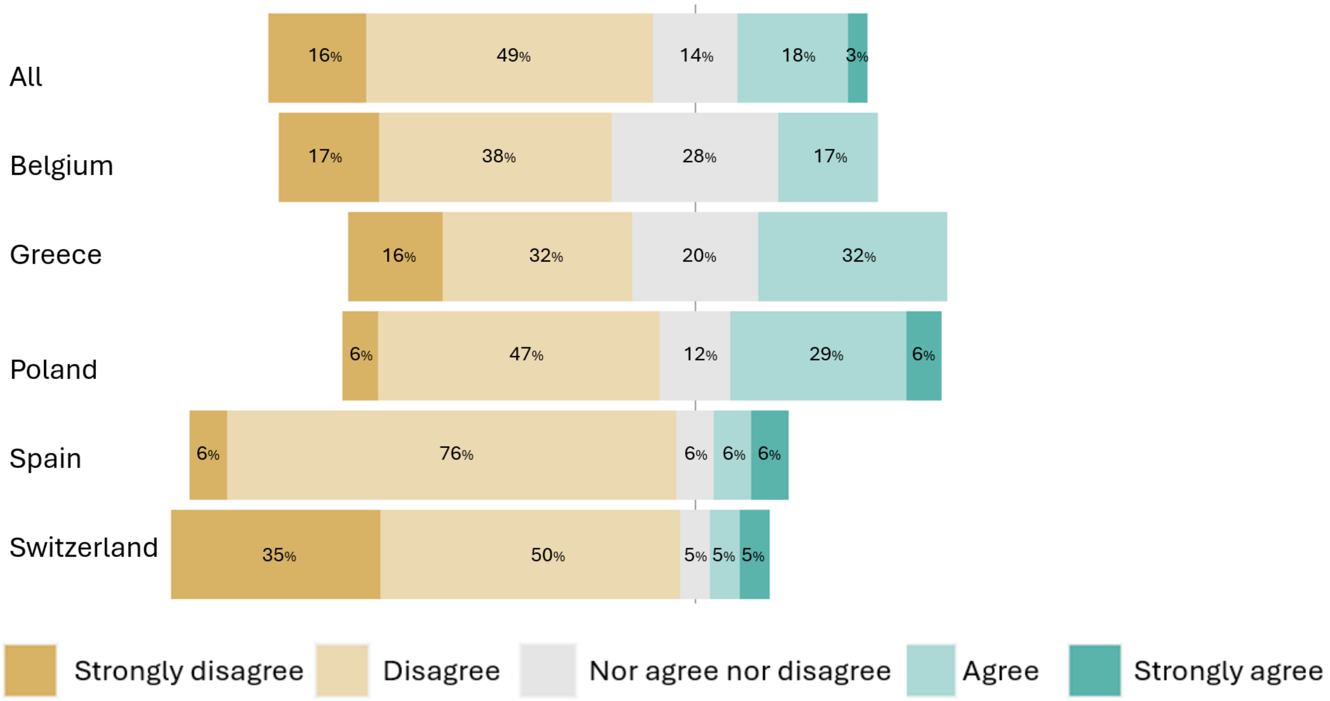

As long as the patient has no specific issue or request, I renew/continue the prescription of BZRA.

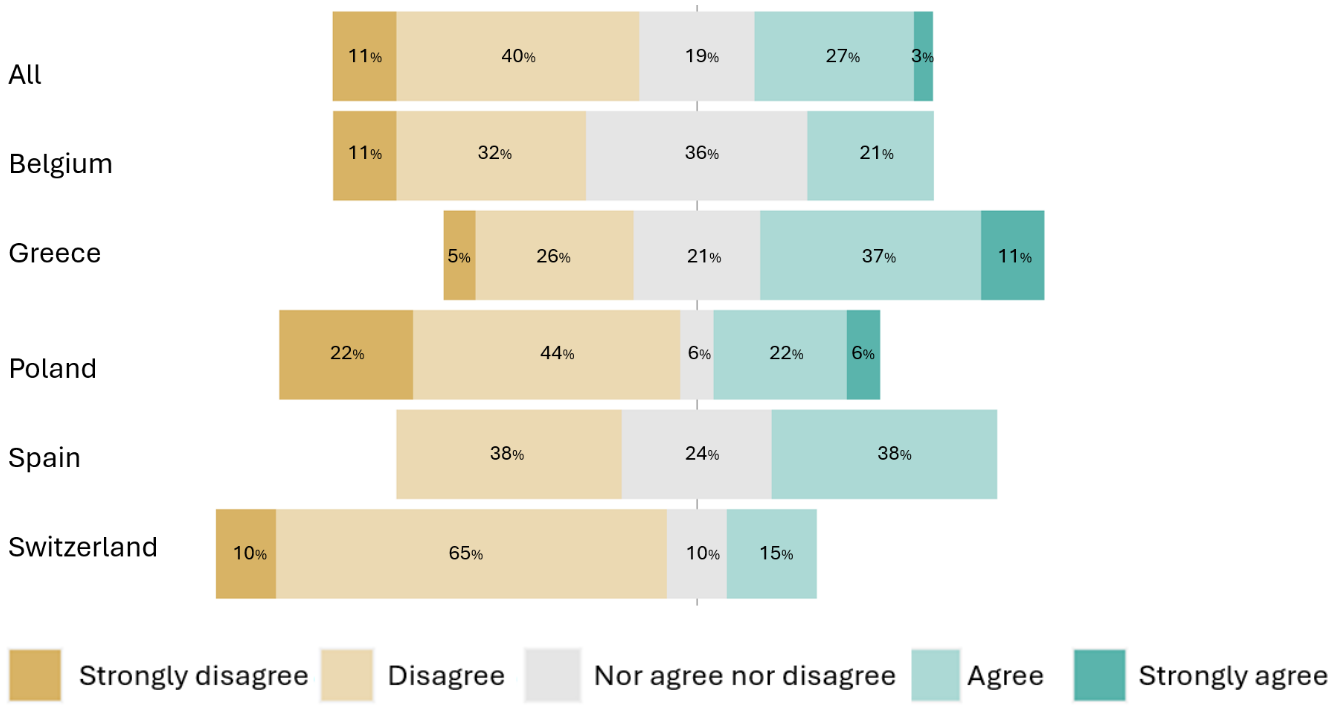

eTable 4.4. Social/Professional Role and Identity

TDF-based items	Mean (SD)				
	Belgium	Greece	Poland	Spain	Switzerland
It is my responsibility as a general practitioner to deprescribe BZRA in older adults with sleep problems.	4.25 (1.07)	3.32 (0.75)	4.11 (0.99)	4.06 (0.99)	4.40 (0.68)
In my practice, it is relevant to follow up on BZRA deprescribing initiated by a physician from the hospital.	4.33 (0.84)	3.58 (1.12)	3.65 (0.86)	4.19 (1.05)	4.50 (0.69)
I don't feel concerned with the deprescribing of BZRA in older adults.	4.63* (0.49)	3.53* (0.91)	3.69* (0.95)	3.94* (1.39)	4.70* (0.47)

* reversed mean applied to negative statements (so that for all items, the lower the mean, the higher the barrier)

It is my responsibility as a general practitioner to deprescribe BZRA in older adults with sleep problems.

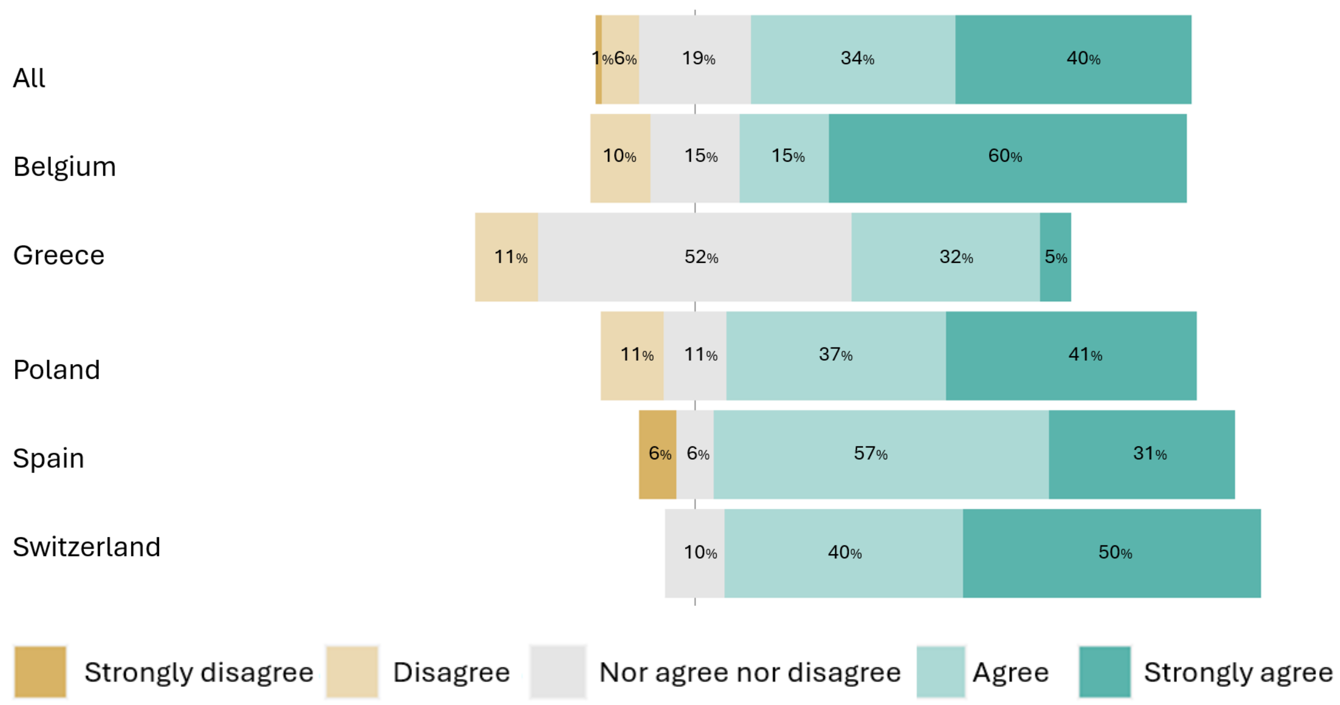

In my practice, it is relevant to follow up on BZRA deprescribing initiated by a hospital physician.

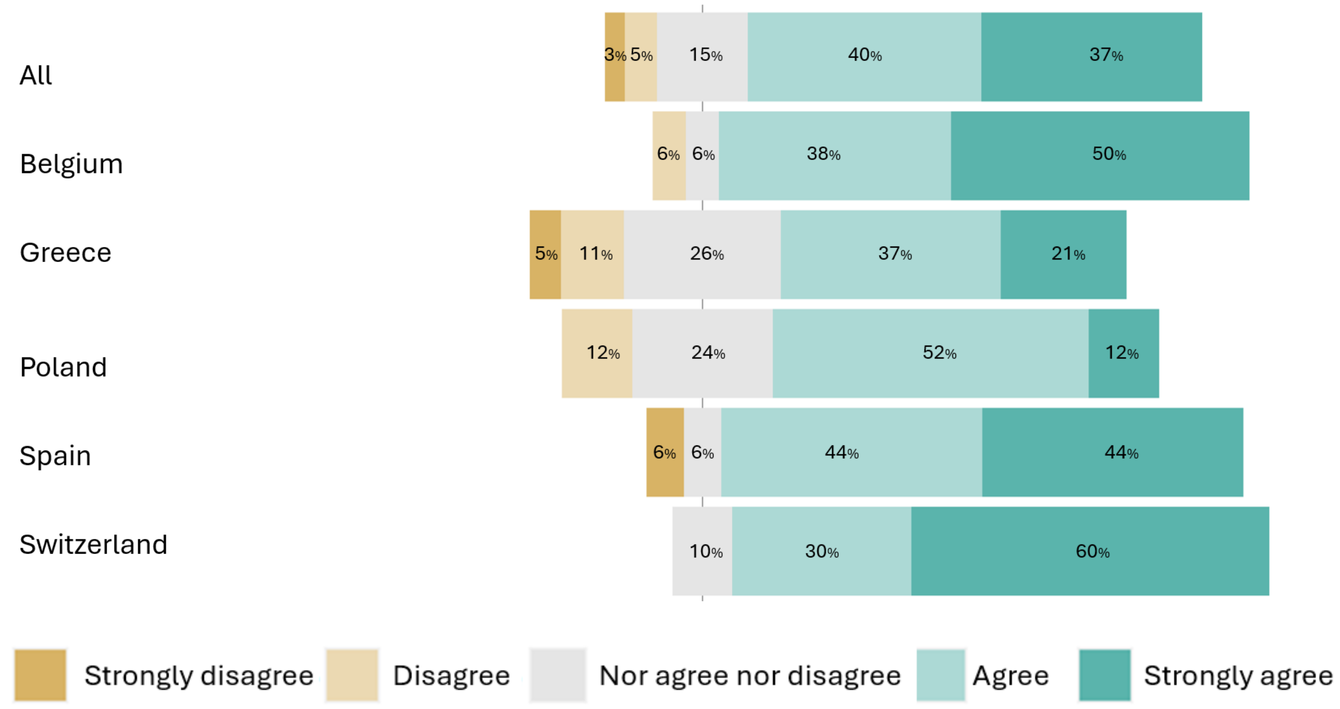

I don't feel concerned with the deprescribing of BZRA in older adults.

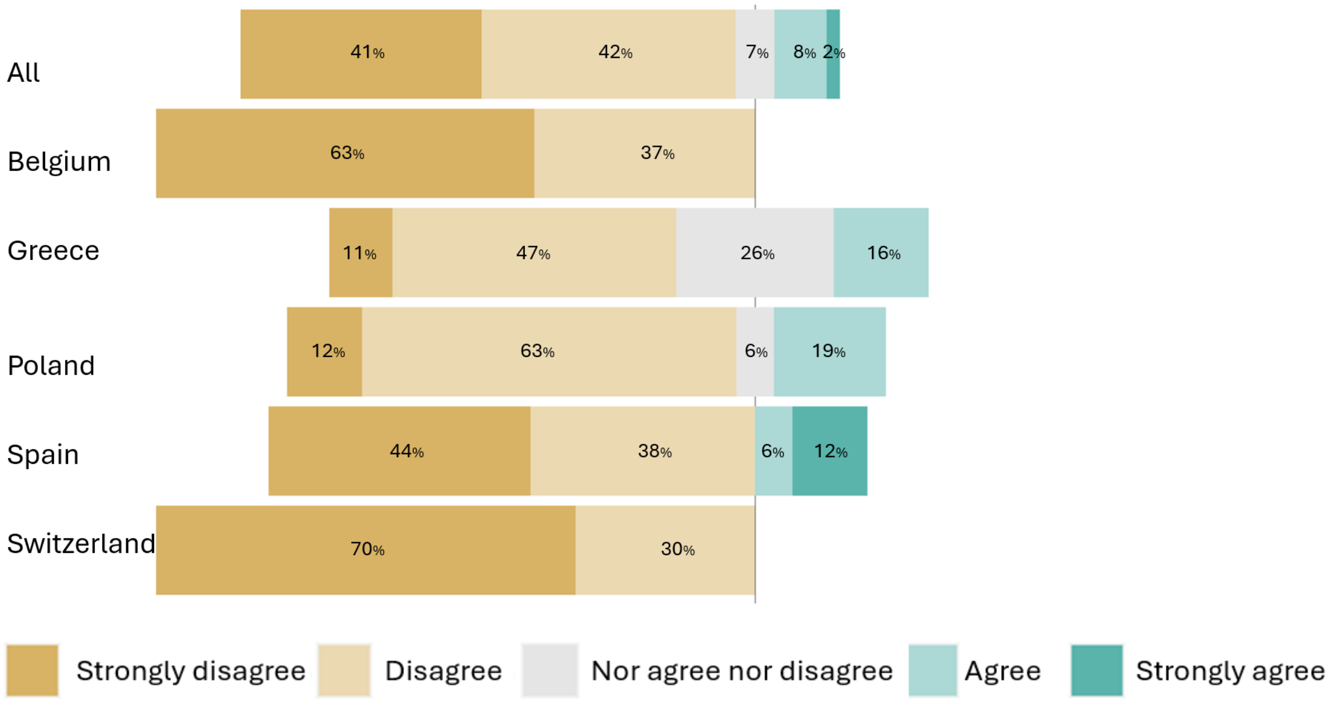

eTable 4.5. Beliefs about capabilities

TDF-based items	Mean (SD)				
	Belgium	Greece	Poland	Spain	Switzerland
I am confident that I can deprescribe BZRA in older adults with sleep problems even when I have limited time.	2.89 (1.13)	2.26 (0.81)	2.29 (0.85)	3.00 (0.97)	3.20 (0.89)
I am confident I could deprescribe BZRA in older adults with sleep problems if I wanted to.	3.11 (1.08)	2.63 (0.89)	2.81 (0.83)	3.50 (0.97)	3.45 (0.76)

I am confident that I can deprescribe BZRA in older adults even when I have limited time.

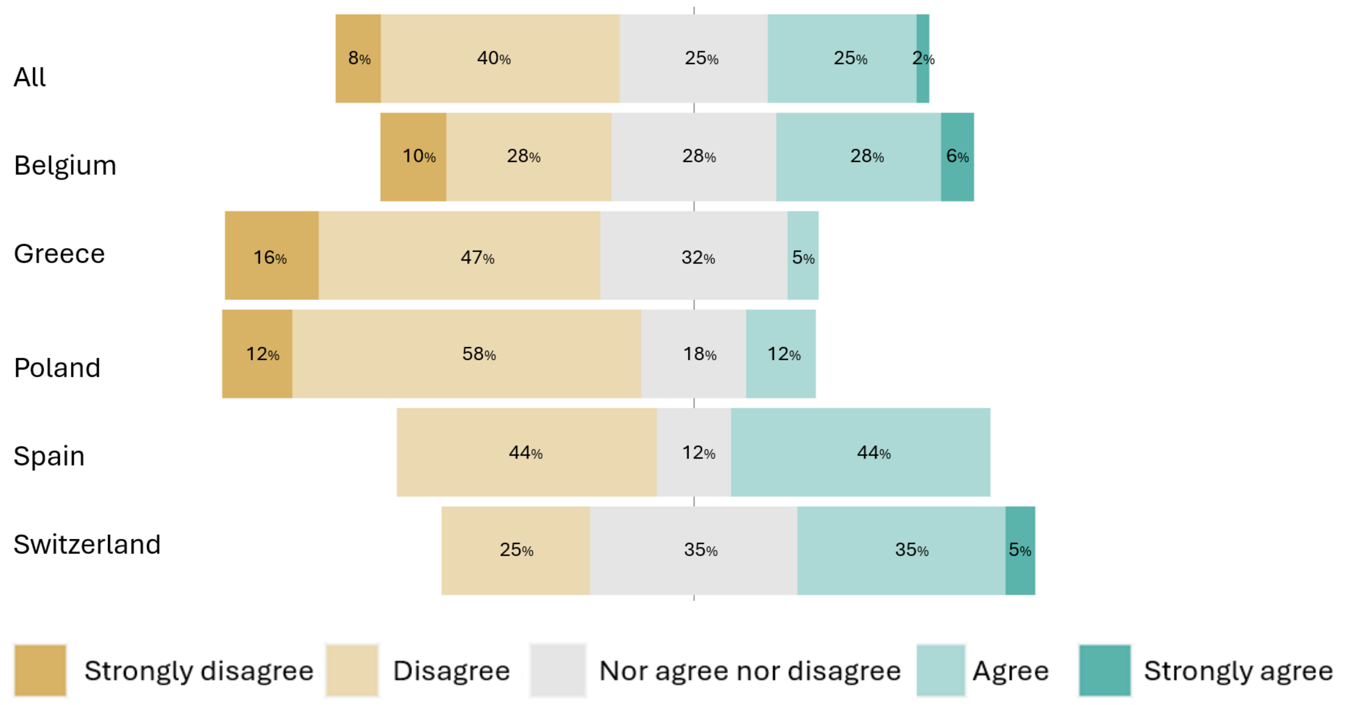

I am confident I could deprescribe BZRA in older adults with sleep problems if I wanted to.

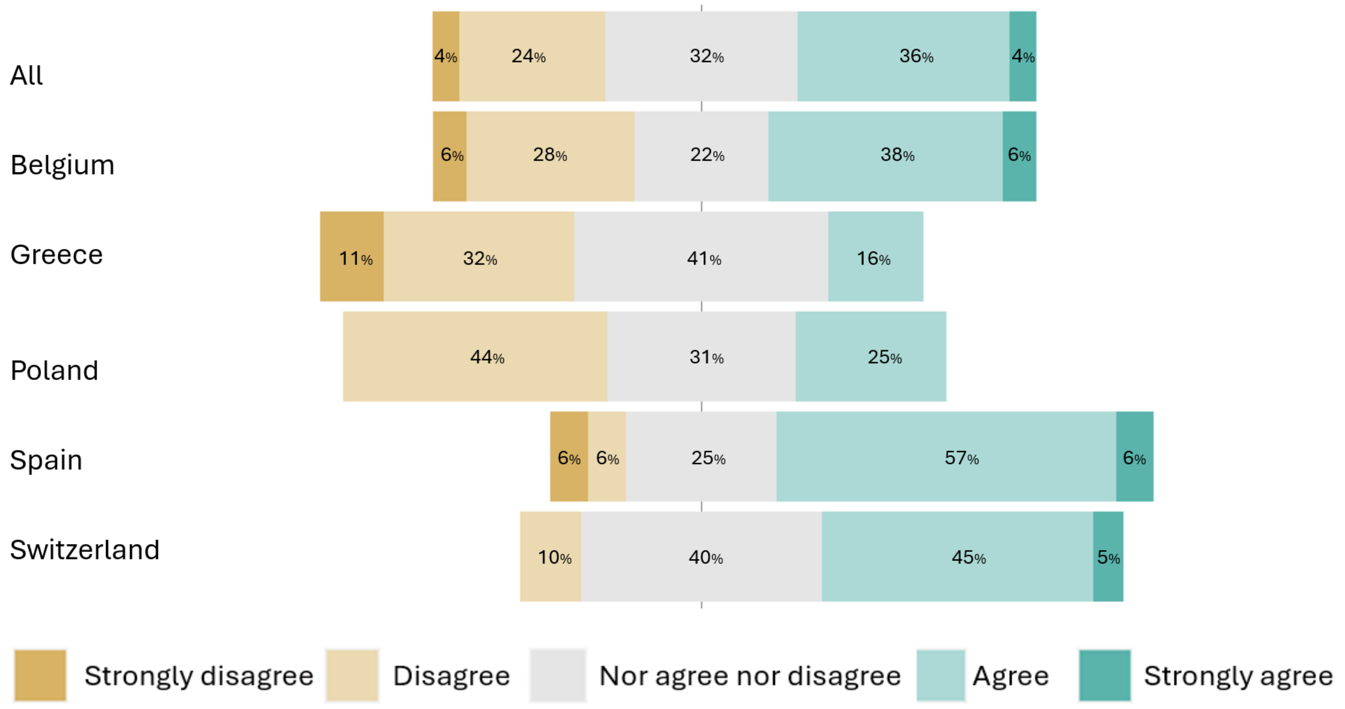

eTable 4.6. Beliefs about consequences

TDF-based items	Mean (SD)				
	Belgium	Greece	Poland	Spain	Switzerland
If I deprescribe BZRA to older adults with sleep problems, it will benefit the population's health in general.	4.17 (0.62)	3.74 (0.81)	4.06 (0.94)	3.56 (1.21)	3.95 (0.83)
If I deprescribe BZRA in older adults with sleep problems, it will negatively affect my relationship with these patients.	3.44* (0.86)	3.42* (0.96)	2.89* (0.83)	3.44* (1.21)	3.80* (0.77)
I believe that BZRA deprescribing in older adults with sleep problems will have negative consequences for my patient's health.	3.39* (0.92)	2.79* (0.78)	3.35* (1.11)	3.56* (1.46)	3.95* (0.61)
In general, I believe that the benefits of BZRA deprescribing in older adults with sleep problems outweigh the harms.	3.78 (1.17)	3.79 (0.92)	3.81 (1.17)	3.81 (0.98)	4.40 (0.50)

* reversed mean applied to negative statements (so that for all items, the lower the mean, the higher the barrier)

If I deprescribe BZRA to older adults, it will benefit the population's health in general.

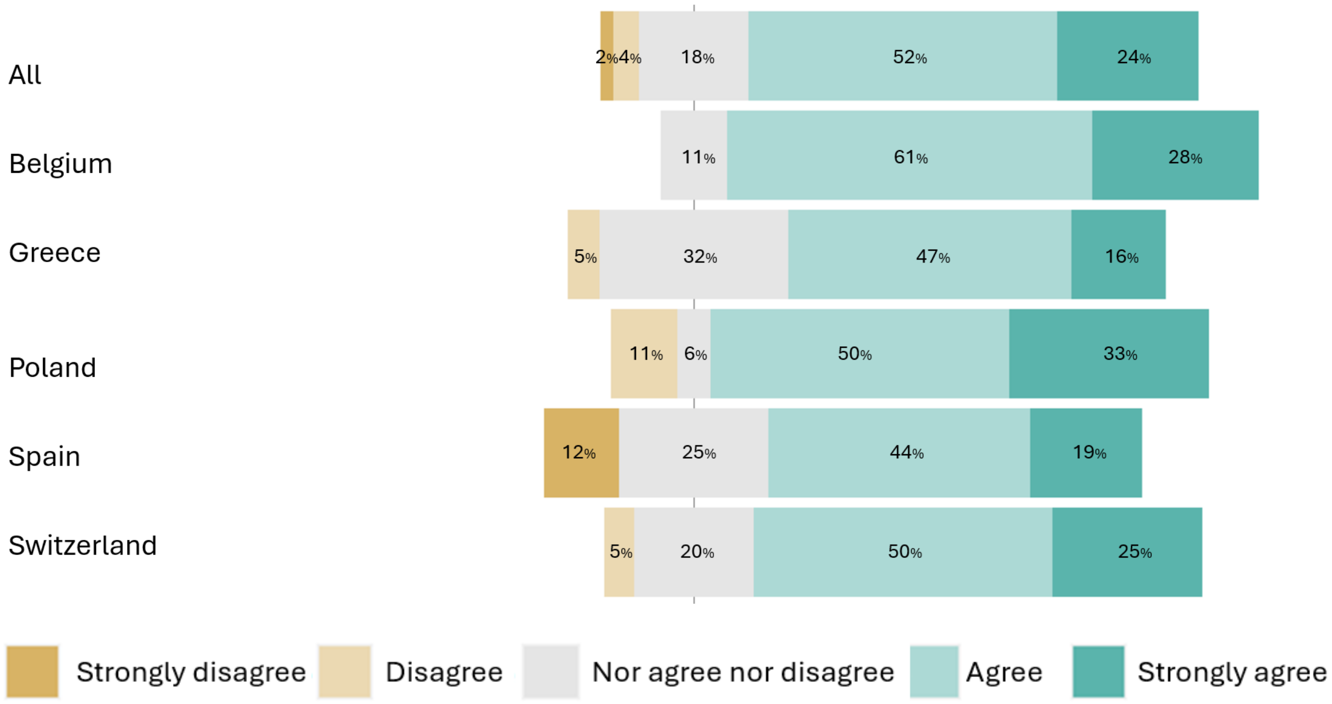

If I deprescribe BZRA in older adults, it will negatively affect my relationship with these patients.

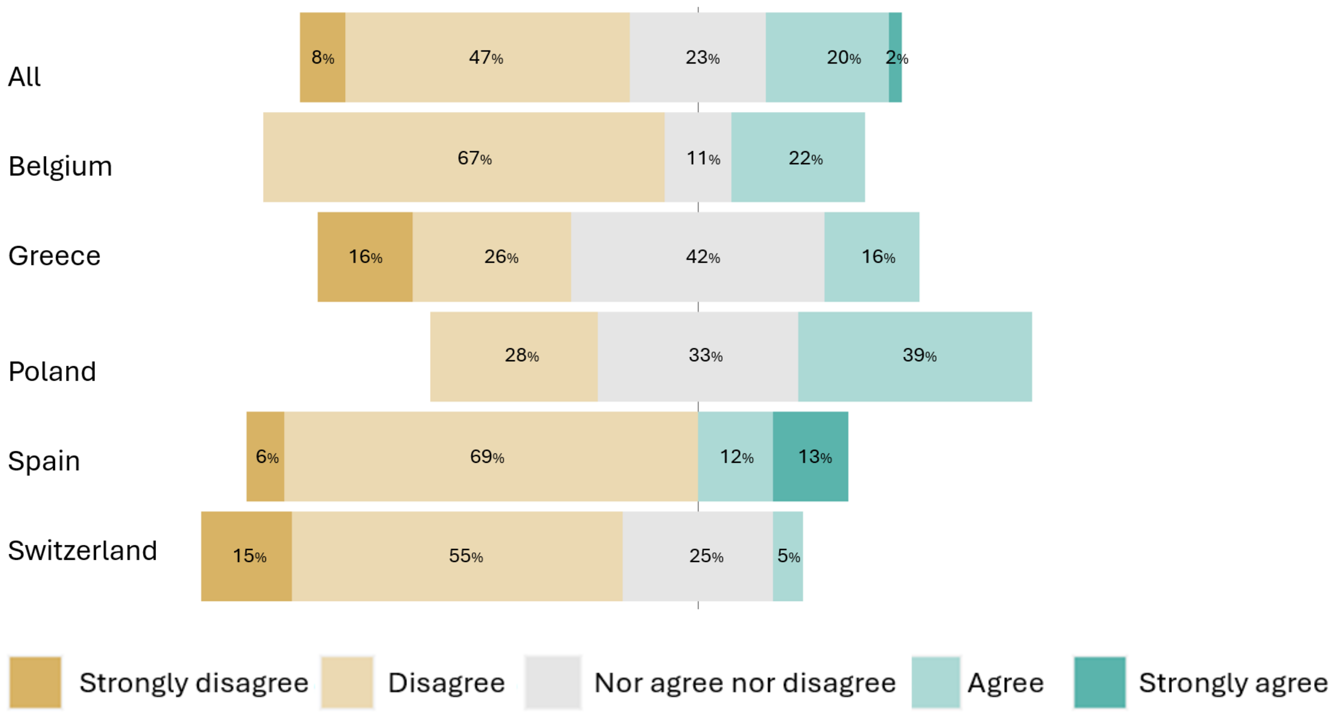

I believe BZRA deprescribing in older adults will have negative consequences for my patient's health.

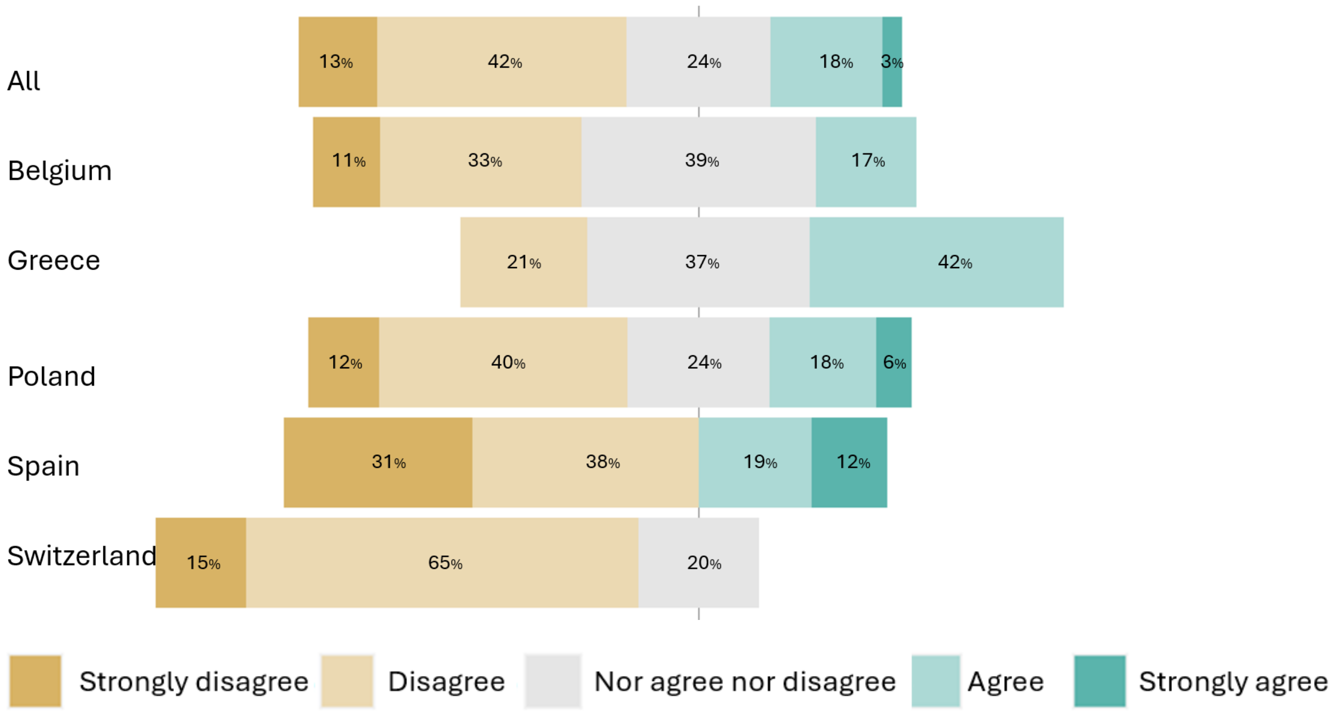

In general, I believe that the benefits of BZRA deprescribing in older adults outweigh the harms.

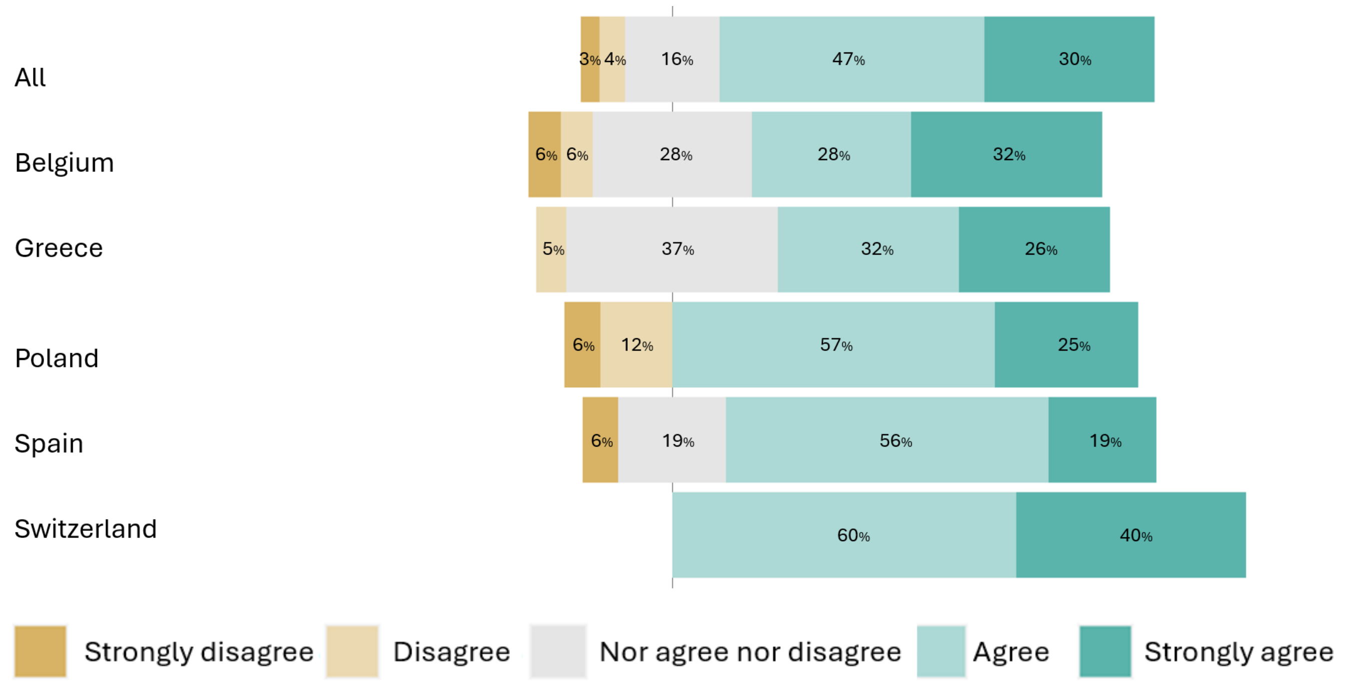

eTable 4.7. Reinforcement

TDF-based items	Mean (SD)				
	Belgium	Greece	Poland	Spain	Switzerland
I am reluctant to deprescribe BZRA in older adults with sleep problems due to previous failed attempts.	2.83 (1.10)	3.37 (0.96)	3.63 (1.09)	2.63 (1.36)	3.40 (0.99)

* reversed mean applied to negative statements (so that for all items, the lower the mean, the higher the barrier)

I am reluctant to deprescribe BZRA in older adults with sleep problems due to previous failed attempts.

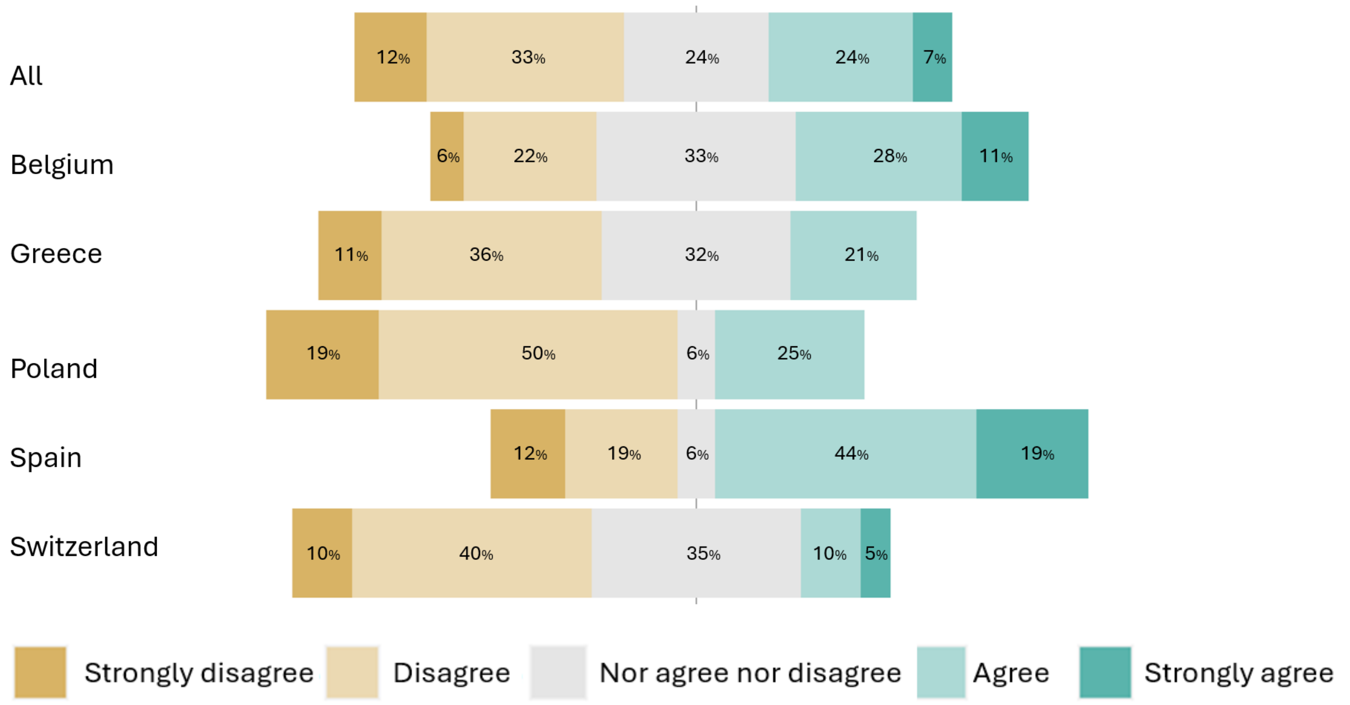

eTable 4.8. Goals

TDF-based items	Mean (SD)				
	Belgium	Greece	Poland	Spain	Switzerland
BZRA deprescribing in older adults with sleep problems is a priority for me.	3.33 (0.97)	2.68 (0.82)	2.76 (0.90)	3.25 (1.13)	3.25 (0.97)
My patients often have other health problems that are usually more important for me to address than the BZRA deprescribing.	3.17* (0.92)	3.63* (0.68)	3.81* (1.05)	3.25* (1.00)	3.40* (0.88)

* reversed mean applied to negative statements (so that for all items, the lower the mean, the higher the barrier)

BZRA deprescribing in older adults with sleep problems is a priority for me.

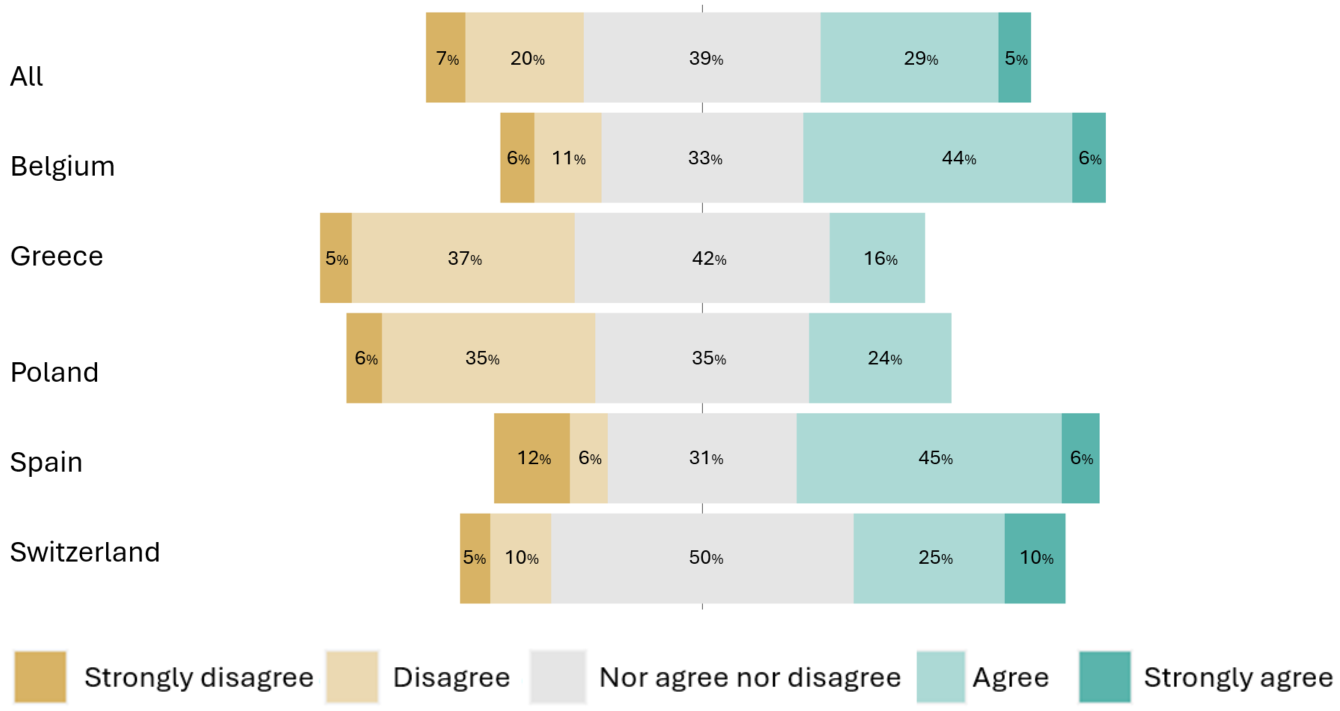

My patients have other health problems that are more important to address than the BZRA deprescribing.

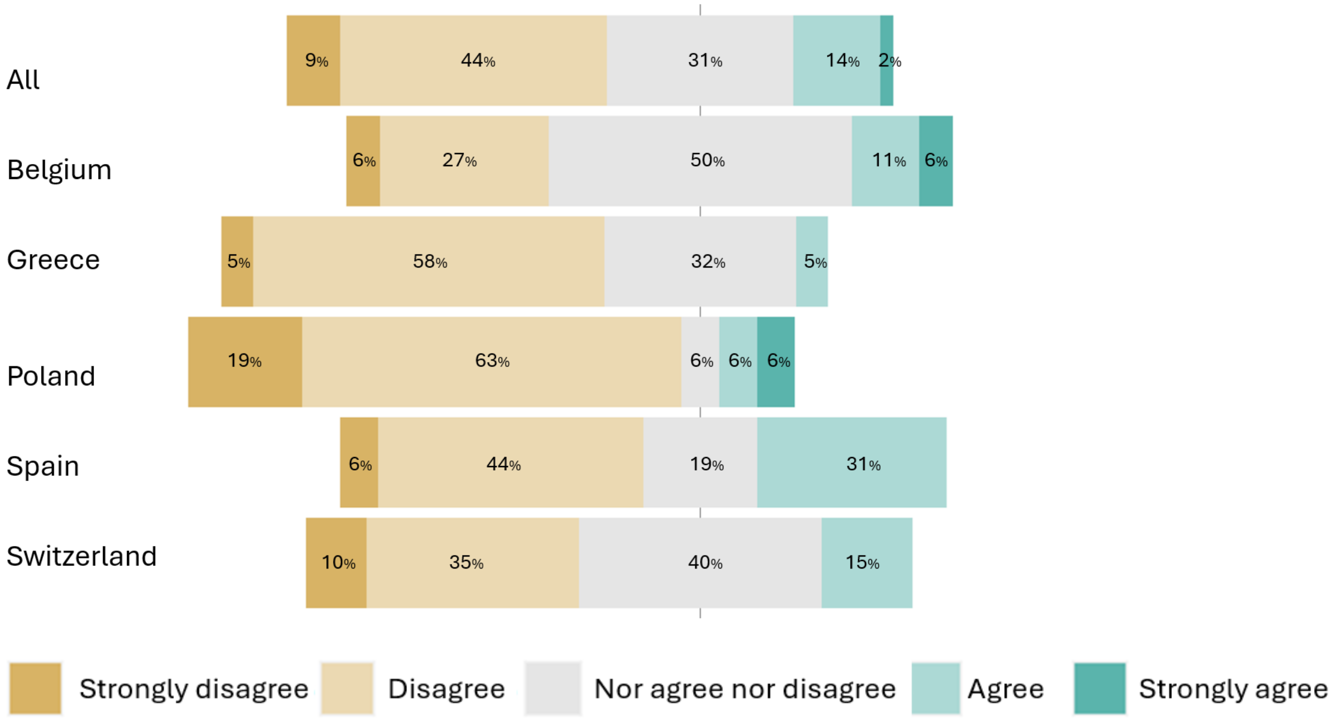

eTable 4.9. Intentions

TDF-based items	Mean (SD)				
	Belgium	Greece	Poland	Spain	Switzerland
I intend to deprescribe BZRA in older adults with sleep problems.	4.00 (0.94)	3.05 (0.78)	4.06 (0.66)	3.81 (0.98)	4.25 (0.55)
I intend to promote the use of alternative approaches to help older adults deal with sleep problems.	4.00 (0.76)	3.53 (0.77)	3.71 (1.16)	3.81 (1.17)	4.25 (0.55)

I intend to deprescribe BZRA in older adults with sleep problems.

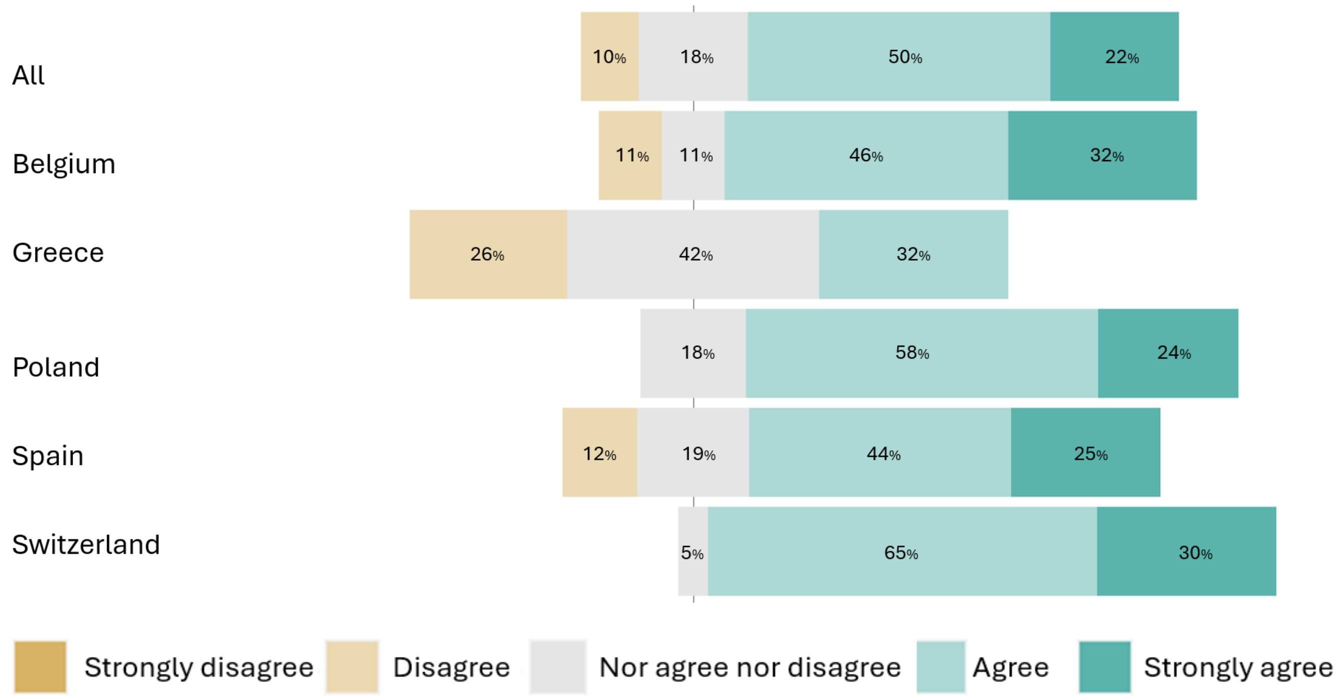

I intend to promote the use of alternative approaches to help older adults deal with sleep problems.

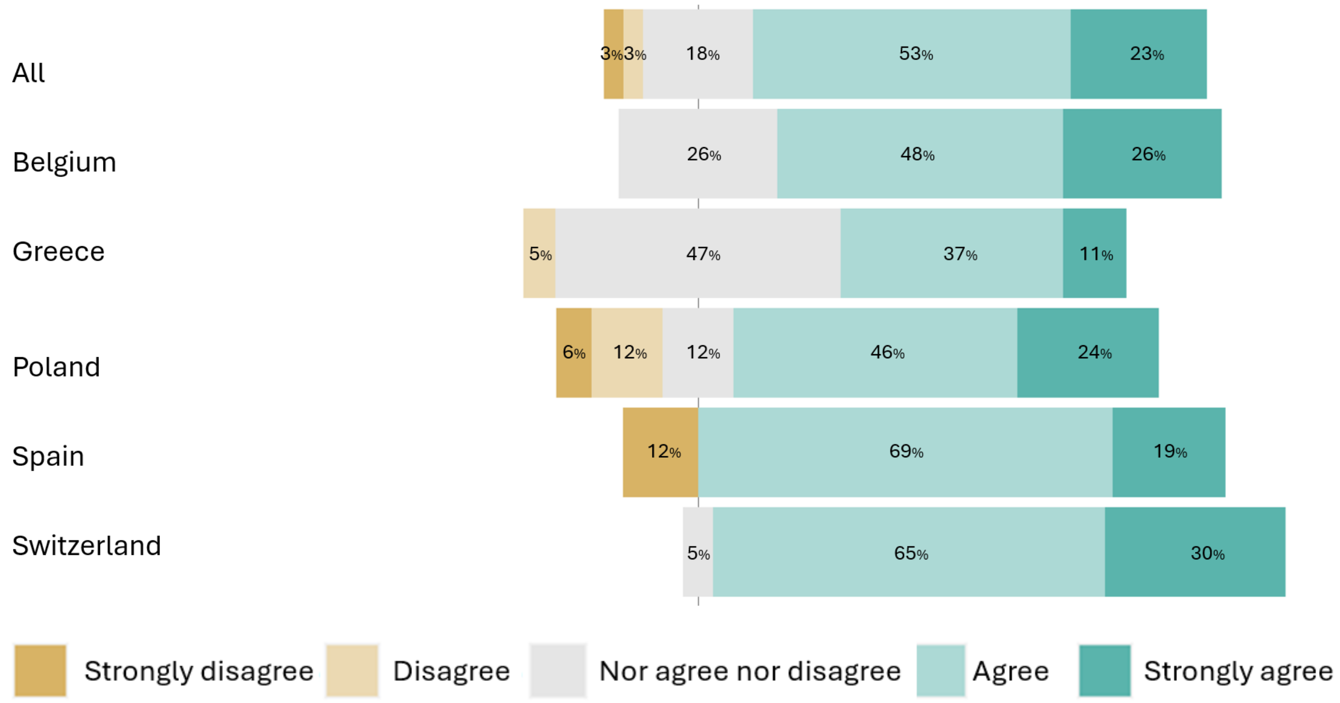

eTable 4.10. Emotions

TDF-based items	Mean (SD)				
	Belgium	Greece	Poland	Spain	Switzerland
I feel frustrated with all the challenges of BZRA deprescribing in older adults with sleep problems.	2.61* (1.09)	2.74* (0.99)	2.25* (0.93)	2.50* (1.03)	2.70* (0.98)
I feel stressed about BZRA deprescribing in older adults with sleep problems.	3.61* (1.20)	2.68* (1.06)	2.29* (0.99)	2.94* (1.24)	3.65* (0.75)

* reversed mean applied to negative statements (so that for all items, the lower the mean, the higher the barrier)

I feel frustrated with all the challenges of BZRA deprescribing in older adults with sleep problems.

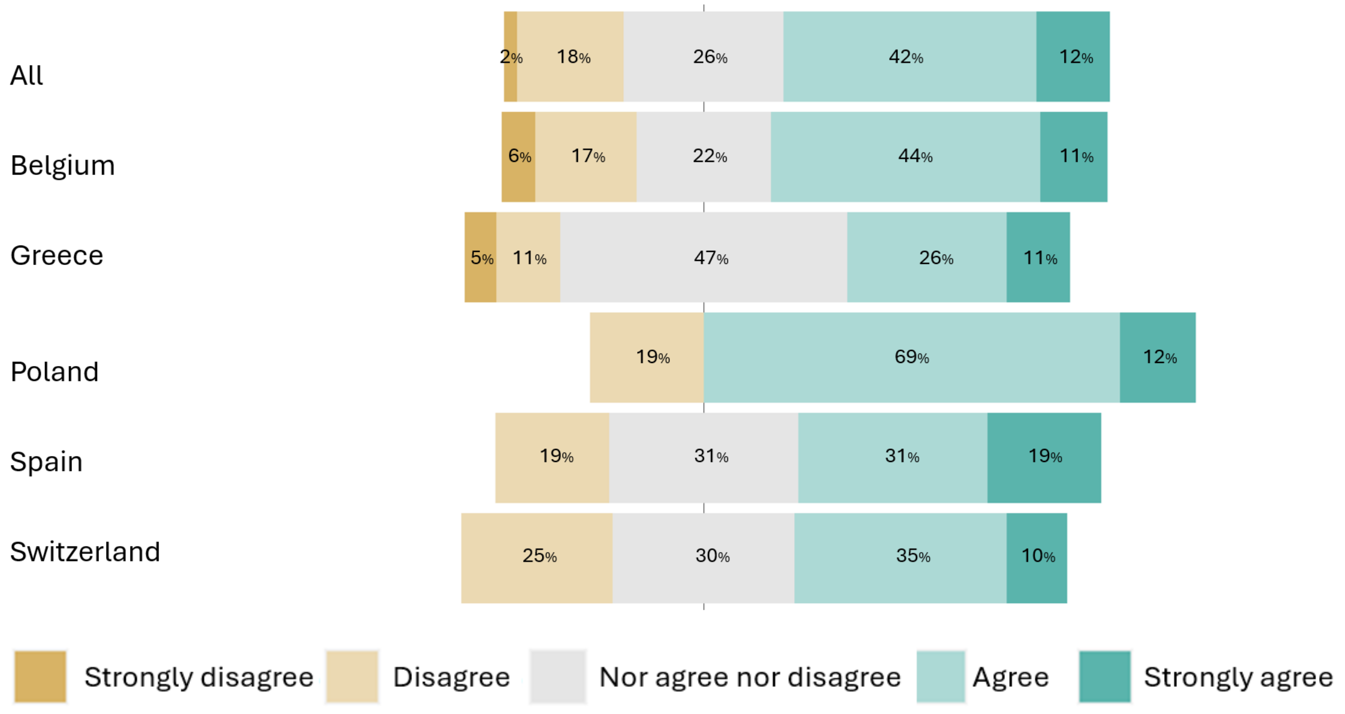

I feel stressed about BZRA deprescribing in older adults with sleep problems.

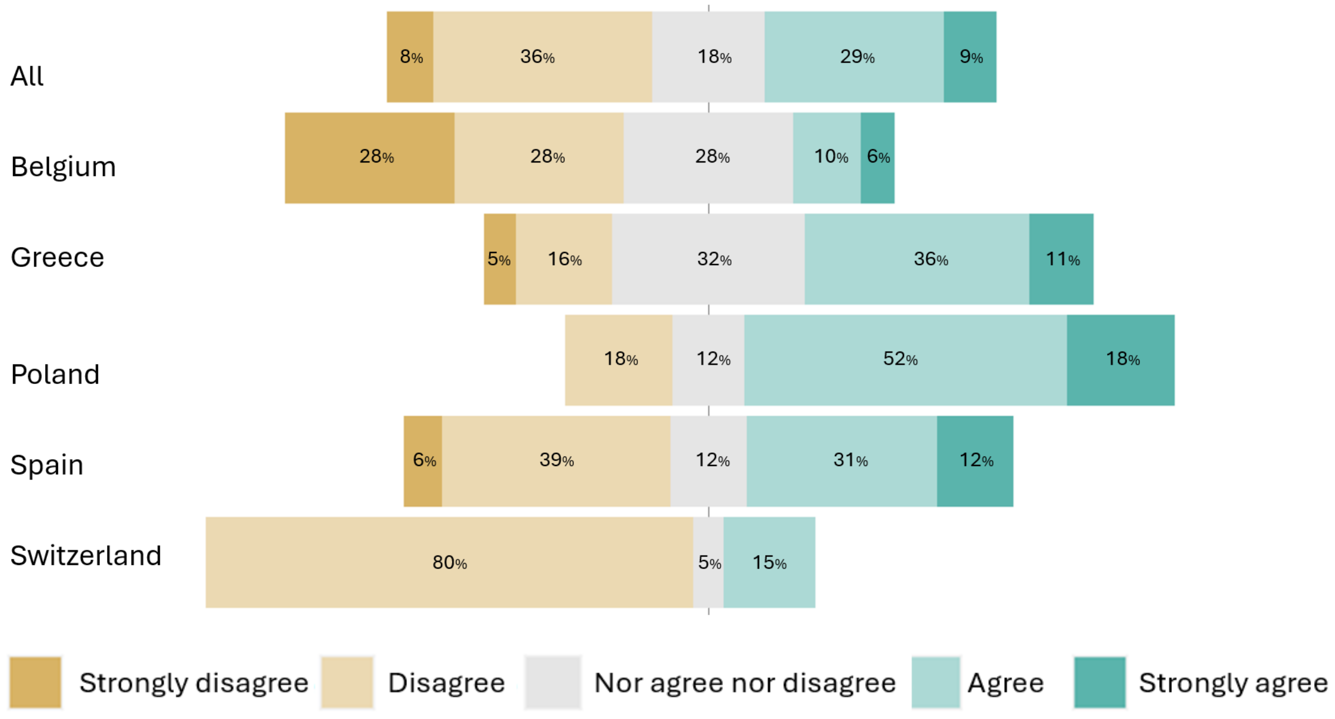

eTable 4.11. Environmental context, and resources

TDF-based items	Mean (SD)				
	Belgium	Greece	Poland	Spain	Switzerland
There are guidelines and tools available for BZRA deprescribing in older adults with sleep problems that are possible to implement in my practice.	3.56 (0.86)	3.42 (0.86)	3.00 (0.94)	3.47 (0.83)	2.75 (0.91)
I have enough time to educate and inform patients about BZRA deprescribing.	2.95 (1.18)	2.79 (1.18)	1.50 (0.52)	2.00 (1.15)	3.00 (1.03)
In my practice, there are enough collaborators to support BZRA deprescribing in older adults with sleep problems.	2.79 (1.03)	2.11 (1.03)	1.50 (0.63)	2.88 (1.26)	2.40 (0.82)
In my opinion, BZRA deprescribing is not prioritized by our healthcare system.	2.56* (1.42)	3.32* (1.42)	3.06* (0.99)	3.44* (1.15)	3.20* (1.20)
For general practitioners, in my area or my region, goals or policies have been set that encourage BZRA deprescribing.	2.30 (1.17)	1.84 (1.17)	1.38 (0.88)	3.63 (1.02)	2.30 (0.66)

* reversed mean applied to negative statements (so that for all items, the lower the mean, the higher the barrier)

There are guidelines/tools available for BZRA deprescribing in older adults implementable in my practice.

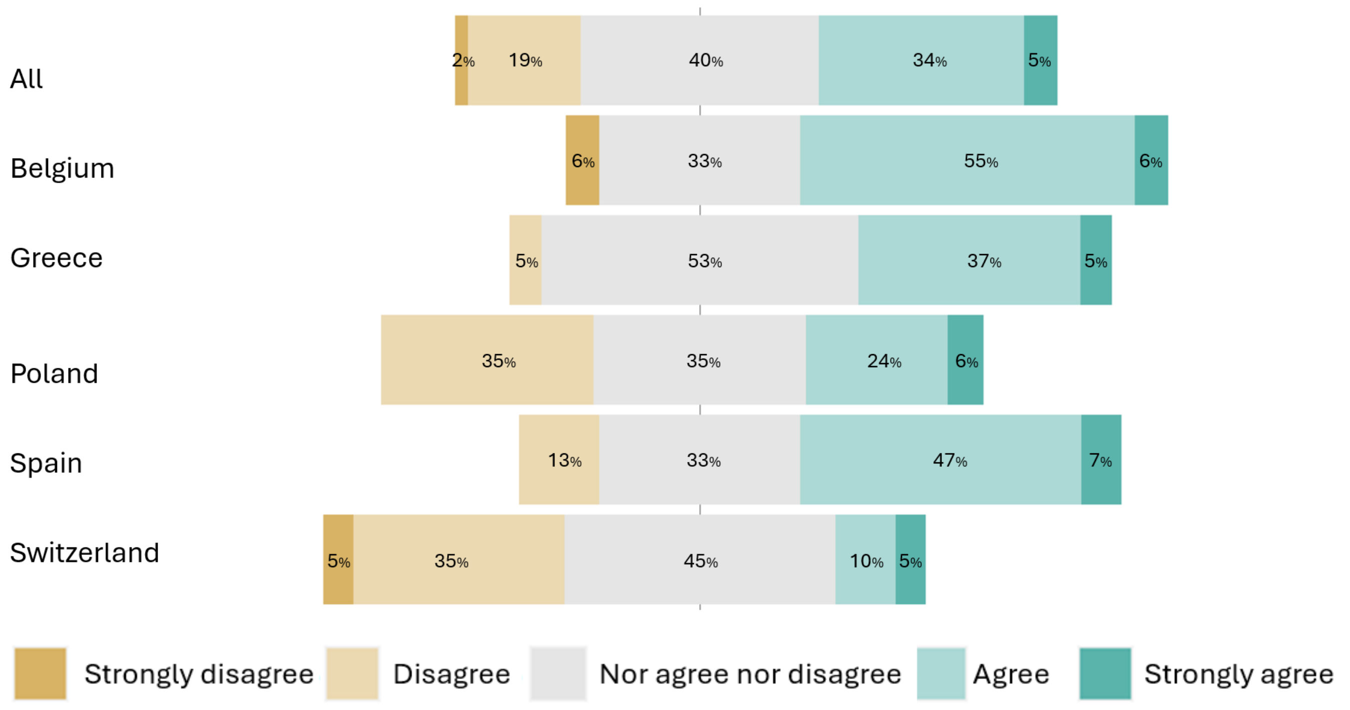

I have enough time to educate and inform patients about BZRA deprescribing.

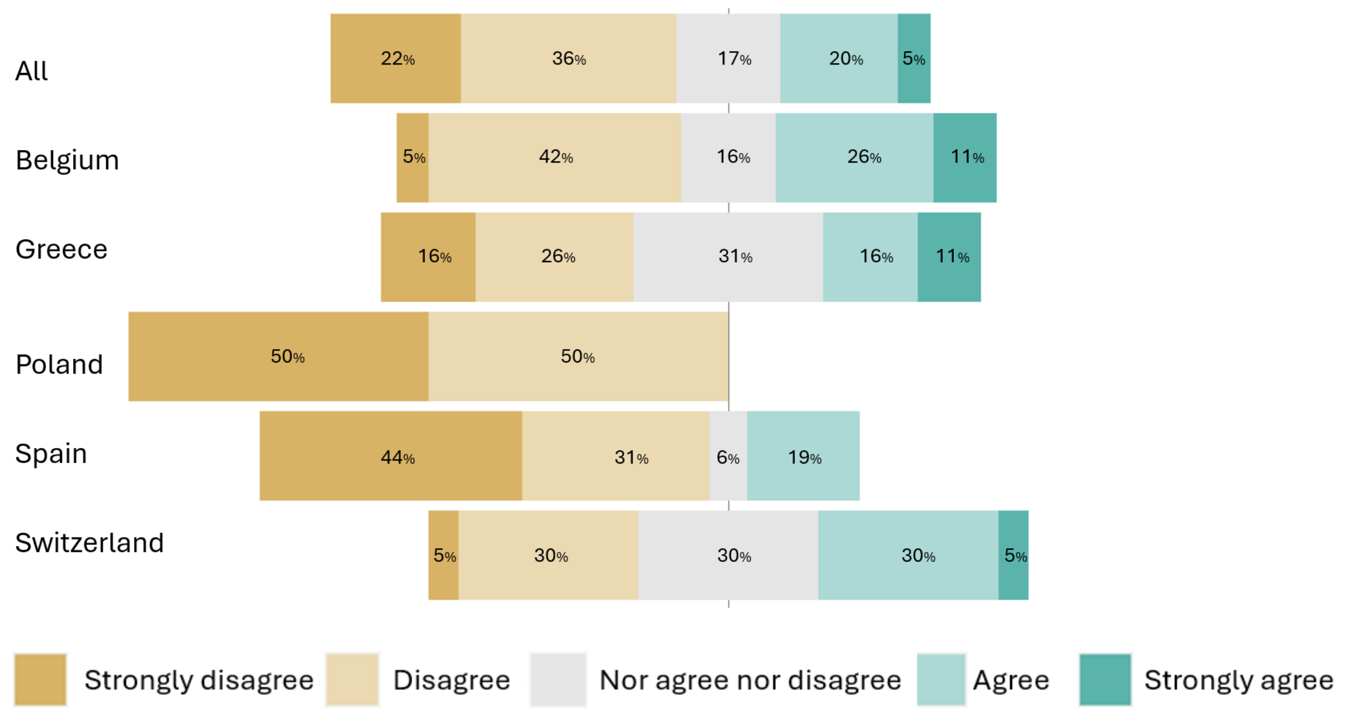

In my practice, there are enough collaborators to support BZRA deprescribing in older adults.

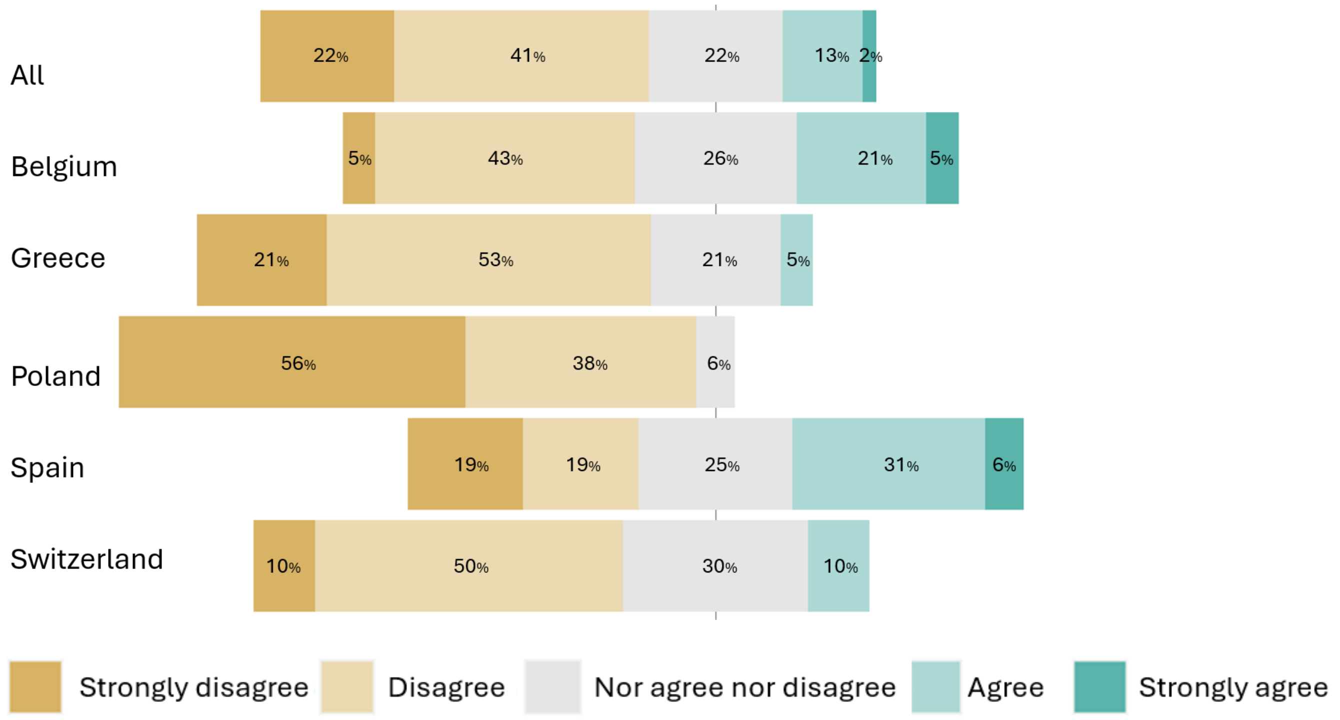

In my opinion, BZRA deprescribing is not prioritized by our healthcare system.

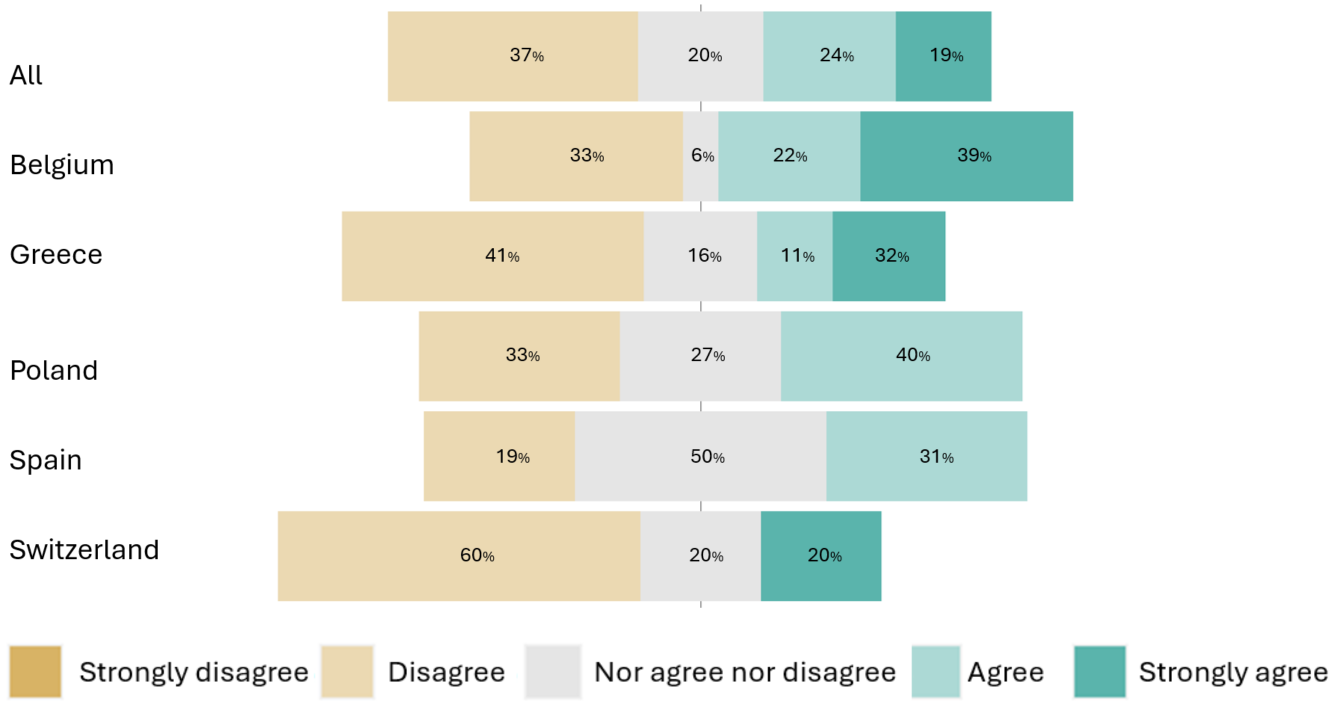

For GPs, in my area or my region, goals or policies have been set that encourage BZRA deprescribing.

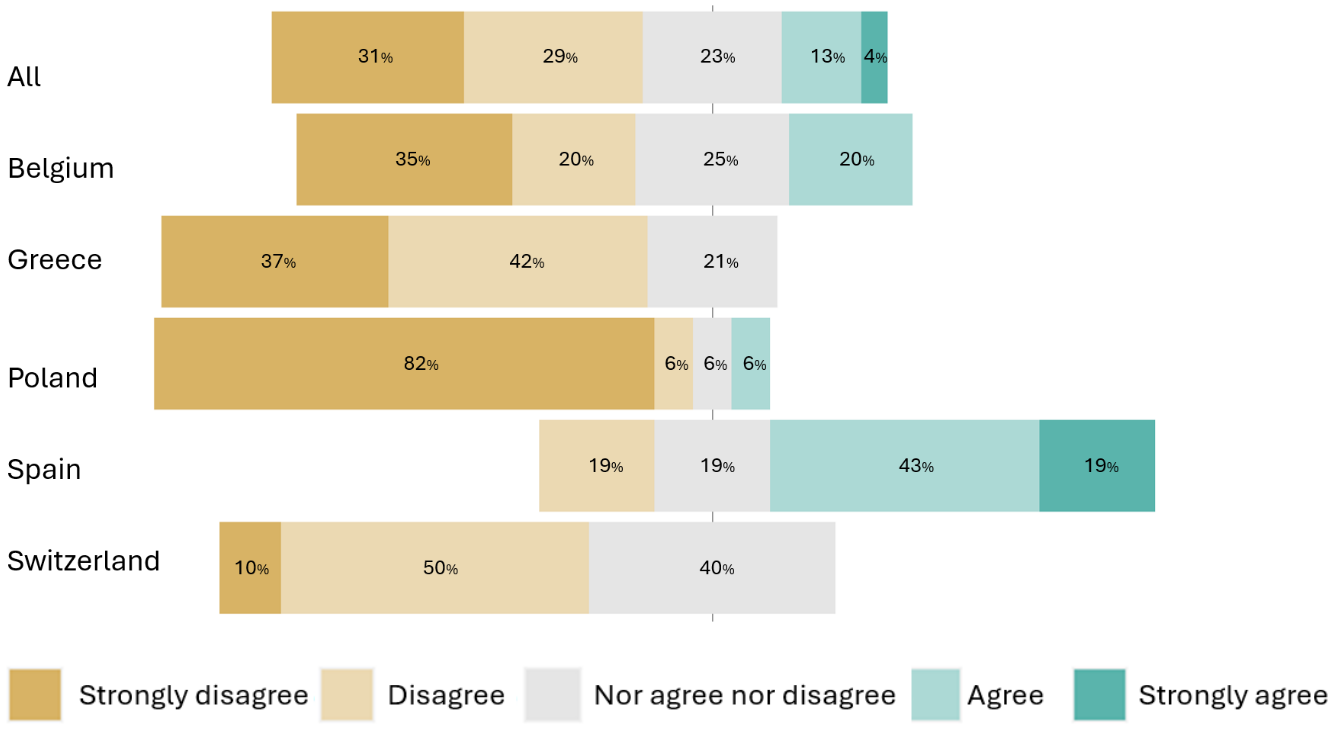

eTable 4.12. Social influence

TDF-based items	Mean (SD)				
	Belgium	Greece	Poland	Spain	Switzerland
I feel a lot of pressure from older adults with sleep problems and/or their relatives to renew/extend their prescriptions.	1.60* (0.75)	1.84* (0.60)	1.39* (0.50)	1.39* (0.50)	1.75* (0.77)
Most of my older patients take a BZRA for sleep problems or their relatives are reluctant to deprescribe their BZRA.	1.68* (0.58)	2.00* (0.67)	1.24* (0.44)	1.24* (0.44)	1.60* (0.59)
My colleagues/collaborators whose opinions I value support BZRA deprescribing in older adults with sleep problems.	4.00 (0.75)	2.68 (1.06)	3.41 (0.94)	3.41 (0.94)	3.75 (0.55)
My colleagues are supportive of BZRA deprescribing.	3.89 (0.68)	2.95 (1.03)	3.24 (1.15)	3.24 (1.15)	3.95 (0.61)

* reversed mean applied to negative statements (so that for all items, the lower the mean, the higher the barrier)

I feel a lot of pressure from older adults and/or their relatives to renew/extend their prescriptions.

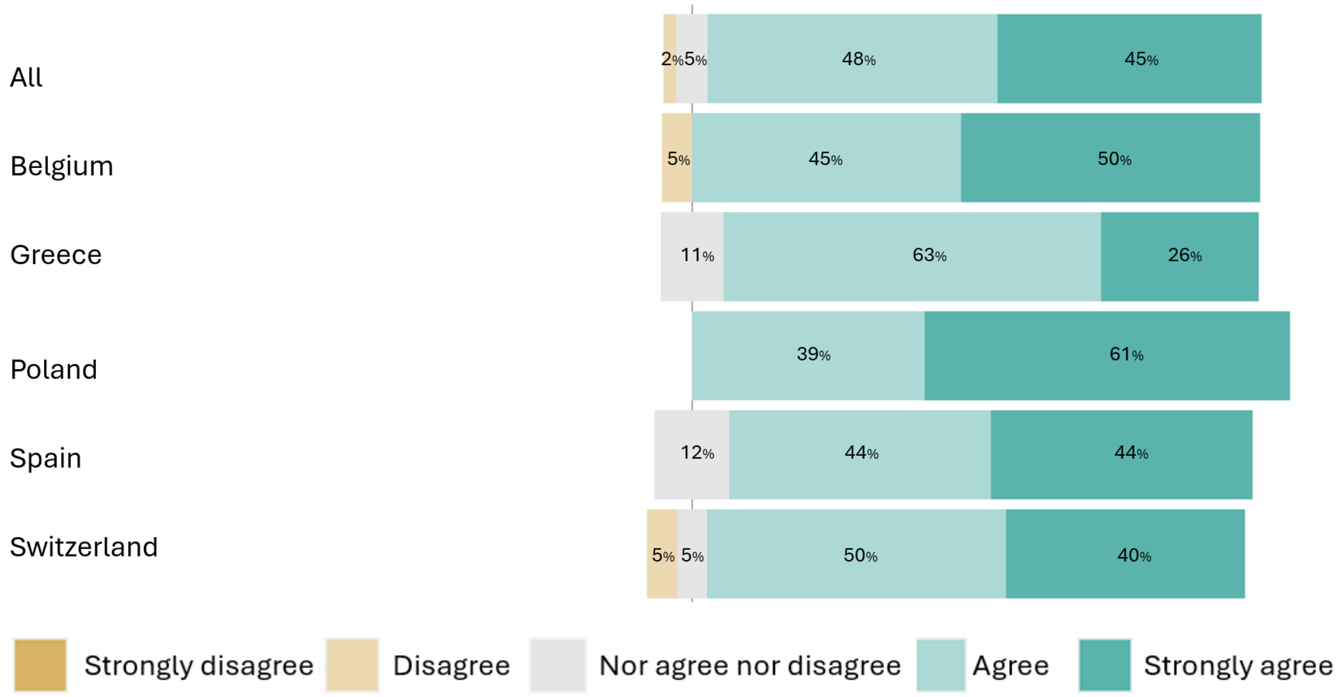

Most of my older patients who take a BZRA, or their relatives, are reluctant to deprescribe their BZRA.

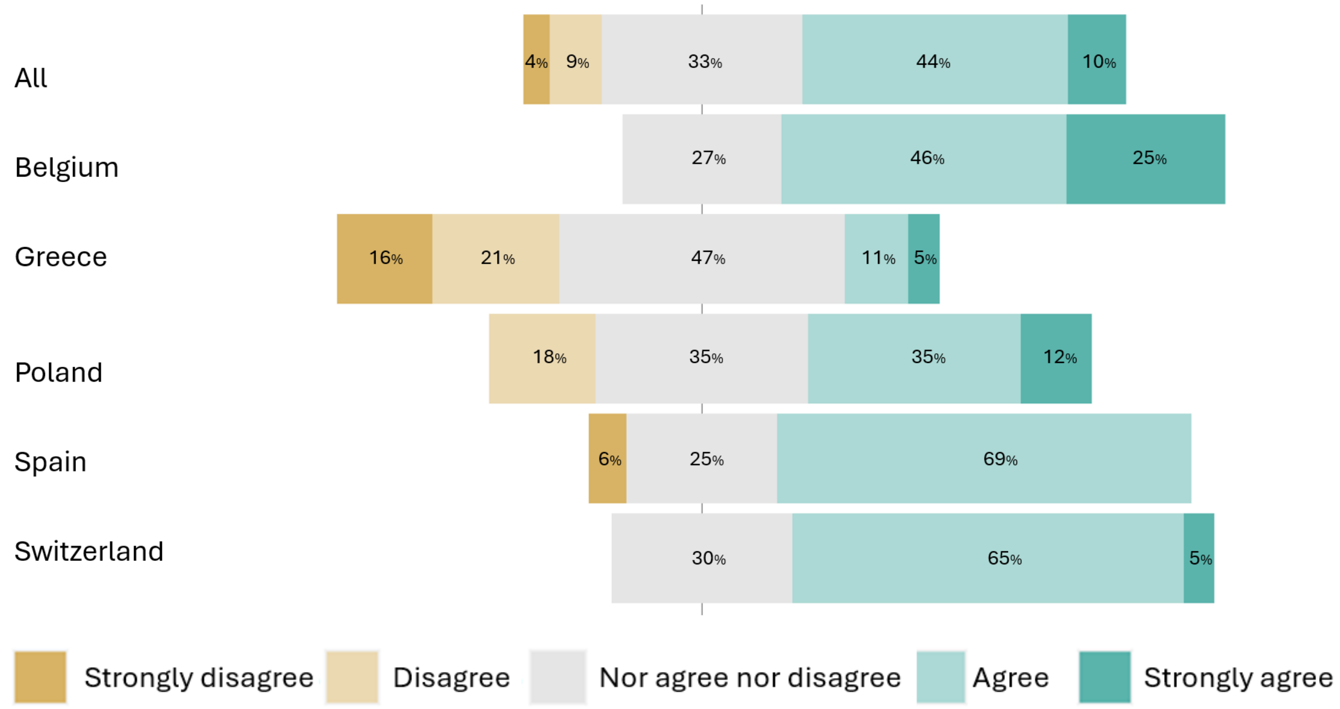

My colleagues/collaborators whose opinions I value support BZRA deprescribing in older adults.

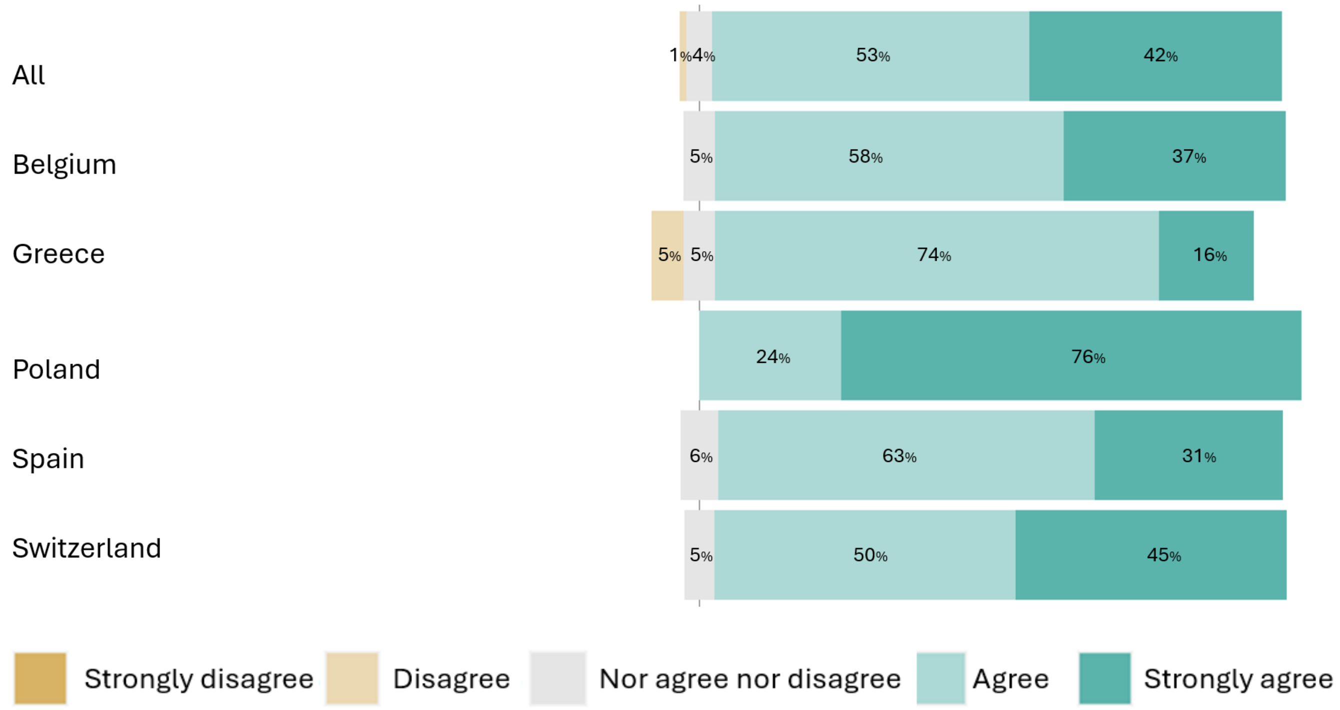

My colleagues are supportive of BZRA deprescribing.

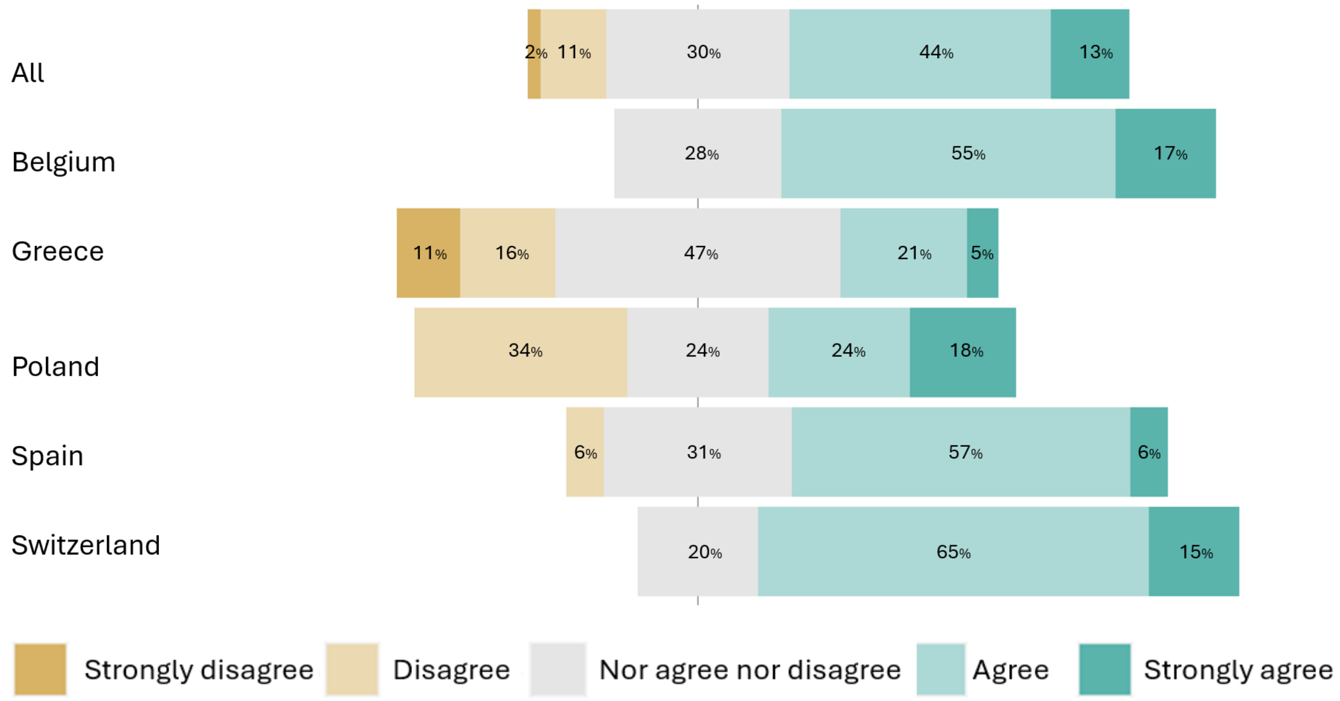

eAppendix 2. Summary of answers to open questions on barriers of and enablers to BZRA deprescribing (general practitioners.)

In collaboration with partners, the statements were translated from the local language into English and deductively coded according to the Theoretical Domains Framework.

Regarding GPs, "Social influence" was a major TDF domain across all 6 countries.

- *"Usually, the family does not agree with stopping BZRA, and often nor do the pharmacists."*
- *"Pressure from families and nursing home staff"*
- *"The patients' belief that these drugs are safe."*
- *"Patients are not motivated at all and do not see the point of the intervention. They often then take medication from relatives/neighbours/friends if I do not prescribe it."*
- *"Non-compliance of patients. Availability of prescription by other colleagues. "*
- *"Treatment with BZRA is started too "easily" from emergency services or hospital specialists, especially during admissions to acute hospitals. This makes its withdrawal difficult, although it does not prevent it outright, because "the specialist has prescribed it, he says it will not harm me and that it is for good".*

GPs also highlighted barriers in the domain of "Environmental context and resources."

- *"There are no effective alternatives."*
- *"Most important: very long appointments to psychiatrist and psychologist, for older patients almost unattainable to reach a specialist in 2 months, no clinics for the treatment of sleep disorders. Costs: sleep medications are cheaper,"*
- *"In a village or a small town, it's hard to find an alternative treatment."*

Enablers are complementary to barriers. GPs stressed that better communication, better access to alternatives, and patient education could help to reduce barriers.

- *"Propositions of feasible alternatives"*
- *"Cooperation and good communication with the patient and with a medical specialist."*
- *"Patients' tendency to prefer alternative therapies (trend of our times?)"*
- *"Written material to support deprescribing."*
- *"Training and information on the non-use of BZRA for sleep problems as a first option to other services different from Primary Care, such as Emergency and specialized hospital services. – Information"*
- *"Public education about the extreme addiction potential of these drugs."*
- *"Good doctor-patient communication. It would be helpful if BZRA would require a similar prescription as narcotic medication."*
