# Supplementary material for: Barriers to Deprescribing Benzodiazepines in Older Adults in a Survey of European Physicians
Source: JAMA Netw Open. 2025 Mar 3;8(3):e2459883. doi: 10.1001/jamanetworkopen.2024.59883 (PMC11877185; doi:10.1001/jamanetworkopen.2024.59883)
Supplement: Supplement 2. — Data Sharing Statement [file jamanetwopen-e2459883-s002.pdf]

Data Sharing Statement

Shapoval. Barriers to Deprescribing Benzodiazepines in Older Adults in a Survey of European Physicians. *JAMA Netw Open*. Published March 03, 2025.
doi:10.1001/jamanetworkopen.2024.59883

Data
Data available: Yes
Data types: Deidentified participant data, Data dictionary
How to access data: The data dictionary is accessible on the Open Science Framework (OSF) platform via the following links: for physicians https://osf.io/eqfsw and general practitioners https://osf.io/hav9e The data can be obtained by contacting the researchers at vladyslav.shapoval@uclouvain.be or anne.spinewine@uclouvain.be
When available: With publication

Supporting Documents
Document types: None

Additional Information
Who can access the data: The data will be provided to anyone who requests it and specifies the purpose of their data analysis.
Types of analyses: There will be no restrictions on the type of analysis for which the data can be used, nor any other limitations on access.
Mechanisms of data availability: After the request by mentioned above e-mails and presenting the purpose, the data will be shared with a person requesting it;
Any additional restrictions: No additional restrictions.
